# Supplementary material for: Characteristics and outcomes of patients with COVID-19 with and without prevalent hypertension: a multinational cohort study
Source: BMJ Open. 2021 Dec 22;11(12):e057632. doi: 10.1136/bmjopen-2021-057632 (PMC8704062; doi:10.1136/bmjopen-2021-057632)
Supplement: Supplementary data [file bmjopen-2021-057632supp001.pdf]

Supporting Figure 1. Flowchart showing the selection of databases included in the analyses

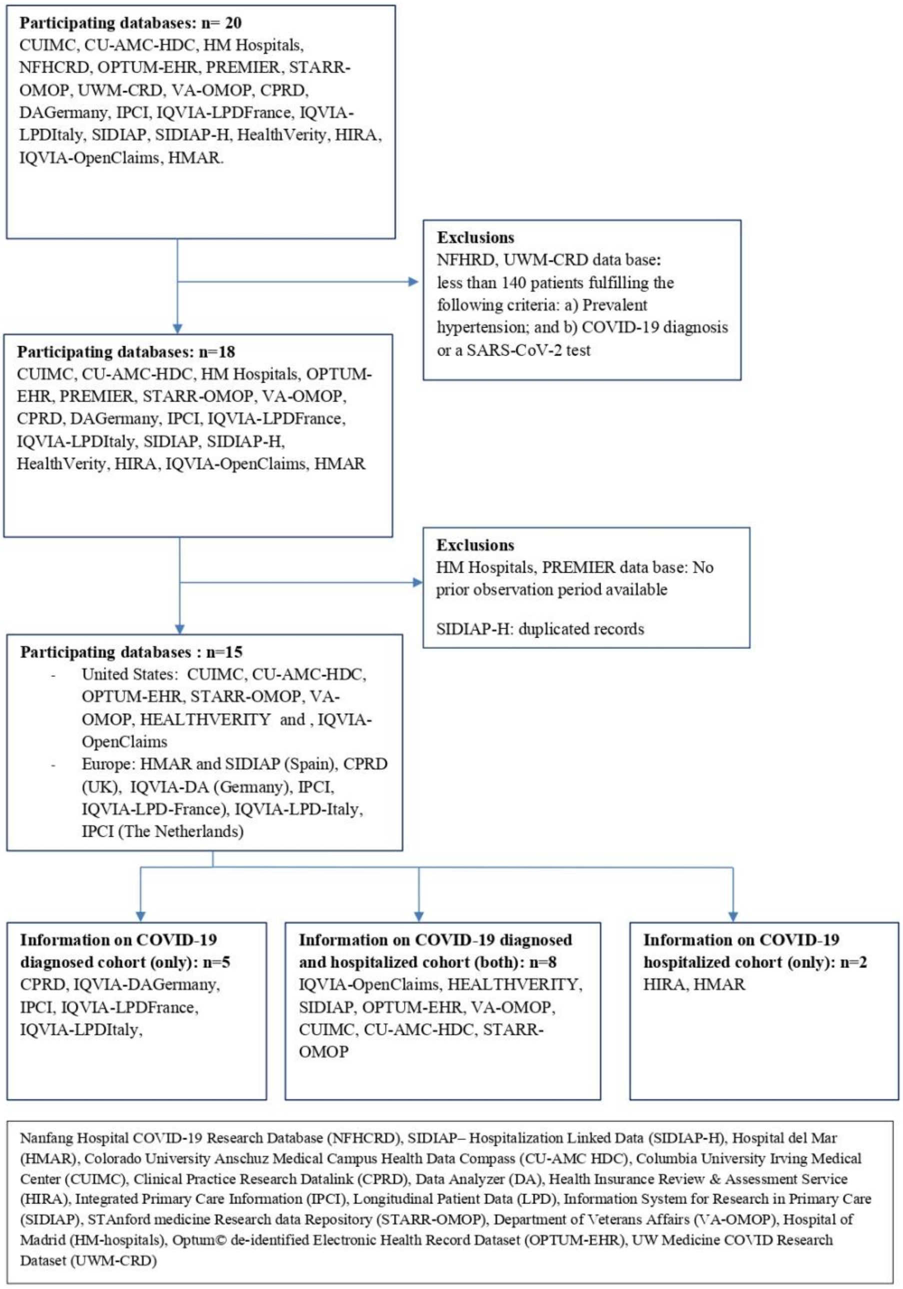

Reyes C, *et al. BMJ Open* 2021; 11:e057632. doi: 10.1136/bmjopen-2021-057632

Supporting Table 1. Description of included databases

Institution Name/ Database	Database Description	Country
Janssen Research & Development<br>The Clinical Practice Research Datalink (CPRD)	The Clinical Practice Research Datalink (CPRD) is a governmental not-for-profit research service jointly funded by the NHS National Institute for Health Research (NIHR) and the Medicines and Healthcare products Regulatory Agency (MHRA) a part of the Department of Health United Kingdom (UK). CPRD consists of data collected from UK primary care for all ages. This includes conditions observations measurements and procedures that the general practitioner is made aware of in addition to any prescriptions as prescribed by the general practitioner. In addition to primary care there are also linked secondary care records for a small number of people. The major data elements contained within this database are outpatient prescriptions given by the general practitioner (coded with Multilex codes) and outpatient clinical referral immunization or test events that the general practitioner knows about (coded in Read or ICD10 or LOINC codes). The database also contains the patients' year of births and any date of deaths.	United Kingdom
IDIAPJGol<br>The Information System for Research in Primary Care (SIDIAP)	The Information System for Research in Primary Care (SIDIAP; www.sidiap.org) is a primary care records database that covers approximately 80% of the population of Catalonia North-East Spain. Healthcare is universal and tax-payer funded in the region and primary care physicians are gatekeepers for all care and responsible for repeat prescriptions.	Spain
Stanford Medicine Research Data Repository (STARR-OMOP)	A clinical data warehouse containing live Epic data from Stanford Health Care the Stanford Children's Hospital	United States

	the University Healthcare Alliance and Packard Children's Health Alliance clinics.<br>Reference: Datta S Posada J Olson G *et al.* A new paradigm for accelerating clinical data science at Stanford Medicine. *arXiv* 2020; published online March 17. http://arxiv.org/abs/2003.10534 (accessed Aug 20 2020).	
Columbia University Irving Medical Center (CUIMC)	The clinical data warehouse of New York-Presbyterian Hospital/Columbia University Irving Medical Center New York NY based on its current and previous electronic health record systems with data spanning over 30 years and including over 6 million patients	United States
IQVIA<br>Open Claims	Pre-adjudicated claims covering over 300 Million lives (~80% of the US) collected from office-based physicians and specialists via office management software and clearinghouse switch sources for the purpose of reimbursement.	United States
HIRA<br>Health Insurance Review & Assessment Service	National claim data from a single insurance service from South Korea, It contains the observational medical records (including both inpatient and outpatient) of a patient while they are qualified to get the national medical insurance.	South Korea
HMAR<br>Hospital del mar	Anonymized data from the Electronic Medical Records from Hospital del Mar (Barcelona, Spain). Hospital belonging to the Spanish National Health System (public), attending the Eastern area of Barcelona City. Includes hospital data collected routinely in the clinical practice, both structured and unstructured information, extracted using a free text analysis tool (with natural language processing): Inpatient (hospital) care, Outpatient specialist care, Emergency Room Visits and partial information from other settings like primary care and pharmacy care present in free text notes from EMRs. All subjects with at least one healthcare encounter with the Hospital within approximately last 20 years are included (approximately 0.6 M subjects, with more than	Spain

	5 M hospitalizations/visits). Hospital del Mar data are made available through collaboration with TFS / IOMED.	
OPTUM-EHR<br>Optum® de-identified Electronic Health Record Dataset	Optum ® de-identified Electronic Health Record Dataset is derived from dozens of healthcare provider organizations in the United States (that include more than 700 hospitals and 7,000 Clinics treating more than 103 million patients) receiving care in the United States. The medical record data includes clinical information, inclusive of prescriptions as prescribed and administered, lab results, vital signs, body measurements, diagnoses, procedures, and information derived from clinical Notes using Natural Language Processing (NLP)	United States
IPCI<br>Integrated Primary Care Information	The Integrated Primary Care Information (IPCI) database is collected from EHR records of patients registered with 391 GPs throughout the Netherlands. The database contains records from approximately 2.6 million patients out of a Dutch population of 17M (8.2%) starting in 1996.	The Netherlands
DA Germany<br>IQVIA Disease Analyser Germany	IQVIA DA Germany is collected from extracts of patient management software used by GPs and specialists practicing in ambulatory care settings. Data coverage includes more than 34M distinct person records out of at total population of 80M (42.5%) in the country and collected from 2,734 providers. Dates of service include from 1992 through March 2020	Germany
LPD-Italy<br>IQVIA LPD Italy	LPD Italy is comprised of anonymised patient records collected from software used by GPs during an office visit to document patients' clinical records. Data coverage includes over 2M patient records with at least one visit and 119.5M prescription orders across 900 GP practices. Dates of service include from 2004 through	Italy

	present. Observation time is defined by the first and last consultation dates. Drugs are captured as prescription records with product, quantity, dosing directions, strength, indication and date of consultation.	
LPD-France	LPD France is a computerised network of physicians including GPs who contribute to a centralised database of anonymised patient EMR. Currently, >1200 GPs from 400 practices are contributing to the database covering 7.8M patients in France. The database covers a time period from 1994 through the present. Observation time is defined by the first and last consultation dates. Drug information is derived from GP prescriptions. Drugs obtained over the counter by the patient outside the prescription system are not reported	France
HEALTHVERITY	This HealthVerity derived data set contains de-identified patient information with an antibody and/or diagnostic test for COVID-19 linked to all available Medical Claims and Pharmacy Data from select private data providers participating in the HealthVerity marketplace.	United States
University of Colorado Anschuz Medical Campus Health Data Compass (CU-AMC HDC)	Health Data Compass (HDC) is a multi-institutional data warehouse. HDC contains inpatient and outpatient electronic medical data including patient, encounter, diagnosis, procedures, medications, laboratory results from two electronic medical record systems (UCHealth and Children's Hospital of Colorado), state-level all-payers claims data, and the Colorado death registry. Acknowledgement statement: Supported by the Health Data Compass Data Warehouse project (healthdatacompass.org)	
Department of Veterans Affairs<br>VA- OMOP	VA-OMOP data reflects the national Department of Veterans Affairs health care system which is the largest integrated provider of medical and mental health services	United States

	in the United States. Care is provided at 170 VA Medical Centers and 1 063 outpatient sites serving more than 9 million enrolled Veterans each year.	

Supporting Table 2. Definitions and codes used to identify COVID-19 cases

The below tables summarises the concepts used to identify patients diagnosed with COVID-19 . The full description of the logic used to identify patients diagnosed and hospitalized is provided at https://atlas.ohdsi.org/#/cohortdefinition/200 and https://atlas.ohdsi.org/#/cohortdefinition/197 respectively.

**COVID-19 condition codes**		
**Id**	**Name**	**Vocabulary**
756023	Acute bronchitis due to COVID-19	OMOP Extension
756044	Acute respiratory distress syndrome (ARDS) due to COVID-19	OMOP Extension
756061	Asymptomatic COVID-19	OMOP Extension
756031	Bronchitis due to COVID-19	OMOP Extension
439676	Coronavirus infection	SNOMED
37311061	Disease caused by 2019-nCoV	SNOMED
4100065	Disease due to Coronaviridae	SNOMED
37310284	Encephalopathy caused by 2019 novel coronavirus	SNOMED

37310283	Gastroenteritis caused by 2019 novel coronavirus	SNOMED
4248811	Healthcare associated severe acute respiratory syndrome	SNOMED
756081	Infection of lower respiratory tract due to COVID-19	OMOP Extension
37310286	Infection of upper respiratory tract caused by 2019 novel coronavirus	SNOMED
45763594	Middle East respiratory syndrome	SNOMED
37310287	Myocarditis caused by 2019 novel coronavirus	SNOMED
37310254	Otitis media caused by 2019 novel coronavirus	SNOMED
37310285	Pneumonia caused by 2019 novel coronavirus	SNOMED
37016927	Pneumonia caused by Human coronavirus	SNOMED
40479642	Pneumonia due to Severe acute respiratory syndrome coronavirus	SNOMED
756039	Respiratory infection due to COVID-19	OMOP Extension
320651	Severe acute respiratory syndrome	SNOMED
37396171	Severe acute respiratory syndrome of upper respiratory tract	SNOMED
37311060	Suspected disease caused by 2019-nCoV	SNOMED
**COVID-19 specific testing - Positive**		
37310282	2019 novel coronavirus detected	SNOMED
**COVID-19 specific testing (note these required a corresponding value as concept of: Detected Positive or Present)**		

BMJ Publishing Group Limited (BMJ) disclaims all liability and responsibility arising from any reliance placed on this supplemental material which has been supplied by the author(s)

37310255	Detection of 2019 novel coronavirus using polymerase chain reaction technique	SNOMED
700360	Infectious agent detection by nucleic acid (DNA or RNA); severe acute respiratory syndrome coronavirus 2 (SARS-CoV-2) (Coronavirus disease [COVID-19]) amplified probe technique	CPT4
37310258	Measurement of 2019 novel coronavirus antibody	SNOMED
37310257	Measurement of 2019 novel coronavirus antigen	SNOMED
756055	Measurement of Severe acute respiratory syndrome coronavirus 2 (SARS-CoV-2)	OMOP Extension
586310	Measurement of Severe acute respiratory syndrome coronavirus 2 (SARS-CoV-2) Genetic material using Molecular method	OMOP Extension
704991	Measurement of Severe acute respiratory syndrome coronavirus 2 (SARS-CoV-2) in Blood	OMOP Extension
756029	Measurement of Severe acute respiratory syndrome coronavirus 2 (SARS-CoV-2) in Respiratory specimen	OMOP Extension
586307	Measurement of Severe acute respiratory syndrome coronavirus 2 (SARS-CoV-2) in Saliva	OMOP Extension
705107	Measurement of Severe acute respiratory syndrome coronavirus 2 (SARS-CoV-2) in Sample from nose	OMOP Extension
586309	Measurement of Severe acute respiratory syndrome coronavirus 2 (SARS-CoV-2) in Specified specimen	OMOP Extension
756065	Measurement of Severe acute respiratory syndrome coronavirus 2 (SARS-CoV-2) in Unspecified specimen	OMOP Extension
704992	Measurement of Severe acute respiratory syndrome coronavirus 2 (SARS-CoV-2) using Culture method	OMOP Extension
705001	Measurement of Severe acute respiratory syndrome coronavirus 2 (SARS-CoV-2) using Nucleic acid amplification technique	OMOP Extension
705000	Measurement of Severe acute respiratory syndrome coronavirus 2 (SARS-CoV-2) using Nucleic acid amplification technique in Blood	OMOP Extension
756085	Measurement of Severe acute respiratory syndrome coronavirus 2 (SARS-CoV-2) using Nucleic acid amplification technique in Respiratory specimen	OMOP Extension
586308	Measurement of Severe acute respiratory syndrome coronavirus 2 (SARS-CoV-2) using Nucleic acid amplification technique in Saliva	OMOP Extension
705106	Measurement of Severe acute respiratory syndrome coronavirus 2 (SARS-CoV-2) using Nucleic acid amplification technique in Sample from nose	OMOP Extension
756084	Measurement of Severe acute respiratory syndrome coronavirus 2 (SARS-CoV-2) using Nucleic acid amplification technique in Unspecified specimen	OMOP Extension
704993	Measurement of Severe acute respiratory syndrome coronavirus 2 (SARS-CoV-2) using Sequencing	OMOP Extension

Reyes C, *et al. BMJ Open* 2021; 11:e057632. doi: 10.1136/bmjopen-2021-057632

586516	SARS-CoV-2 (COVID19) [Presence] in Unspecified specimen by Organism specific culture	LOINC
723480	SARS-CoV-2 (COVID19) Ab [Interpretation] in Serum or Plasma	LOINC
586515	SARS-CoV-2 (COVID19) Ab [Presence] in Serum or Plasma by Immunoassay	LOINC
586522	SARS-CoV-2 (COVID19) Ab [Units/volume] in Serum or Plasma by Immunoassay	LOINC
706179	SARS-CoV-2 (COVID19) Ab panel - Serum or Plasma by Immunoassay	LOINC
723477	SARS-CoV-2 (COVID19) Ag [Presence] in Respiratory specimen by Rapid immunoassay	LOINC
706166	SARS-CoV-2 (COVID19) E gene [Cycle Threshold #] in Unspecified specimen by NAA with probe detection	LOINC
586523	SARS-CoV-2 (COVID19) E gene [Presence] in Respiratory specimen by NAA with probe detection	LOINC
586518	SARS-CoV-2 (COVID19) E gene [Presence] in Serum or Plasma by NAA with probe detection	LOINC
706174	SARS-CoV-2 (COVID19) E gene [Presence] in Unspecified specimen by NAA with probe detection	LOINC
723473	SARS-CoV-2 (COVID19) IgA Ab [Presence] in Serum or Plasma by Immunoassay	LOINC
586521	SARS-CoV-2 (COVID19) IgA Ab [Presence] in Serum Plasma or Blood by Rapid immunoassay	LOINC
723459	SARS-CoV-2 (COVID19) IgA Ab [Units/volume] in Serum or Plasma by Immunoassay	LOINC
757686	SARS-CoV-2 (COVID19) IgA+IgM [Presence] in Serum or Plasma by Immunoassay	LOINC
586527	SARS-CoV-2 (COVID19) IgG Ab [Presence] in DBS by Immunoassay	LOINC
723474	SARS-CoV-2 (COVID19) IgG Ab [Presence] in Serum or Plasma by Immunoassay	LOINC
706181	SARS-CoV-2 (COVID19) IgG Ab [Presence] in Serum Plasma or Blood by Rapid immunoassay	LOINC
706177	SARS-CoV-2 (COVID19) IgG Ab [Units/volume] in Serum or Plasma by Immunoassay	LOINC
706176	SARS-CoV-2 (COVID19) IgG and IgM panel - Serum Plasma or Blood by Rapid immunoassay	LOINC
723479	SARS-CoV-2 (COVID19) IgG+IgM Ab [Presence] in Serum or Plasma by Immunoassay	LOINC

723475	SARS-CoV-2 (COVID19) IgM Ab [Presence] in Serum or Plasma by Immunoassay	LOINC
706180	SARS-CoV-2 (COVID19) IgM Ab [Presence] in Serum Plasma or Blood by Rapid immunoassay	LOINC
706178	SARS-CoV-2 (COVID19) IgM Ab [Units/volume] in Serum or Plasma by Immunoassay	LOINC
706167	SARS-CoV-2 (COVID19) N gene [Cycle Threshold #] in Unspecified specimen by NAA with probe detection	LOINC
706157	SARS-CoV-2 (COVID19) N gene [Cycle Threshold #] in Unspecified specimen by Nucleic acid amplification using CDC primer-probe set N1	LOINC
706155	SARS-CoV-2 (COVID19) N gene [Cycle Threshold #] in Unspecified specimen by Nucleic acid amplification using CDC primer-probe set N2	LOINC
715272	SARS-CoV-2 (COVID19) N gene [Presence] in Nasopharynx by NAA with probe detection	LOINC
757678	SARS-CoV-2 (COVID19) N gene [Presence] in Nose by NAA with probe detection	LOINC
706161	SARS-CoV-2 (COVID19) N gene [Presence] in Respiratory specimen by NAA with probe detection	LOINC
586524	SARS-CoV-2 (COVID19) N gene [Presence] in Respiratory specimen by Nucleic acid amplification using CDC primer-probe set N1	LOINC
586525	SARS-CoV-2 (COVID19) N gene [Presence] in Respiratory specimen by Nucleic acid amplification using CDC primer-probe set N2	LOINC
586520	SARS-CoV-2 (COVID19) N gene [Presence] in Serum or Plasma by NAA with probe detection	LOINC
706175	SARS-CoV-2 (COVID19) N gene [Presence] in Unspecified specimen by NAA with probe detection	LOINC
706156	SARS-CoV-2 (COVID19) N gene [Presence] in Unspecified specimen by Nucleic acid amplification using CDC primer-probe set N1	LOINC
706154	SARS-CoV-2 (COVID19) N gene [Presence] in Unspecified specimen by Nucleic acid amplification using CDC primer-probe set N2	LOINC
757680	SARS-CoV-2 (COVID19) neutralizing antibody [Presence] in Serum by pVNT	LOINC
757679	SARS-CoV-2 (COVID19) neutralizing antibody [Titer] in Serum by pVNT	LOINC
723469	SARS-CoV-2 (COVID19) ORF1ab region [Cycle Threshold #] in Respiratory specimen by NAA with probe detection	LOINC
706168	SARS-CoV-2 (COVID19) ORF1ab region [Cycle Threshold #] in Unspecified specimen by NAA with probe detection	LOINC
723478	SARS-CoV-2 (COVID19) ORF1ab region [Presence] in Respiratory specimen by NAA with probe detection	LOINC

723464	SARS-CoV-2 (COVID19) ORF1ab region [Presence] in Unspecified specimen by NAA with probe detection	LOINC
723471	SARS-CoV-2 (COVID19) RdRp gene [Cycle Threshold #] in Respiratory specimen by NAA with probe detection	LOINC
723470	SARS-CoV-2 (COVID19) RdRp gene [Cycle Threshold #] in Unspecified specimen by NAA with probe detection	LOINC
706160	SARS-CoV-2 (COVID19) RdRp gene [Presence] in Respiratory specimen by NAA with probe detection	LOINC
706173	SARS-CoV-2 (COVID19) RdRp gene [Presence] in Unspecified specimen by NAA with probe detection	LOINC
586528	SARS-CoV-2 (COVID19) RNA [Cycle Threshold #] in Respiratory specimen by NAA with probe detection	LOINC
586529	SARS-CoV-2 (COVID19) RNA [Cycle Threshold #] in Unspecified specimen by NAA with probe detection	LOINC
715262	SARS-CoV-2 (COVID19) RNA [Log #/volume] (viral load) in Unspecified specimen by NAA with probe detection	LOINC
723476	SARS-CoV-2 (COVID19) RNA [Presence] in Nasopharynx by NAA with non-probe detection	LOINC
586526	SARS-CoV-2 (COVID19) RNA [Presence] in Nasopharynx by NAA with probe detection	LOINC
757677	SARS-CoV-2 (COVID19) RNA [Presence] in Nose by NAA with probe detection	LOINC
706163	SARS-CoV-2 (COVID19) RNA [Presence] in Respiratory specimen by NAA with probe detection	LOINC
715260	SARS-CoV-2 (COVID19) RNA [Presence] in Saliva (oral fluid) by NAA with probe detection	LOINC
715261	SARS-CoV-2 (COVID19) RNA [Presence] in Saliva (oral fluid) by Sequencing	LOINC
723463	SARS-CoV-2 (COVID19) RNA [Presence] in Serum or Plasma by NAA with probe detection	LOINC
706170	SARS-CoV-2 (COVID19) RNA [Presence] in Unspecified specimen by NAA with probe detection	LOINC
706158	SARS-CoV-2 (COVID19) RNA panel - Respiratory specimen by NAA with probe detection	LOINC
706169	SARS-CoV-2 (COVID19) RNA panel - Unspecified specimen by NAA with probe detection	LOINC
723467	SARS-CoV-2 (COVID19) S gene [Cycle Threshold #] in Respiratory specimen by NAA with probe detection	LOINC
723468	SARS-CoV-2 (COVID19) S gene [Cycle Threshold #] in Unspecified specimen by NAA with probe detection	LOINC

Reyes C, *et al. BMJ Open* 2021; 11:e057632. doi: 10.1136/bmjopen-2021-057632

723465	SARS-CoV-2 (COVID19) S gene [Presence] in Respiratory specimen by NAA with probe detection	LOINC
586519	SARS-CoV-2 (COVID19) S gene [Presence] in Serum or Plasma by NAA with probe detection	LOINC
723466	SARS-CoV-2 (COVID19) S gene [Presence] in Unspecified specimen by NAA with probe detection	LOINC
586517	SARS-CoV-2 (COVID19) whole genome [Nucleotide sequence] in Isolate by Sequencing	LOINC
40218805	Testing for SARS-CoV-2 in CDC laboratory	HCPCS
40218804	Testing for SARS-CoV-2 in non-CDC laboratory	HCPCS

Supporting Table 3. Definitions and codes used for hypertension and other comorbidities

**Name**	**Included Codes**
Hyperlipidemia	https://atlas.ohdsi.org/#/concept/432867
Chronic kidney disease	https://atlas.ohdsi.org/#/cohortdefinition/312
Cancer	https://atlas.ohdsi.org/#/cohortdefinition/222
Asthma	https://atlas.ohdsi.org/#/cohortdefinition/218
Dementia	https://atlas.ohdsi.org/#/cohortdefinition/226
Total cardiovascular disease	https://atlas.ohdsi.org/#/cohortdefinition/246
Peripheral vascular disease	https://atlas.ohdsi.org/#/concept/321052

Cerebrovascular disease	https://atlas.ohdsi.org/#/concept/381591
Chronic liver disease	https://atlas.ohdsi.org/#/concept/4212540
Chronic obstructive pulmonary disease	https://atlas.ohdsi.org/#/cohortdefinition/219
Heart disease	https://atlas.ohdsi.org/#/cohortdefinition/231
Hypertension	https://atlas.ohdsi.org/#/cohortdefinition/227
Obesity	https://atlas.ohdsi.org/#/cohortdefinition/224
Type 2 Diabetes Mellitus	https://atlas.ohdsi.org/#/cohortdefinition/311

Supporting Table 4. Prevalence of hypertension among COVID-19 patients in the diagnosed and hospitalised cohorts in the CHARYBDIS Network.

	**Diagnosed with COVID-19**		**Hospitalized with COVID-19**	
	**N of prevalent cases**	**% (95%CI)**	**N of prevalent cases**	**% (95%CI)**
IQVIA-OpenClaims-US	1,245,436	48.3 (48.2-48.4)	384,508	76.5 (76.3-76.6)
OPTUM-EHR-US	66,432	37.4 (37.2-37.7)	18,242	64.1 (63.5-64.6)
VA-OMOP-US	34,093	61.4 (61.0-61.8)	8,996	85.9 (85.2-86.6)

Reyes C, *et al. BMJ Open* 2021; 11:e057632. doi: 10.1136/bmjopen-2021-057632

BMJ Publishing Group Limited (BMJ) disclaims all liability and responsibility arising from any reliance placed on this supplemental material which has been supplied by the author(s)

HEALTHVERITY-US	25,405	22.3 (22.0-22.5)	4,512	59.5 (58.4-60.6)
SIDIAP-Spain	21,289	17.4 (17.2-17.6)	5,636	31.0 (30.3-31.6)
CUIMC-US	3,672	43.1 (42.1-44.2)	1,708	65.7 (63.9-67.5)
IQVIA-LPD-France	3,260	19.0 (18.4-19.6)	-	-
CU-AMC-HDC-US	2,461	33.9 (32.8-34.9)	904	63.0 (60.5-65.5)
IQVIA-DA-Germany	2,418	30.3 (29.3-31.3)	-	-
HIRA-South Korea	-	-	1943	25.6 (24.6-26.6)
IQVIA-LPD-Italy	1,618	36.1 (34.6-37.5)	-	-
STARR-OMOP-US	1,246	37.4 (35.8-39.1)	342	55.6 (51.7-59.5)
HMAR-Spain	-	-	594	29.5 (27.5-31.5)
CPRD-UK	756	22.4 (21.0-23.8)	-	-
IPCI-The Netherlands	676	22.2 (20.7-23.7)	-	-
