# Supplementary material for: Characteristics and outcomes of patients with COVID-19 with and without prevalent hypertension: a multinational cohort study
Source: BMJ Open. 2021 Dec 22;11(12):e057632. doi: 10.1136/bmjopen-2021-057632 (PMC8704062; doi:10.1136/bmjopen-2021-057632)
Supplement: Author's manuscript [file bmjopen-2021-057632.draft_revisions.pdf]

BMJ Open is committed to open peer review. As part of this commitment we make the peer review history of every article we publish publicly available.

When an article is published we post the peer reviewers' comments and the authors' responses online. We also post the versions of the paper that were used during peer review. These are the versions that the peer review comments apply to.

The versions of the paper that follow are the versions that were submitted during the peer review process. They are not the versions of record or the final published versions. They should not be cited or distributed as the published version of this manuscript.

BMJ Open is an open access journal and the full, final, typeset and author-corrected version of record of the manuscript is available on our site with no access controls, subscription charges or pay-per-view fees (http://bmjopen.bmj.com).

If you have any questions on BMJ Open's open peer review process please email info.bmjopen@bmj.com

BMJ Open

Characteristics and outcomes of COVID-19 patients with and without prevalent hypertension: a multinational cohort study

Journal:	*BMJ Open*
Manuscript ID	bmjopen-2021-057632
Article Type:	Original research
Date Submitted by the Author:	29-Sep-2021
Complete List of Authors:	Reyes, Carlen; GREMPAL Research Group, Fundació Institut Universitari per a la recerca a l'Atenció Primària de Salut Jordi Gol i Gurina (IDIAPJGol), and CIBERFes, Universitat Autonoma de Barcelona and Instituto de Salud Carlos III,<br>Pistillo, Andrea ; Fundació Institut Universitari per a la recerca a l'Atenció Primària de Salut Jordi Gol i Gurina (IDIAPJGol),<br>Fernández-Bertolín, Sergio; Fundació Institut Universitari per a la recerca a l'Atenció Primària de Salut Jordi Gol i Gurina (IDIAPJGol),<br>Recalde, Martina<br>Roel, Elena<br>Puente, Diana; IDIAP Jordi Gol, Research<br>Sena, Anthony<br>Blacketer, Clair<br>Lai, Lana<br>Alshammari, Thamir; King Saud University, Medication Safety Research Chair; Saudi Food and Drug Authority,<br>Ahmed, Waheed-UI-Rahman<br>Alser, Osaid ; Harvard Medical School, Trauma, Emergency Surgery and Surgical Critical Care<br>Alghoul, Heba; Islamic University of Gaza Faculty of Medicine,<br>Areia, Carlos; University of Oxford, Nuffield Department of Clinical Neurosciences<br>Dawoud, Dalia; National Institute for Health and Care Excellence,<br>Prats-Uribe, Albert; University of Oxford, Nuffield Department of Orthopaedics Rheumatology and Musculoskeletal Science<br>Valveny, Neus<br>de Maeztu, Gabriel<br>Sorlí Redó, Luisa<br>Martinez Roldan, Jordi<br>Lopez Montesinos, Inmaculada<br>Schilling, Lisa<br>Golozar, Asieh; Johns Hopkins University Bloomberg School of Public Health<br>Reich, Christian<br>Posada, Jose<br>Shah, Nigam ; Stanford University,<br>You, Sen Chang<br>Lynch, Kristine; Department of Veterans Affairs; The University of Utah School of Medicine

	DuVall, Scott; Department of Veterans Affairs; The University of Utah School of Medicine<br>Matheny, Michael; VA Tennessee Valley Healthcare System, GRECC; Vanderbilt University Medical Center, Department of Biomedical Informatics<br>Nyberg, Fredrik; University of Gothenburg Sahlgrenska Academy, School of Public Health and Community Medicine, Institute of Medicine, Institute of Medicine<br>Ostropolets, Anna<br>Hripcsak, George<br>Rijnbeek, P; Erasmus Medical Center, Rotterdam<br>Suchard, MA; University of California Los Angeles,<br>Ryan, Patrick; Janssen Research and Development LLC, Observational Health Data Analytics; Columbia University Irving Medical Center, Department of Biomedical Informatics<br>Kostka, Kristin<br>Duarte-Salles, Talita; Institut de Recerca en Atencio Primaria Jordi Gol,

Keywords:	COVID-19, EPIDEMIOLOGY, Hypertension < CARDIOLOGY

SCHOLARONE™
Manuscripts

I, the Submitting Author has the right to grant and does grant on behalf of all authors of the Work (as defined in the below author licence), an exclusive licence and/or a non-exclusive licence for contributions from authors who are: i) UK Crown employees; ii) where BMJ has agreed a CC-BY licence shall apply, and/or iii) in accordance with the terms applicable for US Federal Government officers or employees acting as part of their official duties; on a worldwide, perpetual, irrevocable, royalty-free basis to BMJ Publishing Group Ltd ("BMJ") its licensees and where the relevant Journal is co-owned by BMJ to the co-owners of the Journal, to publish the Work in this journal and any other BMJ products and to exploit all rights, as set out in our licence.

The Submitting Author accepts and understands that any supply made under these terms is made by BMJ to the Submitting Author unless you are acting as an employee on behalf of your employer or a postgraduate student of an affiliated institution which is paying any applicable article publishing charge ("APC") for Open Access articles. Where the Submitting Author wishes to make the Work available on an Open Access basis (and intends to pay the relevant APC), the terms of reuse of such Open Access shall be governed by a Creative Commons licence – details of these licences and which Creative Commons licence will apply to this Work are set out in our licence referred to above.

Other than as permitted in any relevant BMJ Author's Self Archiving Policies, I confirm this Work has not been accepted for publication elsewhere, is not being considered for publication elsewhere and does not duplicate material already published. I confirm all authors consent to publication of this Work and authorise the granting of this licence.

For peer review only

Characteristics and outcomes of COVID-19 patients with and without prevalent hypertension: a multinational cohort study

Carlen Reyes[1], Andrea Pistillo[1], Sergio Fernandez Bertolin[1], Martina Recalde[1,2], Elena Roel[1,2], Diana Puente[1,2], Anthony G. Sena[3,4], Claire Blacketer[3,4], Lana YH Lai[5], Thamer M Alshammari[6], Waheed-UI-Rahman Ahmed[7,8], Osaid Alser[9], Heba Alghoul[10], Carlos Areia[11], Dalia Dawoud[12,13], Albert Prats-Uribe[14], Neus Valveny[15], Gabriel de Maeztu[16], Luisa Sorlí Redó[2,17,18], Jordi Martínez Roldán[19], Inmaculada Lopez Montesinos[17], Lisa M Schilling[20], Asieh Golozar[21,22], Christian Reich[23], Jose D. Posada[24], Nigam H. Shah[24], Seng Chan You[25], Kristine E. Lynch[26,27], Scott L. DuVall[26,27], Michael Matheny[26,27], Fredrik Nyberg[28], Anna Ostropolets[29], George Hripcsak[30,31], Peter Rijnbeek[32], Mark A. Suchard[33], Patrick Ryan[3,30], Kristin Kostka[23,34], Talita Duarte-Salles[1*]

Affiliations:

1- Fundació Institut Universitari per a la recerca a l'Atenció Primària de Salut Jordi Gol i Gurina (IDIAPJGol), Barcelona, Spain.

2- Universitat Autònoma de Barcelona, Spain

3- Janssen Research & Development, Titusville, NJ, USA

4- Department of Medical Informatics, Erasmus University Medical Center, Rotterdam, The Netherlands

5- School of Medical Sciences, University of Manchester, UK

6- College of Pharmacy, Riyadh Elm University Riyadh, Saudi

7- Nuffield Department of Orthopaedics, Rheumatology, and Musculoskeletal Sciences, University of Oxford, Botnar Research Centre, Windmill Road, Oxford, UK.

For peer review only - http://bmjopen.bmj.com/site/about/guidelines.xhtml

8- College of Medicine and Health, University of Exeter, St Luke’s Campus, Heavitree Road, Exeter, UK
9- Massachusetts General Hospital, Harvard Medical School, USA
10- Faculty of Medicine, Islamic University of Gaza, Palestine
11- Nuffield Department of Clinical Neurosciences, University of Oxford, Oxford, UK
12- National Institute for Health and Care Excellence (NICE), London, UK
13- Cairo University, Faculty of Pharmacy, Cairo, Egypt
14- Centre for Statistics in Medicine, NDORMS, University of Oxford, Botnar Research Centre, Nuffield Orthopaedic Centre, Windmill Road, Oxford, UK
15- Real-World Evidence, TFS, Barcelona, Spain
16- IOMED, Barcelona, Spain
17- Department of Infectious Diseases, Hospital del Mar, Institut Hospital del Mar d'Investigació Mèdica (IMIM), Barcelona, Spain
18- Universitat Pompeu Fabra, Barcelona, Spain
19- Director of Innovation and Digital Transformation, Hospital del Mar, Barcelona, Spain
20- University of Colorado Anschutz Medical Campus, Aurora, CO, USA
21- Regeneron Pharmaceuticals, Tarrytown, NY, USA
22- Johns Hopkins Bloomberg School of Public health, NY, USA
23- Real-World Solutions, IQVIA, Cambridge, MA, USA
24- Stanford University School of Medicine, Stanford, CA, USA
25- Department of Preventive Medicine, Yonsei University College of Medicine, Seoul, Korea
26- VA Informatics and Computing Infrastructure, VA Salt Lake City Health Care System, Salt Lake City, UT, USA

For peer review only

27- Department of Internal Medicine, University of Utah School of Medicine, Salt Lake City, UT, USA

28- School of Public Health and Community Medicine, Institute of Medicine, Sahlgrenska Academy, University of Gothenburg, Gothenburg, Sweden

29- Columbia University Irving Medical Center, New York, USA

30- Department of Biomedical Informatics, Columbia University Irving Medical Center, New York, NY, USA

31- Medical Informatics Services, NewYork-Presbyterian Hospital, New York, NY, USA

32- Department of Medical Informatics Erasmus University Medical Center, Rotterdam, The Netherlands

33- Department of Biostatistics, Fielding School of Public Health, University of California, Los Angeles, USA

34- The OHDSI Center at the Roux Institute, Northeastern University, Portland, ME, USA

*Corresponding author:

Talita Duarte-Salles

Fundació Institut Universitari per la recerca a L'Atenció Primària de Salut Jordi Gol I Gurina (IDIAPJGol)

Gran Via Corts Catalanes, 587, àtic

08007 Barcelona-Spain

Tel: +34-93 4824342

Email: tduarte@idiapjgol.org

For peer review only

ABSTRACT

Objective: To characterize patients with and without prevalent hypertension and COVID-19, and to assess their adverse outcomes in both in and outpatients.

Design and setting: Retrospective cohort study using 15 healthcare databases (primary and secondary electronic health care records, insurance and national claims data) from the US, Europe and South Korea, standardized to the Observation Medical Outcomes Partnership common data model.

Participants: We included all patients diagnosed/hospitalized with COVID-19 (non-mutually exclusive cohorts) and stratified them by hypertension status. Follow-up was from COVID-19 diagnosis/hospitalization to death, end of the study period, or 30-days.

Outcomes: Demographics, comorbidities, and 30-day outcomes (hospitalization, adverse events or death) were reported.

Results: We identified 2,851,035 diagnosed and 563,708 hospitalized patients with COVID-19. Hypertension was more prevalent in the latter (range (%, 95%CI) across databases 17.4 (17.2-17.6)- 61.4 (61.0-61.8) and 25.6 (24.6-26-6)-85.9 (85.2-86.6). Patients with hypertension diagnosed with COVID-19 were predominantly >50-year-old and female. Patients with hypertension were frequently diagnosed with obesity, heart disease, dyslipidaemia, and diabetes. Compared to patients without hypertension, patients with hypertension had more hospitalizations (range 1.3 (0.4-2.2)- 41.1 (39.5-42.7) vs 1.4 (0.9-1.9)-15.9 (14.9-16.9)) and mortality (0.3(0.1-0.5)-18.5 (15.7-21.3) vs 0.2 (0.2-0.2)-11.8 (10.8-12.8)). Hospitalized patients with hypertension were more likely to have acute respiratory distress syndrome (0.1(0.0-0.2) -65.6 (62.5-68.7) vs 0.1 (0.0-0.2)-54.7 (50.5-58.9)), arrhythmia (0.5 (0.3-0.7)-45.8 (42.6-49.0) vs 0.4 (0.3-0.5)-36.8 (32.7-40.9)) and increased

For peer review only

mortality (1.8 (0.4-3.2)-25.1 (23.0-27.2) vs 0.7 (0.5-0.9)-10.9 (10.4-11.4)) than patients without hypertension.

Conclusions: COVID-19 patients with hypertension were more likely to suffer severe outcomes, hospitalizations and deaths compared to those without hypertension.

KEY WORDS: COVID-19, Epidemiology, Hypertension

WORD COUNT: 2,971

ARTICLE SUMMARY

Strengths and limitations of this study

1- This study is unique in its approach to characterizing COVID-19 cases across an international network of healthcare databases, with diverse healthcare systems and policies, through a comprehensive federated approach.
2- This study was carried out using routinely collected clinical practice data, which confers a great external validity, but also implies a risk of misclassification.
3- This study was intentionally descriptive and was deliberately not designed for causal inference.
4- The diagnosed and/or hospitalized cohorts were non-mutually exclusive.
5- The data that underpinned this study mostly came from the initial months of the COVID-19 pandemic and may not be representative of the COVID-19 cases diagnosed and/or hospitalized during subsequent periods.

FUNDING STATEMENT

This work was supported by several funders as follows; The European Health Data & Evidence Network received funding from the Innovative Medicines Initiative 2 Joint Undertaking (JU) under grant agreement No 806968. The JU received support from the European Union's Horizon 2020 research and innovation programme and EFPIA. This research received partial support from the National Institute for Health Research (NIHR)

For peer review only

Oxford Biomedical Research Centre (BRC), US National Institutes of Health (R01 LM006910), US Department of Veterans Affairs, the Health Department from the Generalitat de Catalunya with a grant for research projects on SARS-CoV-2 and COVID-19 disease organized by the Direcció General de Recerca i Innovació en Salut, Janssen Research & Development, TFS, IOMED and IQVIA. The University of Oxford received funding related to this work from the Bill & Melinda Gates Foundation (Investment ID INV-016201 and INV-019257). TFS received funding related to this work from the University of Oxford. This work was also supported with funding, [resources, and facilities] of the Department of Veterans Affairs (VA) Informatics and Computing Infrastructure (VINCI), VA HSR RES 13-457. No funders had a direct role in this study. The views and opinions expressed are those of the authors and do not necessarily reflect those of the Clinician Scientist Award programme, NIHR, Department of Veterans Affairs or the United States Government, NHS, or the Department of Health, England.

COMPETING INTERESTS STATEMENT

SLDV reports grants from Anolinx; MS, reports grants from US National Institutes of Health, grants from Department of Veterans Affairs, during the conduct of the study; grants from IQVIA, personal fees from Janssen Research and Development, grants from US Food and Drug Administration, personal fees from Private Health Management, outside the submitted work LLC, grants from Astellas Pharma, Inc, grants from AstraZeneca Pharmaceuticals LP, grants from Boehringer Ingelheim International GmbH, grants from Celgene Corporation, grants from Eli Lilly and Company, grants from Genentech Inc., grants from Genomic Health, Inc., grants from Gilead Sciences Inc., grants from GlaxoSmithKline PLC, grants from Innocrin Pharmaceuticals Inc., grants from Janssen Pharmaceuticals, Inc., grants from Kantar Health, grants from Myriad Genetic Laboratories, Inc., grants from Novartis International AG, grants from Parexel International Corporation through the University of

For peer review only

For peer review only - http://bmjopen.bmj.com/site/about/guidelines.xhtml

Utah or Western Institute for Veteran Research, outside the submitted work; GH, reports grants from NIH, during the conduct of the study; grants from Janssen Research, outside the submitted work; FN, reports that Until 2019 was an employee of AstraZeneca and holds some AstraZeneca shares, outside the submitted work; KK, reports personal fees from National Institutes of Health, outside the submitted work, and at the time of data analysis and initial drafting of the manuscript, KK was an employee of IQVIA Inc; CR reports he is an employee of IQVIA Inc; GdM is Employee of IOMED; NV is an Employee of TFS; AGS reports personal fees from Janssen R&D, outside the submitted work and full time employee of Janssen R&D and is a Johnson and Johnson shareholder; CB reports personal fees from Janssen R&D, outside the submitted work and is a full time employee of Janssen R&D and is a Johnson and Johnson shareholder; JDP reports grants from National LIbrary of Medicine, during the conduct of the study; AG is an employee of Regeneron Pharmaceuticals and reports stocks from Regeneron Pharmaceuticals. PR reports having received research group grants from Innovative Medicine Initiative and Janssen Research and Development; MS reports grants from US National Institutes of Health, grants from Department of Veterans Affairs, during the conduct of the study; grants from IQVIA, personal fees from Janssen Research and Development, grants from US Food and Drug Administration, personal fees from Private Health Management, outside the submitted work; PR, reports and is an employee of Janssen Research and Development and shareholder of Johnson & Johnson; ER, SFB, NHS, LMS, DP, SCY, MR, APU, HA, KEL, MM, AO, CA, CR, TDS, TMA, OA, W-U-RA, ILM, JMR, LSR, DD, LYHL, AP, have nothing to declare. The views expressed are those of the authors and do not necessarily represent the views or policy of the Department of Veterans Affairs or the United States Government. No other relationships or activities could appear to have influenced the submitted work.

For peer review only - http://bmjopen.bmj.com/site/about/guidelines.xhtml

INTRODUCTION

As of September 2021, the ongoing pandemic of the coronavirus disease 2019 (COVID-19) has affected over 220 million people and the estimated death toll surpasses the 4,5 million deaths worldwide[1]. Hypertension is a common chronic condition that may increase the risk of hospitalizations and adverse outcomes[2]. A higher prevalence of hypertension has been found among COVID-19 patients compared to the general population, which has attracted the attention of researchers[3]. The characterization of this population at risk is key to be able to design effective preventive strategies that could, improve patient outcomes and reduce the pressure on healthcare systems.

To date, observational studies [4-16], systematic reviews, and meta-analyses have reported an increased risk of progression to severe COVID-19 and increased mortality in patients with hypertension [17-21]. However, these studies, either only included hospitalized patients[4-13,15-16], leading to a selection bias, or had a small sample size [6-10,15], both of which limits the extrapolation of results.

Most patients with confirmed SARS-CoV-2 infection, experience mild or moderate symptoms (80%)[22] and are predominantly seen as outpatients, therefore a large characterization study including both inpatient and outpatients is needed.

This study aims to describe and compare the demographics, baseline comorbidities and 30-day outcomes of individuals with COVID-19 and with and without pre-existing hypertension, in both in and outpatients.

MATERIAL AND METHODS

Study design, setting, and data sources

For peer review only - http://bmjopen.bmj.com/site/about/guidelines.xhtml

195 A multinational, multi-data base cohort study was conducted using data from 1st March to the
196 31st October 2020 included in “The Characterizing Health Associated Risks and Your
197 Baseline Disease In SARS-COV-2” (CHARYBDIS [23]) study. This is a large-scale
198 multinational cohort study aimed to characterize health-associated risks and baseline diseases
199 in SARS-COV-2 patients using routinely collected primary care and hospital electronic
200 health records (EHR), hospital billing, and insurance claims data from the United States
201 (US), Europe (the Netherlands, Spain, the United Kingdom (UK), Germany, and France) and
202 Asia (South Korea and China).

203 From the databases contributing to CHARYBDIS, only twenty had available information on
204 pre-existing hypertension and were initially selected. To be included in the study, databases
205 had to: 1. have at least 140 subjects with prevalent hypertension diagnosed with COVID-19
206 (necessary to estimate the prevalence of previous conditions or 30-day outcomes with
207 sufficient precision (confidence interval width of ±5%)) and 2. have at least one year of
208 previous data before the date of COVID-19 diagnosis or hospitalization. Data results for this
209 paper were extracted on the 21st of January 2021 [23]. Fifteen databases complied with the
210 aforementioned inclusion criteria. Of these, five had data for outpatients (IQVIA-
211 Longitudinal Patients Database “LPD” (France), IQVIA-Longitudinal Patients Database
212 “LPD” (Italy), IQVIA-Disease Analyser “DA” (Germany), Clinical Practice Research
213 Datalink “CPRD” (UK), Integrated Primary Care Information “IPCI” (the Netherlands), two
214 had data for in-patients (Health Insurance Review & Assessment Service “HIRA” (South
215 Korea), Hospital del Mar “HMAR” (Spain)) and eight had both in and out-patient data
216 (IQVIA-OpenClaims, HEALTHVERITY, Information System for Research in Primary Care
217 “SIDIAP” (Spain [24]), Optum© de-identified Electronic health Record Dataset “OPTUM-
218 HER” (US), VA-OMOP, University of Colorado Anschutz Medical Campus Health Data

For peer review only - http://bmjopen.bmj.com/site/about/guidelines.xhtml

Compass “CUIMC” (US), CU-AMC-HDC, STAnford Medicine Research Data Repository “STARR-OMOP” (US [25])). A more detailed description of the included data sources is available in the Supporting Figure 1 and Table 1.

Study participants and follow-up

Two non-mutually exclusive cohorts were defined: 1) individuals *diagnosed* with COVID-19 (COVID-19 *diagnosed*) and 2) individuals *hospitalized* with COVID-19 (COVID-19 *hospitalized*). COVID-19 *diagnosed* cohort included individuals with a COVID-19 clinical diagnosis and/or a SARS-CoV-2 positive test. The COVID-19 *hospitalized* cohort included patients hospitalized with a COVID-19 clinical diagnosis or positive test 21 days before admission up to the end of their hospitalization. The codes used to identify COVID-19 cases are described in more detail in Supporting Table 2. The index date (i.e. cohort start date) was the date of COVID-19 diagnosis or positive test (whichever occurred first), for the diagnosed cohort; and the date of hospitalization, for the hospitalized cohort. Cohort participants were followed from the index date to the earliest of death, the end of the observation period, or 30 days after.

Baseline characteristics and outcomes of interest

The hypertension diagnosis, as well as the participants’ sex and age, were gathered at the index date and identified comorbidities in the year before the index date. Comorbidities (asthma, cancer, chronic kidney and liver disease, chronic obstructive pulmonary disease, dementia, heart disease, hyperlipidaemia, peripheral vascular disease, type 2 diabetes mellitus, obesity) were ascertained based on the Systematized Nomenclature of Medicine Current Terminology (SNOMED CT) hierarchy, with all descendant codes included. We selected and included comorbidities based on their prevalence in the cohorts of the participating sites and their clinical relevance to the COVID-19 research field [17-21]. Clinical epidemiologists generated a list of codes for the identification of prior medical conditions and

For peer review only

outcomes of interest using a web-based integrated platform (ATLAS tool: https://atlas.ohdsi.org/). The definition of the variables can be found in Supporting Table 3. Our main 30-day outcomes of interest were hospitalization and death for the COVID-19 *diagnosed* cohort, and requirement of intensive services (identified as any record of mechanical ventilation and/or a tracheostomy and/or extracorporeal membrane oxygenation procedure), acute respiratory distress syndrome (ARDS), arrhythmia, total cardiovascular events (ischemic stroke, haemorrhagic stroke, heart failure (heart failure during hospitalization for the hospitalized cohort), acute myocardial infarction or sudden cardiac death), sepsis, bleeding, venous thromboembolism (VTE) and death for the COVID-19 *hospitalized* cohort.

Statistical analyses

All data were standardized to the Observational Medical Outcomes Partnership (OMOP) Common Data Model (CDM) [26]. A common analytical code for the CHARYBDIS study was developed for the Observational Health Data Sciences and Informatics (OHDSI) Methods Library which was run locally in each database. Only aggregate results from each database were publicly shared. The CHARYBDIS protocol and source code can be found at https://github.com/ohdsi-studies/Covid19CharacterizationCharybdis. Demographics (sex and age categorized in 5-year age bands), comorbidities and 30-day incidence rates of outcomes were reported as proportions, along with 95% Confidence Intervals (CI). A minimum of 5 individuals was established to minimize the risk of identification of patients.

All results are reported by cohort, database and by hypertension status (with or without hypertension).

This is a descriptive study and no causal inference is intended. Multivariable regression or adjustment for confounding was therefore considered out of remit, and not included in our

For peer review only - http://bmjopen.bmj.com/site/about/guidelines.xhtml

269 study. We used R version 4.0.3 for data visualization. Before performing these analyses, all the data partners obtained Institutional Review Board (IRB) or equivalent governance approval. All data partners consented to the external sharing of the result set on data.ohdsi.org. Consent to participate was not required as only anonymised retrospective data was used for this study and no patient or GP contact was required.

274 **Patient and Public Involvement**

275 No patient involved

276 **RESULTS**

277 **Study population**

278 Overall, 2,851,035 patients diagnosed and 563,708 patients hospitalized with COVID-19 were identified in 15 databases from 8 countries (the US, South Korea, Germany, the Netherlands, France, Italy, Spain, and the UK). In total, 1,408,762 and 427,385 patients diagnosed and hospitalized with COVID-19, respectively, had a prior diagnosis of hypertension (Supporting Table 4). The prevalence of hypertension ranged from 17.4% to 48.3% in the COVID-19 *diagnosed* cohort, and from 25.6% to 85.9% in the COVID-19 *hospitalized cohort*.

285 **Baseline characteristics**

286 The age and sex distribution in the COVID-19 *diagnosed cohort* and in the COVID-19 *hospitalized cohort*, with and without hypertension are represented in Figures 1 and 2 respectively. Overall, in both cohorts, patients with hypertension were older than those without (higher proportion of patients aged above 50 across all databases). The proportion of patients *diagnosed* with COVID-19 and hypertension peaked at a younger age (55 to 70 years old) compared to those *hospitalized* (70 to 80 years old). The proportion of women with hypertension was greater in the *diagnosed* cohort (8.6 % to 55.6%) than in the *hospitalized* cohort (4.5% to 56%).

For peer review only - http://bmjopen.bmj.com/site/about/guidelines.xhtml

Baseline comorbidities

Figures 3 and 4 reports the proportion of baseline comorbidities of the COVID-19 *diagnosed* cohort (Figure 3) and COVID-19 *hospitalized* cohort (Figure 4), with and without hypertension. Patients with hypertension and COVID-19 *diagnosed* or *hospitalized* were frequently diagnosed with obesity, heart disease, dyslipidaemia, and type 2 diabetes, the proportion of which, more than double the ones found among patients with COVID-19 without hypertension.

30-day outcomes of interest

Thirty-day outcomes in people with and without hypertension in both the COVID-19 *diagnosed* and/or *hospitalized* cohorts are reported in Tables 1 and 2.

Patients with hypertension *diagnosed* with COVID-19 were more likely to be hospitalized (range 1.3% to 41.1% vs 1.4 to 15.9%) and had increased mortality (range 0.3% to 18.5% vs 0.2% to 11.8%) when compared to those without hypertension (Table 1).

For peer review only

Table 1. Comparison of 30-day outcomes of interest between COVID-19 patients with and without hypertension (HTN), in the COVID-19 diagnosed cohorts in the CHARYBDIS Network, % (95%CI)

Database	HTN	N	30-day outcomes	
			Death	Hospitalization
IQVIA-OpenClaims (US)	With	1,245,436	-	29.6 (29.5-29.7)
	Without	1,333,227	-	8.9 (8.9-8.9)
OPTUM-HER (US)	With	6,6432	1.7 (1.6-1.8)	26.4 (26.1-26.7)
	Without	11,1033	0.2 (0.2-0.2)	9.2 (9.0-9.4)

VA-OMOP (US)	With	34,093	5.4 (5.2-5.6)	23.4 (23.0-23.8)
	Without	21,464	0.7 (0.6-0.8)	6.1 (5.8-6.4)
HEALTHVERITY (US)	With	25,405	-	14.6 (14.2-15.0)
	Without	88,768	-	3.1 (3.0-3.2)
SIDIAP (Spain)	With	21,289	9.8 (9.4-10.2)	22.8 (22.2-23.4)
	Without	100,852	3.3 (3.2-3.4)	11.2 (11.0-11.4)
CUIMC (US)	With	3,672	11.8 (10.8-12.8)	41.1 (39.5-42.7)
	Without	4,847	2.0 (1.6-2.4)	15.9 (14.9-16.9)
CU-AMC-HDC (US)	With	2,461	5.9 (5.0-6.8)	35.8 (33.9-37.7)
	Without	4,809	0.7 (0.5-0.9)	11.2 (10.3-12.1)
IQVIA-DA (Germany)	With	2,418	0.3 (0.1-0.5)	-
	Without	5,553	-	-
STARR-OMOP (US)	With	1,246	0.6 (0.2-1.0)	24.6 (22.2-27.0)
	Without	2,082	-	14.0 (12.5-15.5)
CPRD (UK)	With	756	18.5 (15.7-21.3)	-
	Without	2,616	11.8 (10.6-13.0)	-
IPCI (The Netherlands)	With	676	13.6 (11.0-16.2)	1.3 (0.4-2.2)
	Without	2,371	3.1 (2.4-3.8)	1.4 (0.9-1.9)

For peer review only - http://bmjopen.bmj.com/site/about/guidelines.xhtml

Note: “-” means information is not available or <5 cases for all databases except for CU-AMC HDC where information is not available for <10 cases.

Patients with hypertension *hospitalized* with COVID-19 were more frequently diagnosed of ARDS (range 0.1 to 65.6% vs 0.1 to 54.7%), cardiac arrhythmia (range 0.5 to 45.8% vs 0.4 to 36.8%), and had increased mortality (range 1.8 to 25.1% vs 0.7 to 10.9%) as compared to those without hypertension (Table 2).

For peer review only

For peer review only - http://bmjopen.bmj.com/site/about/guidelines.xhtml

Table 2. Comparison of 30-days outcomes of interest between COVID-19 patients with and without hypertension (HTN), in the COVID-19 hospitalized cohorts in the CHARYBDIS Network, % (95%CI)

Database	HTN*	N	30-day outcomes						
			VTE†	Death	Cardiac arrhythmia	Sepsis	ARDS‡	Intensive services	Total CVE§
IQVIA-OpenClaims (US)	With	384,508	3.9 (3.8-4.0)	-	15.4 (15.3-15.5)	18.3 (18.2-18.4)	34.8 (34.6-35.0)	9.1 (9.0-9.2)	11.3 (11.2-11.4)
	Without	118,425	3.8 (3.7-3.9)	-	7.2 (7.1-7.3)	15.5 (15.3-15.7)	31.3 (31.0-31.6)	6.4 (6.3-6.5)	4.5 (4.4-4.6)
OPTUM-HER (US)	With	18,242	6.2 (5.9-6.5)	5.1 (4.8-5.4)	31.6 (30.9-32.3)	24.8 (24.2-25.4)	45.7 (45.0-46.4)	14.0 (13.5-14.5)	18.2 (17.6-18.8)
	Without	10,222	4.4 (4.0-4.8)	1.6 (1.4-1.8)	11.1 (10.5-11.7)	15.0 (14.3-15.7)	27.5 (26.6-28.4)	6.3 (5.8-6.8)	4.8 (4.4-5.2)
VA-OMOP (US)	With	8,996	7.3 (6.8-7.8)	15.4 (14.7-16.1)	33.9 (32.9-34.9)	20.0 (19.2-20.8)	43.9 (42.9-44.9)	17.1 (16.3-17.9)	21.0 (20.2-21.8)
	Without	1,475	6.9 (5.6-8.2)	7.6 (6.2-9.0)	16.8 (14.9-18.7)	16.2 (14.3-18.1)	39.6 (37.1-42.1)	11.2 (9.6-12.8)	7.3 (6.0-8.6)
HEALTHVERITY (US)	With	4,512	3.6 (3.1-4.1)	-	14.8 (13.8-15.8)	16.5 (15.4-17.6)	26.7 (25.4-28.0)	6.1 (5.4-6.8)	11.9 (11.0-12.8)
	Without	3,069	3.9 (3.2-4.6)	-	6.8 (5.9-7.7)	12.5 (11.3-13.7)	23.9 (22.4-25.4)	4.9 (4.1-5.7)	5.6 (4.8-6.4)

For peer review only

SIDIAP (Spain)	With	5,636	1.0 (0.7-1.3)	15.4 (14.5-16.3)	0.5 (0.3-0.7)	-	0.1 (0.0-0.2)	-	0.9 (0.7-1.1)
	Without	12,566	1.1 (0.9-1.3)	10.9 (10.4-11.4)	0.4 (0.3-0.5)	0.0 (0.0-0.0)	0.1 (0.0-0.2)	-	0.5 (0.4-0.6)
CUIMC (US)	With	1,708	3.9 (3.0-4.8)	25.1 (23.0-27.2)	12.1 (10.6-13.6)	6.1 (5.0-7.2)	16.0 (14.3-17.7)	2.2 (1.5-2.9)	8.1 (6.8-9.4)
	Without	892	3.6 (2.4-4.8)	10.2 (8.2-12.2)	4.7 (3.3-6.1)	5.3 (3.8-6.8)	17.8 (15.3-20.3)	1.8 (0.9-2.7)	3.8 (2.5-5.1)
CU-AMC HDC (US)	With	904	11.4 (9.3-13.5)	14.9 (12.6-17.2)	45.8 (42.6-49.0)	34.2 (31.1-37.3)	65.6 (62.5-68.7)	28.3 (25.4-31.2)	19.8 (17.2-22.4)
	Without	530	6.0 (4.0-8.0)	6.0 (4.0-8.0)	36.8 (32.7-40.9)	27.4 (23.6-31.2)	54.7 (50.5-58.9)	15.5 (12.4-18.6)	5.7 (3.7-7.7)
HIRA (South Korea)	With	1,943	0.7 (0.3-1.1)	7.7 (6.5-8.9)	4.4 (3.5-5.3)	5.3 (4.3-6.3)	2.6 (1.9-3.3)	4.9 (3.9-5.9)	10.0 (8.7-11.3)
	Without	5,656	NC	0.7 (0.5-0.9)	0.7 (0.5-0.9)	3.1 (2.6-3.6)	0.5 (0.3-0.7)	0.6 (0.4-0.8)	4.7 (4.1-5.3)
STARR-OMOP (US)	With	342	2.0 (0.5-3.5)	1.8 (0.4-3.2)	22.2 (17.8-26.6)	9.9 (6.7-13.1)	12.6 (9.1-16.1)	9.1 (6.1-12.1)	16.4 (12.5-20.3)
	Without	273	NC	-	6.6 (3.7-9.5)	7.0 (4.0-10.0)	11.4 (7.6-15.2)	5.5 (2.8-8.2)	-
HMAR (Spain)	With	594	3.2 (1.8-4.6)	14.3 (11.5-17.1)	23.1 (19.7-26.5)	1.9 (0.8-3.0)	12.6 (9.9-15.3)	13.5 (10.8-16.2)	12.1 (9.5-14.7)
	Without	1,417	2.6 (1.8-3.4)	3.9 (2.9-4.9)	6.6 (5.3-7.9)	0.7 (0.3-1.1)	7.3 (5.9-8.7)	6.6 (5.3-7.9)	2.2 (1.4-3.0)

Note: "-" means information is not available or <5 cases for all databases except for CU-AMC HDC where information is not available for <10 cases.

For peer review only - http://bmjopen.bmj.com/site/about/guidelines.xhtml

* hypertension; †: Venous thromboembolic (pulmonary embolism and deep vein thrombosis) events; ‡: Acute respiratory distress syndrome; §: cardiovascular disease events

For peer review only

For peer review only - http://bmjopen.bmj.com/site/about/guidelines.xhtml

DISCUSSION

This large multinational, multi-database cohort study, reports a greater prevalence of hypertension among patients *hospitalized* with COVID-19 compared to those *diagnosed* with COVID-19. Patients with hypertension diagnosed and/or hospitalized with COVID-19 were frequently diagnosed with obesity, heart disease, dyslipidaemia, and type 2 diabetes at baseline, compared to those without hypertension. They were also more likely to experience adverse outcomes including death and hospitalizations (in the COVID-19 *diagnosed cohort*) and cardiac arrhythmia, ARDS and death (in the COVID-19 *hospitalized* cohort) than patients without hypertension.

This is the first large multinational study that characterizes both in and out-patients with COVID-19, with and without prevalent hypertension. Hypertension was more prevalent in hospitalized patients compared to those diagnosed with COVID-19 (range from 25.6% to 85.9% vs 17.4 to 61.4% respectively). The observed variability between databases is similar to previous reports, where prevalence's ranged from 28.8% [7] to 60% [15]. However, these results should be put into context given that our highest rate (in both COVID-19 *diagnosed* and COVID-19 *hospitalized*) was observed in the VA-OMOP database from the US Department of Veterans Affairs (mostly men of older age).

As in the general population with hypertension [27], patients with hypertension diagnosed with COVID-19 in this study were more frequently diagnosed with heart disease or type 2 diabetes at baseline, than individuals without hypertension. These results are similar to what has been previously published, where patients with hypertension and COVID-19 also reported a higher prevalence of diabetes mellitus [6,8,12], cardiovascular diseases (other than hypertension) [8,12], and chronic kidney disease [8] compared to those without hypertension. This study further expands these previous findings identifying these same comorbidities in the out-patients

For peer review only - http://bmjopen.bmj.com/site/about/guidelines.xhtml

diagnosed with COVID-19 and adds obesity and dyslipidaemia to the list of conditions more frequently found among patients with COVID-19 and hypertension compared to those without hypertension. The higher prevalence of comorbid conditions found in this study among patients with hypertension *hospitalized* with COVID-19 compared to patients with hypertension *diagnosed* with COVID-19 suggests a poorer baseline health status.

Patients with hypertension *hospitalized* with COVID-19 were more likely to have worse disease progression with higher rates of ARDS (Prevalence per cent change (PC) between patients with and without hypertension ranging from -1.8% to 18.2%), more cardiac arrhythmia (PC ranging from 0.1% to 20.5%) and increased mortality (PC ranging from 3.5% to 14.9%). Previous studies have documented poorer clinical outcomes in patients with hypertension hospitalized with COVID-19 (including ARDS) [8, 12, 14, 19], the need for mechanical ventilation, admission to intensive care units [6, 13, 19] or an increased mortality [4,7,11-13, 19]. This study further showed that patients with hypertension *diagnosed* with COVID-19 were more likely to experience hospitalizations (PC between patients with and without hypertension ranging from -0.1% to 25.6%), and deaths (PC from 1.5% to 10.5%). These results highlight the importance of considering hypertension as a possible risk factor in the overall population *diagnosed* and not only in those *hospitalized* with COVID-19. It also adds to the current literature cardiac arrhythmia and cardiovascular diseases (other than hypertension) to the list of adverse outcomes more frequently diagnosed among patients with hypertension hospitalized with COVID-19 compared to those without hypertension.

This study has several strengths. This is the largest cohort study on individuals with hypertension who were *diagnosed* and/or *hospitalized* with COVID-19 to date. It provides novel insight into the characterization of patients *diagnosed* with COVID-19 and confers a greater external validity of its results compared to what has been published up to date (only *hospitalized* patients). It is also unique in its approach to characterizing COVID-19 cases

For peer review only

For peer review only - http://bmjopen.bmj.com/site/about/guidelines.xhtml

across an international network of healthcare databases, with diverse healthcare systems and policies, through a comprehensive federated approach, allowing the analysis of 15 databases without sharing patient identifiable data, hence respecting the patients' confidentiality at all times.

We recognize there are limitations to our approach. First, this study was intentionally descriptive and was deliberately not designed for causal inference. The observed differences between groups (eg. with versus without hypertension) should therefore not be interpreted as causal effects. Our patients were analysed depending on if they were *diagnosed* and/or *hospitalized* with COVID-19 according to database registration procedures, however, variations could have occurred during the processes by which patients were screened, tested, admitted, and registered across time and the databases. Additionally, the diagnosed and/or hospitalized cohorts were non-mutually exclusive, and therefore could be patients in the diagnosed cohort who were also hospitalized and vice versa.

This study was carried out using data recorded in routine clinical practice based on EHRs and/or claims, therefore, data could be incomplete or be erroneous, leading to potential misclassification. We have therefore selectively reported database-specific outcomes to minimize the impact of incompleteness. Differential reporting in databases is likely due to different coding practices as well as variability in disease severity, with milder/less symptomatic cases more likely being only diagnosed, and more severe ones hospitalized. Finally, the data that underpinned this study mostly came from the initial months of the COVID-19 pandemic and may not be representative of the COVID-19 cases diagnosed and/or hospitalized during subsequent periods.

CONCLUSIONS

COVID-19 patients with hypertension are more likely to have comorbidities, experience more severe outcomes including hospitalizations and deaths (among outpatients with

For peer review only

For peer review only - http://bmjopen.bmj.com/site/about/guidelines.xhtml

COVID-19) and experience more ARDS and deaths (among inpatients' with COVID-19) compared with patients without hypertension.

FIGURE LEGENDS

Figure 1. Comparison of the age and sex distribution in patients with a COVID-19 diagnosis with and without hypertension in the CHARYBDIS Network, %. Colour Red= with hypertension, Green= without hypertension.

Figure 2. Comparison of the age and sex distribution in patients with a COVID-19 hospitalization with and without hypertension in the CHARYBDIS Network, %. Colour Red=with hypertension, Colour Green=without hypertension.

Figure 3. **Comorbidities at baseline among patients with a COVID-19 diagnosis with and without hypertension in the CHARYBDIS Network, %.** Colour Red=with hypertension, Colour Green=without hypertension.

Figure 4. Comorbidities at baseline among patients with a COVID-19 hospitalization with and without hypertension in the CHARYBDIS Network, %. Colour Red=with hypertension, Colour Green=without hypertension.

CONTRIBUTOR'S STATEMENT

CR, AGS, CA, APU, AG, FN, AO, GH, PR, KK, TDS, KEL, SLD, MR, ER, SFB and AP provided substantial contributions to the conception or design, analysis, and interpretation of data for the work. CR, AGS, CA, APU, AG, FN, AO, GH, PR, KK, TDS, KEL, SLD, MR, ER, SFB, AP, DP, NV, GdeM, LSR, JPH, JMR, ILM, NHS, PRy, MAS, MM, CB, LYHL, TLA, W-U-RA, OA, HA, DD drafted or revised the manuscript critically for important intellectual content. All authors approved the final version of the manuscript and CR, AGS, CA, APU, AG, FN, AO, GH, PR, KK, TDS, LYHL, TLA, W-U-RA, OA, HA, DD, LMS,

For peer review only - http://bmjopen.bmj.com/site/about/guidelines.xhtml

CRei, JDP, SCY agreed to be accountable for all aspects of the work (KEL and SLD only for VA data) in ensuring that questions related to the accuracy or integrity of any part of the work are appropriately investigated and resolved.

ACKNOWLEDGEMENTS

We would like to acknowledge the patients who suffered from or died of this devastating disease, and their families and carers. We also thank the healthcare professionals involved in the management of COVID-19 during these challenging times, from primary care to intensive care units. The authors appreciate the Korean Health Insurance Review and Assessment Service for providing data.

We also thank the database curation teams around the world including the COVIDMAR Group (JPHorcajada, R.Güerri, J.Villar, M.Montero, S.Gómez-Zorrilla, M.Arenas-Miras, J.Gómez-Junyent, I.Arrieta, E.Sendra, S.Castañeda, E.Letang, I.Pelegrín, A.Rial, J.Rodríguez, C.Gimenez, J.Soldado, E.García).

We also thank the important contribution to this work of Dr Daniel Prieto-Alhambra.

DATA SHARING STATEMENT

Open Science is a guiding principle within OHDSI. As such, we provide unfettered access to all open-source analysis tools employed in this study via https://github.com/ohdsi-studies/Covid19CharacterizationCharybdis, as well as all data and results artefacts that do not include patient-level health information via https://data.odhsi.org/Covid19CharacterizationCharybdis/. Data partners contributing to this study remain custodians of their individual patient-level health information and hold either IRB exemption or approval for participation.

For peer review only

REFERENCES

1- Weekly Operational Update on COVID-19-6 Sep 2021 [internet]. WHO [cited 9th September 2021]. Available from https://www.who.int/publications/m/item/weekly-operational-update-on-covid-19---6-september-2021.

2- Unger T, Borghi C, Charchar F, Khan NA, Poulter NR, Prabhakaran D, et al. 2020 International Society of Hypertension global hypertension practice guidelines. J Hypertens. 2020;38:982-1004.

3-Cook TM. The importance of hypertension as a risk factor for severe illness and mortality in COVID-19. Anaesthesia. 2020;75:976-977.

4-Gupta S, Hayek SS, Wang W, Chan L, Mathews KS, Melamed ML, et al. Factors Associated With Death in Critically Ill Patients With Coronavirus Disease 2019 in the US. JAMA Intern Med. 2020;180:1–12.

5-Jiménez E, Fontán-Vela M, Valencia J, Fernandez-Jimenez I, Álvaro-Alonso EA, Izquierdo-García E, et al. Characteristics, complications and outcomes among 1549 patients hospitalised with COVID-19 in a secondary hospital in Madrid, Spain: a retrospective case series study. BMJ Open. 2020;10:e042398.

6-Huang S, Wang J, Liu F, Liu J, Cao G, Yang C, et al. COVID-19 patients with hypertension have more severe disease: a multicenter retrospective observational study. Hypertens Res. 2020;43:824-831.

7-Park BE, Lee JH, Park HK, Kim HN, Jang SY, Bae MH, et al. Impact of Cardiovascular Risk Factors and Cardiovascular Diseases on Outcomes in Patients Hospitalized with COVID-19 in Daegu Metropolitan City. J Korean Med Sci. 2021;36:e15.

For peer review only

8- Yao Q, Ni J, Hu TT, Cai ZL, Zhao JH, Xie QW, et al. Clinical characteristics and outcomes in coronavirus disease 2019 (COVID-19) patients with and without hypertension: a retrospective study. Rev Cardiovasc Med. 2020;21:615-625.

9- Zhou F, Yu T, Du R, Fan G, Liu Y, Liu Z, et al. Clinical course and risk factors for mortality of adult inpatients with COVID-19 in Wuhan, China: a retrospective cohort study. Lancet. 2020;395(10229):1054-1062.

10-Wang Y, Lu X, Li Y, Chen H, Chen T, Su N, et al. Clinical Course and Outcomes of 344 Intensive Care Patients with COVID-19. Am J Respir Crit Care Med. 2020;201:1430-1434.

11-Grasselli G, Zangrillo A, Zanella A, Antonelli M, Cabrini L, Castelli A, et al. Baseline Characteristics and Outcomes of 1591 Patients Infected With SARS-CoV-2 Admitted to ICUs of the Lombardy Region, Italy. JAMA. 2020;323:1574-1581.

12-Gao C, Cai Y, Zhang K, Zhou L, Zhang Y, Zhang X, et al. Association of hypertension and antihypertensive treatment with COVID-19 mortality: a retrospective observational study. Eur Heart J. 2020;41:2058-2066.

13- Guan WJ, Liang WH, Zhao Y, Liang HR, Chen ZS, Li YM, et al. Comorbidity and its impact on 1590 patients with COVID-19 in China: a nationwide analysis. Eur Respir J. 2020;55:2000547.

14- Ji W, Huh K, Kang M, Hong J, Bae GH, Lee R, et al. Effect of Underlying Comorbidities on the Infection and Severity of COVID-19 in Korea: a Nationwide Case-Control Study. J Korean Med Sci. 2020;35:e237.

15- Chilimuri S, Sun H, Alemam A, Mantri N, Shehi E, Tejada J, et al. Predictors of Mortality in Adults Admitted with COVID-19: Retrospective Cohort Study from New York City. West J Emerg Med. 2020;21:779-784.

For peer review only

For peer review only - http://bmjopen.bmj.com/site/about/guidelines.xhtml

16- Richardson S, Hirsch JS, Narasimhan M, Crawford JM, McGinn T, Davidson KW, et al. Presenting Characteristics, Comorbidities, and Outcomes Among 5700 Patients Hospitalized With COVID-19 in the New York City Area. JAMA. 2020;323:2052-2059. Erratum in: JAMA. 2020;323:2098.

17-Dorjee K, Kim H, Bonomo E, Dolma R. Prevalence and predictors of death and severe disease in patients hospitalized due to COVID-19: A comprehensive systematic review and meta-analysis of 77 studies and 38,000 patients. PLoS One. 2020;15:e0243191.

18-Javanmardi F, Keshavarzi A, Akbari A, Emami A, Pirbonyeh N. Prevalence of underlying diseases in dead cases of COVID-19: A systematic review and meta-analysis. PLoS One. 2020;15:e0241265.

19-Pranata R, Lim MA, Huang I, Raharjo SB, Lukito AA. Hypertension is associated with increased mortality and severity of disease in COVID-19 pneumonia: A systematic review, meta-analysis and meta-regression. J Renin Angiotensin Aldosterone Syst. 2020;21:1470320320926899.

20- Silverio A, Di Maio M, Citro R, Esposito L, Iuliano G, Bellino M, et al. Cardiovascular risk factors and mortality in hospitalized patients with COVID-19: systematic review and meta-analysis of 45 studies and 18,300 patients. BMC Cardiovasc Disord. 2021;21:23.

21- Moazzami B, Chaichian S, Kasaeian A, Djalalinia S, Akhlaghdoust M, Eslami M, et al. Metabolic risk factors and risk of Covid-19: A systematic review and meta-analysis. PLoS One. 2020;15:e0243600.

22- Baj J, Karakuła-Juchnowicz H, Teresiński G, Buszewicz G, Ciesielka M, Sitarz E, et al. COVID-19: Specific and Non-Specific Clinical Manifestations and Symptoms: The Current State of Knowledge. J Clin Med. 2020;9:1753.

For peer review only - http://bmjopen.bmj.com/site/about/guidelines.xhtml

23- Prieto-Alhambra D, Kostka K, Duarte-Salles T, Prats-Uribe A, Sena A, Pistillo A, et al. Unraveling COVID-19: a large-scale characterization of 4.5 million COVID-19 cases using CHARYBDIS. Res Sq [Preprint]. 2021:rs.3.rs-279400.

24- García-Gil M del M, Hermosilla E, Prieto-Alhambra D, Fina F, Rosell M, Ramos R, et al. Construction and validation of a scoring system for the selection of high-quality data in a Spanish population primary care database (SIDIAP). Inform Prim Care. 2011; 19(3): 135–45

25-Datta S, Posada J, Olson G, Li W, O'Reilly C, Balraj D, et al. A new paradigm for accelerating clinical data science at Stanford Medicine. arXiv:2003.10534

26-Voss EA, Makadia R, Matcho A, Ma Q, Knoll C, Schuemie M, et al. Feasibility and utility of applications of the common data model to multiple, disparate observational health databases. J Am Med Inform Assoc. 2015;22:553-64.

27-Tsimihodimos V, Gonzalez-Villalpando C, Meigs JB, Ferrannini E. Hypertension and Diabetes Mellitus: Coprediction and Time Trajectories. Hypertension. 2018;71:422-428.

For peer review only

For peer review only - http://bmjopen.bmj.com/site/about/guidelines.xhtml

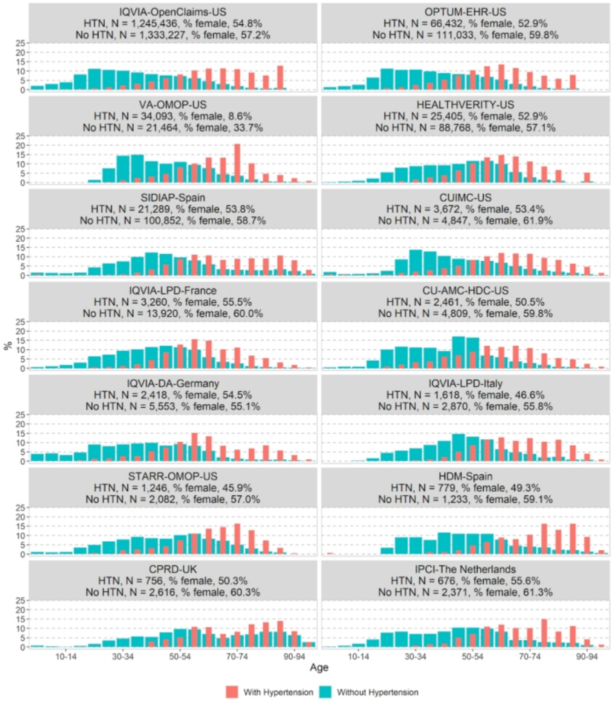

Comparison of the age and sex distribution in patients with a COVID-19 diagnosis with and without hypertension in the CHARYBDIS Network, %. Colour Red= with hypertension, Green= without hypertension.

531x752mm (79 x 79 DPI)

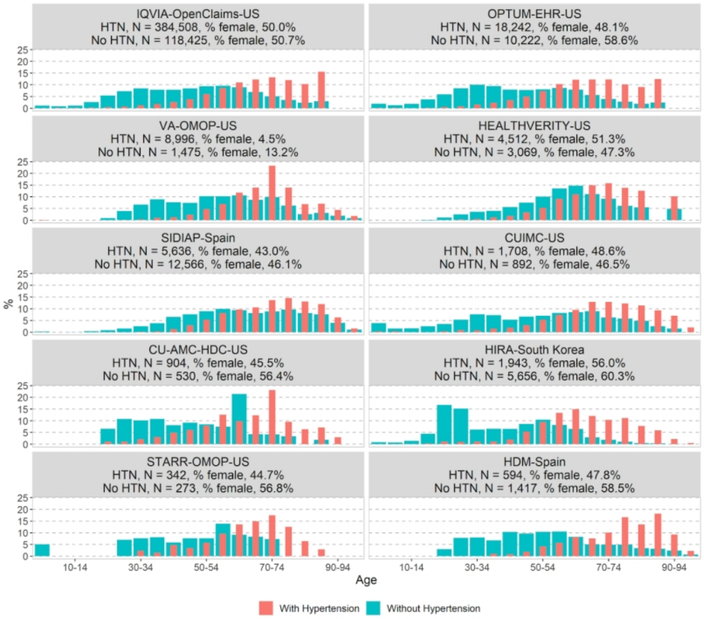

Comparison of the age and sex distribution in patients with a COVID-19 hospitalization with and without hypertension in the CHARYBDIS Network, %. Colour Red=with hypertension, Colour Green=without hypertension.

532x752mm (79 x 79 DPI)

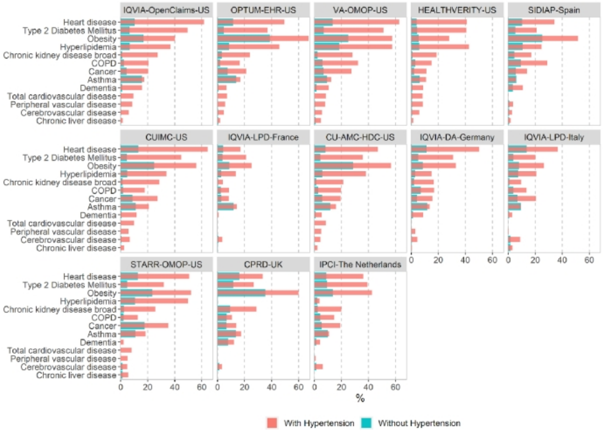

Comorbidities at baseline among patients with a COVID-19 diagnosis with and without hypertension in the CHARYBDIS Network, %. Colour Red=with hypertension, Colour Green=without hypertension.

210x297mm (300 x 300 DPI)

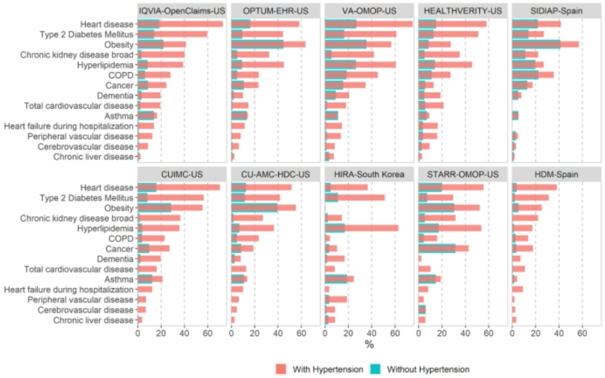

Comorbidities at baseline among patients with a COVID-19 hospitalization with and without hypertension in the CHARYBDIS Network, %. Colour Red=with hypertension, Colour Green=without hypertension.

532x752mm (79 x 79 DPI)

Supporting Figure 1. Flowchart showing the selection of databases included in the analyses

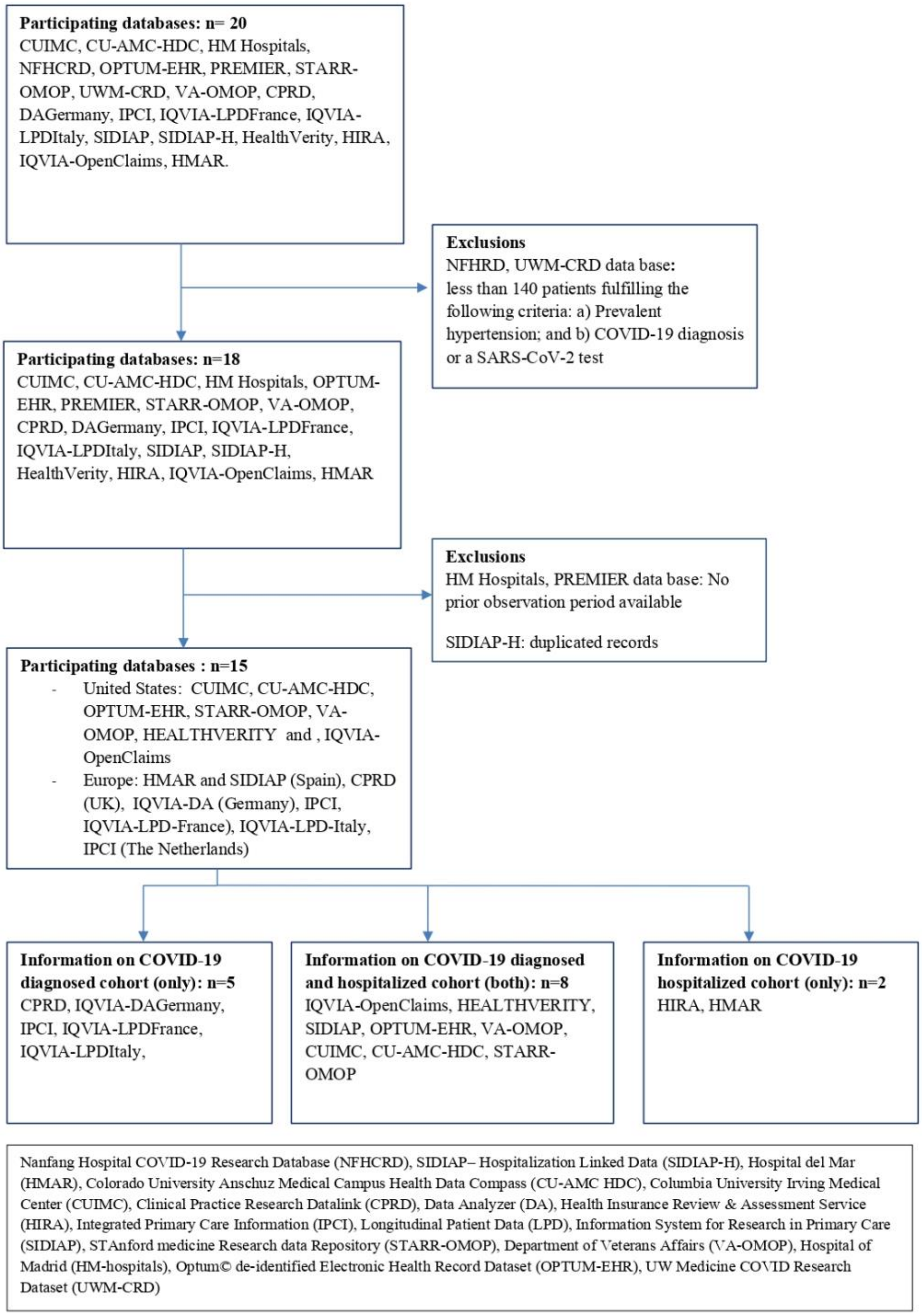

Supporting Table 1. Description of included databases

Institution Name/ Database	Database Description	Country
Janssen Research & Development<br>The Clinical Practice Research Datalink (CPRD)	The Clinical Practice Research Datalink (CPRD) is a governmental not-for-profit research service jointly funded by the NHS National Institute for Health Research (NIHR) and the Medicines and Healthcare products Regulatory Agency (MHRA) a part of the Department of Health United Kingdom (UK). CPRD consists of data collected from UK primary care for all ages. This includes conditions observations measurements and procedures that the general practitioner is made aware of in addition to any prescriptions as prescribed by the general practitioner. In addition to primary care there are also linked secondary care records for a small number of people. The major data elements contained within this database are outpatient prescriptions given by the general practitioner (coded with Multilex codes) and outpatient clinical referral immunization or test events that the general practitioner knows about (coded in Read or ICD10 or LOINC codes). The database also contains the patients' year of births and any date of deaths.	United Kingdom
IDIAPJGol<br>The Information System for Research in Primary Care (SIDIAP)	The Information System for Research in Primary Care (SIDIAP; www.sidiap.org) is a primary care records database that covers approximately 80% of the population of Catalonia North-East Spain. Healthcare is universal and tax-payer funded in the region and primary care physicians are gatekeepers for all care and responsible for repeat prescriptions.	Spain
Stanford Medicine Research Data Repository (STARR-OMOP)	A clinical data warehouse containing live Epic data from Stanford Health Care the Stanford Children's Hospital	United States

For peer review only

	the University Healthcare Alliance and Packard Children's Health Alliance clinics.<br>Reference: Datta S Posada J Olson G *et al.* A new paradigm for accelerating clinical data science at Stanford Medicine. *arXiv* 2020; published online March 17. http://arxiv.org/abs/2003.10534 (accessed Aug 20 2020).	
Columbia University Irving Medical Center (CUIMC)	The clinical data warehouse of New York-Presbyterian Hospital/Columbia University Irving Medical Center New York NY based on its current and previous electronic health record systems with data spanning over 30 years and including over 6 million patients	United States
IQVIA<br>Open Claims	Pre-adjudicated claims covering over 300 Million lives (~80% of the US) collected from office-based physicians and specialists via office management software and clearinghouse switch sources for the purpose of reimbursement.	United States
HIRA<br>Health Insurance Review & Assessment Service	National claim data from a single insurance service from South Korea, It contains the observational medical records (including both inpatient and outpatient) of a patient while they are qualified to get the national medical insurance.	South Korea
HMAR<br>Hospital del mar	Anonymized data from the Electronic Medical Records from Hospital del Mar (Barcelona, Spain). Hospital belonging to the Spanish National Health System (public), attending the Eastern area of Barcelona City. Includes hospital data collected routinely in the clinical practice, both structured and unstructured information, extracted using a free text analysis tool (with natural language processing): Inpatient (hospital) care, Outpatient specialist care, Emergency Room Visits and partial information from other settings like primary care and pharmacy care present in free text notes from EMRs. All subjects with at least one healthcare encounter with the Hospital within approximately last 20 years are included (approximately 0.6 M subjects, with more than	Spain

For peer review only

	5 M hospitalizations/visits). Hospital del Mar data are made available through collaboration with TFS / IOMED.	
OPTUM-EHR<br>Optum® de-identified Electronic Health Record Dataset	Optum ® de-identified Electronic Health Record Dataset is derived from dozens of healthcare provider organizations in the United States (that include more than 700 hospitals and 7,000 Clinics treating more than 103 million patients) receiving care in the United States. The medical record data includes clinical information, inclusive of prescriptions as prescribed and administered, lab results, vital signs, body measurements, diagnoses, procedures, and information derived from clinical Notes using Natural Language Processing (NLP)	United States
IPCI<br>Integrated Primary Care Information	The Integrated Primary Care Information (IPCI) database is collected from EHR records of patients registered with 391 GPs throughout the Netherlands. The database contains records from approximately 2.6 million patients out of a Dutch population of 17M (8.2%) starting in 1996.	The Netherlands
DA Germany<br>IQVIA Disease Analyser Germany	IQVIA DA Germany is collected from extracts of patient management software used by GPs and specialists practicing in ambulatory care settings. Data coverage includes more than 34M distinct person records out of at total population of 80M (42.5%) in the country and collected from 2,734 providers. Dates of service include from 1992 through March 2020	Germany
LPD-Italy<br>IQVIA LPD Italy	LPD Italy is comprised of anonymised patient records collected from software used by GPs during an office visit to document patients' clinical records. Data coverage includes over 2M patient records with at least one visit and 119.5M prescription orders across 900 GP practices. Dates of service include from 2004 through	Italy

For peer review only

	present. Observation time is defined by the first and last consultation dates. Drugs are captured as prescription records with product, quantity, dosing directions, strength, indication and date of consultation.	
LPD-France	LPD France is a computerised network of physicians including GPs who contribute to a centralised database of anonymised patient EMR. Currently, >1200 GPs from 400 practices are contributing to the database covering 7.8M patients in France. The database covers a time period from 1994 through the present. Observation time is defined by the first and last consultation dates. Drug information is derived from GP prescriptions. Drugs obtained over the counter by the patient outside the prescription system are not reported	France
HEALTHVERITY	This HealthVerity derived data set contains de-identified patient information with an antibody and/or diagnostic test for COVID-19 linked to all available Medical Claims and Pharmacy Data from select private data providers participating in the HealthVerity marketplace.	United States
University of Colorado Anschuz Medical Campus Health Data Compass (CU-AMC HDC)	Health Data Compass (HDC) is a multi-institutional data warehouse. HDC contains inpatient and outpatient electronic medical data including patient, encounter, diagnosis, procedures, medications, laboratory results from two electronic medical record systems (UCHealth and Children's Hospital of Colorado), state-level all-payers claims data, and the Colorado death registry. Acknowledgement statement: Supported by the Health Data Compass Data Warehouse project (healthdatacompass.org)	
Department of Veterans Affairs VA- OMOP	VA-OMOP data reflects the national Department of Veterans Affairs health care system which is the largest integrated provider of medical and mental health services	United States

	in the United States. Care is provided at 170 VA Medical Centers and 1 063 outpatient sites serving more than 9 million enrolled Veterans each year.	

Supporting Table 2. Definitions and codes used to identify COVID-19 cases

The below tables summarises the concepts used to identify patients diagnosed with COVID-19 . The full description of the logic used to identify patients diagnosed and hospitalized is provided at https://atlas.ohdsi.org/#/cohortdefinition/200 and https://atlas.ohdsi.org/#/cohortdefinition/197 respectively.

**COVID-19 condition codes**		
**Id**	**Name**	**Vocabulary**
756023	Acute bronchitis due to COVID-19	OMOP Extension
756044	Acute respiratory distress syndrome (ARDS) due to COVID-19	OMOP Extension
756061	Asymptomatic COVID-19	OMOP Extension
756031	Bronchitis due to COVID-19	OMOP Extension
439676	Coronavirus infection	SNOMED
37311061	Disease caused by 2019-nCoV	SNOMED
4100065	Disease due to Coronaviridae	SNOMED
37310284	Encephalopathy caused by 2019 novel coronavirus	SNOMED

For peer review only

37310283	Gastroenteritis caused by 2019 novel coronavirus	SNOMED
4248811	Healthcare associated severe acute respiratory syndrome	SNOMED
756081	Infection of lower respiratory tract due to COVID-19	OMOP Extension
37310286	Infection of upper respiratory tract caused by 2019 novel coronavirus	SNOMED
45763594	Middle East respiratory syndrome	SNOMED
37310287	Myocarditis caused by 2019 novel coronavirus	SNOMED
37310254	Otitis media caused by 2019 novel coronavirus	SNOMED
37310285	Pneumonia caused by 2019 novel coronavirus	SNOMED
37016927	Pneumonia caused by Human coronavirus	SNOMED
40479642	Pneumonia due to Severe acute respiratory syndrome coronavirus	SNOMED
756039	Respiratory infection due to COVID-19	OMOP Extension
320651	Severe acute respiratory syndrome	SNOMED
37396171	Severe acute respiratory syndrome of upper respiratory tract	SNOMED
37311060	Suspected disease caused by 2019-nCoV	SNOMED
**COVID-19 specific testing - Positive**		
37310282	2019 novel coronavirus detected	SNOMED
**COVID-19 specific testing (note these required a corresponding value as concept of: Detected Positive or Present)**		

For peer review only

37310255	Detection of 2019 novel coronavirus using polymerase chain reaction technique	SNOMED
700360	Infectious agent detection by nucleic acid (DNA or RNA); severe acute respiratory syndrome coronavirus 2 (SARS-CoV-2) (Coronavirus disease [COVID-19]) amplified probe technique	CPT4
37310258	Measurement of 2019 novel coronavirus antibody	SNOMED
37310257	Measurement of 2019 novel coronavirus antigen	SNOMED
756055	Measurement of Severe acute respiratory syndrome coronavirus 2 (SARS-CoV-2)	OMOP Extension
586310	Measurement of Severe acute respiratory syndrome coronavirus 2 (SARS-CoV-2) Genetic material using Molecular method	OMOP Extension
704991	Measurement of Severe acute respiratory syndrome coronavirus 2 (SARS-CoV-2) in Blood	OMOP Extension
756029	Measurement of Severe acute respiratory syndrome coronavirus 2 (SARS-CoV-2) in Respiratory specimen	OMOP Extension
586307	Measurement of Severe acute respiratory syndrome coronavirus 2 (SARS-CoV-2) in Saliva	OMOP Extension
705107	Measurement of Severe acute respiratory syndrome coronavirus 2 (SARS-CoV-2) in Sample from nose	OMOP Extension
586309	Measurement of Severe acute respiratory syndrome coronavirus 2 (SARS-CoV-2) in Specified specimen	OMOP Extension
756065	Measurement of Severe acute respiratory syndrome coronavirus 2 (SARS-CoV-2) in Unspecified specimen	OMOP Extension
704992	Measurement of Severe acute respiratory syndrome coronavirus 2 (SARS-CoV-2) using Culture method	OMOP Extension
705001	Measurement of Severe acute respiratory syndrome coronavirus 2 (SARS-CoV-2) using Nucleic acid amplification technique	OMOP Extension
705000	Measurement of Severe acute respiratory syndrome coronavirus 2 (SARS-CoV-2) using Nucleic acid amplification technique in Blood	OMOP Extension
756085	Measurement of Severe acute respiratory syndrome coronavirus 2 (SARS-CoV-2) using Nucleic acid amplification technique in Respiratory specimen	OMOP Extension
586308	Measurement of Severe acute respiratory syndrome coronavirus 2 (SARS-CoV-2) using Nucleic acid amplification technique in Saliva	OMOP Extension
705106	Measurement of Severe acute respiratory syndrome coronavirus 2 (SARS-CoV-2) using Nucleic acid amplification technique in Sample from nose	OMOP Extension
756084	Measurement of Severe acute respiratory syndrome coronavirus 2 (SARS-CoV-2) using Nucleic acid amplification technique in Unspecified specimen	OMOP Extension
704993	Measurement of Severe acute respiratory syndrome coronavirus 2 (SARS-CoV-2) using Sequencing	OMOP Extension

For peer review only

586516	SARS-CoV-2 (COVID19) [Presence] in Unspecified specimen by Organism specific culture	LOINC
723480	SARS-CoV-2 (COVID19) Ab [Interpretation] in Serum or Plasma	LOINC
586515	SARS-CoV-2 (COVID19) Ab [Presence] in Serum or Plasma by Immunoassay	LOINC
586522	SARS-CoV-2 (COVID19) Ab [Units/volume] in Serum or Plasma by Immunoassay	LOINC
706179	SARS-CoV-2 (COVID19) Ab panel - Serum or Plasma by Immunoassay	LOINC
723477	SARS-CoV-2 (COVID19) Ag [Presence] in Respiratory specimen by Rapid immunoassay	LOINC
706166	SARS-CoV-2 (COVID19) E gene [Cycle Threshold #] in Unspecified specimen by NAA with probe detection	LOINC
586523	SARS-CoV-2 (COVID19) E gene [Presence] in Respiratory specimen by NAA with probe detection	LOINC
586518	SARS-CoV-2 (COVID19) E gene [Presence] in Serum or Plasma by NAA with probe detection	LOINC
706174	SARS-CoV-2 (COVID19) E gene [Presence] in Unspecified specimen by NAA with probe detection	LOINC
723473	SARS-CoV-2 (COVID19) IgA Ab [Presence] in Serum or Plasma by Immunoassay	LOINC
586521	SARS-CoV-2 (COVID19) IgA Ab [Presence] in Serum Plasma or Blood by Rapid immunoassay	LOINC
723459	SARS-CoV-2 (COVID19) IgA Ab [Units/volume] in Serum or Plasma by Immunoassay	LOINC
757686	SARS-CoV-2 (COVID19) IgA+IgM [Presence] in Serum or Plasma by Immunoassay	LOINC
586527	SARS-CoV-2 (COVID19) IgG Ab [Presence] in DBS by Immunoassay	LOINC
723474	SARS-CoV-2 (COVID19) IgG Ab [Presence] in Serum or Plasma by Immunoassay	LOINC
706181	SARS-CoV-2 (COVID19) IgG Ab [Presence] in Serum Plasma or Blood by Rapid immunoassay	LOINC
706177	SARS-CoV-2 (COVID19) IgG Ab [Units/volume] in Serum or Plasma by Immunoassay	LOINC
706176	SARS-CoV-2 (COVID19) IgG and IgM panel - Serum Plasma or Blood by Rapid immunoassay	LOINC
723479	SARS-CoV-2 (COVID19) IgG+IgM Ab [Presence] in Serum or Plasma by Immunoassay	LOINC

For peer review only

723475	SARS-CoV-2 (COVID19) IgM Ab [Presence] in Serum or Plasma by Immunoassay	LOINC
706180	SARS-CoV-2 (COVID19) IgM Ab [Presence] in Serum Plasma or Blood by Rapid immunoassay	LOINC
706178	SARS-CoV-2 (COVID19) IgM Ab [Units/volume] in Serum or Plasma by Immunoassay	LOINC
706167	SARS-CoV-2 (COVID19) N gene [Cycle Threshold #] in Unspecified specimen by NAA with probe detection	LOINC
706157	SARS-CoV-2 (COVID19) N gene [Cycle Threshold #] in Unspecified specimen by Nucleic acid amplification using CDC primer-probe set N1	LOINC
706155	SARS-CoV-2 (COVID19) N gene [Cycle Threshold #] in Unspecified specimen by Nucleic acid amplification using CDC primer-probe set N2	LOINC
715272	SARS-CoV-2 (COVID19) N gene [Presence] in Nasopharynx by NAA with probe detection	LOINC
757678	SARS-CoV-2 (COVID19) N gene [Presence] in Nose by NAA with probe detection	LOINC
706161	SARS-CoV-2 (COVID19) N gene [Presence] in Respiratory specimen by NAA with probe detection	LOINC
586524	SARS-CoV-2 (COVID19) N gene [Presence] in Respiratory specimen by Nucleic acid amplification using CDC primer-probe set N1	LOINC
586525	SARS-CoV-2 (COVID19) N gene [Presence] in Respiratory specimen by Nucleic acid amplification using CDC primer-probe set N2	LOINC
586520	SARS-CoV-2 (COVID19) N gene [Presence] in Serum or Plasma by NAA with probe detection	LOINC
706175	SARS-CoV-2 (COVID19) N gene [Presence] in Unspecified specimen by NAA with probe detection	LOINC
706156	SARS-CoV-2 (COVID19) N gene [Presence] in Unspecified specimen by Nucleic acid amplification using CDC primer-probe set N1	LOINC
706154	SARS-CoV-2 (COVID19) N gene [Presence] in Unspecified specimen by Nucleic acid amplification using CDC primer-probe set N2	LOINC
757680	SARS-CoV-2 (COVID19) neutralizing antibody [Presence] in Serum by pVNT	LOINC
757679	SARS-CoV-2 (COVID19) neutralizing antibody [Titer] in Serum by pVNT	LOINC
723469	SARS-CoV-2 (COVID19) ORF1ab region [Cycle Threshold #] in Respiratory specimen by NAA with probe detection	LOINC
706168	SARS-CoV-2 (COVID19) ORF1ab region [Cycle Threshold #] in Unspecified specimen by NAA with probe detection	LOINC
723478	SARS-CoV-2 (COVID19) ORF1ab region [Presence] in Respiratory specimen by NAA with probe detection	LOINC

For peer review only

723464	SARS-CoV-2 (COVID19) ORF1ab region [Presence] in Unspecified specimen by NAA with probe detection	LOINC
723471	SARS-CoV-2 (COVID19) RdRp gene [Cycle Threshold #] in Respiratory specimen by NAA with probe detection	LOINC
723470	SARS-CoV-2 (COVID19) RdRp gene [Cycle Threshold #] in Unspecified specimen by NAA with probe detection	LOINC
706160	SARS-CoV-2 (COVID19) RdRp gene [Presence] in Respiratory specimen by NAA with probe detection	LOINC
706173	SARS-CoV-2 (COVID19) RdRp gene [Presence] in Unspecified specimen by NAA with probe detection	LOINC
586528	SARS-CoV-2 (COVID19) RNA [Cycle Threshold #] in Respiratory specimen by NAA with probe detection	LOINC
586529	SARS-CoV-2 (COVID19) RNA [Cycle Threshold #] in Unspecified specimen by NAA with probe detection	LOINC
715262	SARS-CoV-2 (COVID19) RNA [Log #/volume] (viral load) in Unspecified specimen by NAA with probe detection	LOINC
723476	SARS-CoV-2 (COVID19) RNA [Presence] in Nasopharynx by NAA with non-probe detection	LOINC
586526	SARS-CoV-2 (COVID19) RNA [Presence] in Nasopharynx by NAA with probe detection	LOINC
757677	SARS-CoV-2 (COVID19) RNA [Presence] in Nose by NAA with probe detection	LOINC
706163	SARS-CoV-2 (COVID19) RNA [Presence] in Respiratory specimen by NAA with probe detection	LOINC
715260	SARS-CoV-2 (COVID19) RNA [Presence] in Saliva (oral fluid) by NAA with probe detection	LOINC
715261	SARS-CoV-2 (COVID19) RNA [Presence] in Saliva (oral fluid) by Sequencing	LOINC
723463	SARS-CoV-2 (COVID19) RNA [Presence] in Serum or Plasma by NAA with probe detection	LOINC
706170	SARS-CoV-2 (COVID19) RNA [Presence] in Unspecified specimen by NAA with probe detection	LOINC
706158	SARS-CoV-2 (COVID19) RNA panel - Respiratory specimen by NAA with probe detection	LOINC
706169	SARS-CoV-2 (COVID19) RNA panel - Unspecified specimen by NAA with probe detection	LOINC
723467	SARS-CoV-2 (COVID19) S gene [Cycle Threshold #] in Respiratory specimen by NAA with probe detection	LOINC
723468	SARS-CoV-2 (COVID19) S gene [Cycle Threshold #] in Unspecified specimen by NAA with probe detection	LOINC

For peer review only

723465	SARS-CoV-2 (COVID19) S gene [Presence] in Respiratory specimen by NAA with probe detection	LOINC
586519	SARS-CoV-2 (COVID19) S gene [Presence] in Serum or Plasma by NAA with probe detection	LOINC
723466	SARS-CoV-2 (COVID19) S gene [Presence] in Unspecified specimen by NAA with probe detection	LOINC
586517	SARS-CoV-2 (COVID19) whole genome [Nucleotide sequence] in Isolate by Sequencing	LOINC
40218805	Testing for SARS-CoV-2 in CDC laboratory	HCPCS
40218804	Testing for SARS-CoV-2 in non-CDC laboratory	HCPCS

Supporting Table 3. Definitions and codes used for hypertension and other comorbidities

**Name**	**Included Codes**
Hyperlipidemia	https://atlas.ohdsi.org/#/concept/432867
Chronic kidney disease	https://atlas.ohdsi.org/#/cohortdefinition/312
Cancer	https://atlas.ohdsi.org/#/cohortdefinition/222
Asthma	https://atlas.ohdsi.org/#/cohortdefinition/218
Dementia	https://atlas.ohdsi.org/#/cohortdefinition/226
Total cardiovascular disease	https://atlas.ohdsi.org/#/cohortdefinition/246
Peripheral vascular disease	https://atlas.ohdsi.org/#/concept/321052

For peer review only

Cerebrovascular disease	https://atlas.ohdsi.org/#/concept/381591
Chronic liver disease	https://atlas.ohdsi.org/#/concept/4212540
Chronic obstructive pulmonary disease	https://atlas.ohdsi.org/#/cohortdefinition/219
Heart disease	https://atlas.ohdsi.org/#/cohortdefinition/231
Hypertension	https://atlas.ohdsi.org/#/cohortdefinition/227
Obesity	https://atlas.ohdsi.org/#/cohortdefinition/224
Type 2 Diabetes Mellitus	https://atlas.ohdsi.org/#/cohortdefinition/311

For peer review only

Supporting Table 4. Prevalence of hypertension among COVID-19 patients in the diagnosed and hospitalised cohorts in the CHARYBDIS Network.

	**Diagnosed with COVID-19**		**Hospitalized with COVID-19**	
	**N of prevalent cases**	**% (95%CI)**	**N of prevalent cases**	**% (95%CI)**
IQVIA-OpenClaims-US	1,245,436	48.3 (48.2-48.4)	384,508	76.5 (76.3-76.6)
OPTUM-EHR-US	66,432	37.4 (37.2-37.7)	18,242	64.1 (63.5-64.6)
VA-OMOP-US	34,093	61.4 (61.0-61.8)	8,996	85.9 (85.2-86.6)

HEALTHVERITY-US	25,405	22.3 (22.0-22.5)	4,512	59.5 (58.4-60.6)
SIDIAP-Spain	21,289	17.4 (17.2-17.6)	5,636	31.0 (30.3-31.6)
CUIMC-US	3,672	43.1 (42.1-44.2)	1,708	65.7 (63.9-67.5)
IQVIA-LPD-France	3,260	19.0 (18.4-19.6)	-	-
CU-AMC-HDC-US	2,461	33.9 (32.8-34.9)	904	63.0 (60.5-65.5)
IQVIA-DA-Germany	2,418	30.3 (29.3-31.3)	-	-
HIRA-South Korea	-	-	1943	25.6 (24.6-26.6)
IQVIA-LPD-Italy	1,618	36.1 (34.6-37.5)	-	-
STARR-OMOP-US	1,246	37.4 (35.8-39.1)	342	55.6 (51.7-59.5)
HMAR-Spain	-	-	594	29.5 (27.5-31.5)
CPRD-UK	756	22.4 (21.0-23.8)	-	-
IPCI-The Netherlands	676	22.2 (20.7-23.7)	-	-

For peer review only

BMJ Open

Characteristics and outcomes of COVID-19 patients with and without prevalent hypertension: a multinational cohort study

Journal:	*BMJ Open*
Manuscript ID	bmjopen-2021-057632.R1
Article Type:	Original research
Date Submitted by the Author:	08-Nov-2021
Complete List of Authors:	Reyes, Carlen; GREMPAL Research Group, Fundació Institut Universitari per a la recerca a l'Atenció Primària de Salut Jordi Gol i Gurina (IDIAPJGol), and CIBERFes, Universitat Autonoma de Barcelona and Instituto de Salud Carlos III,<br>Pistillo, Andrea ; Fundació Institut Universitari per a la recerca a l'Atenció Primària de Salut Jordi Gol i Gurina (IDIAPJGol),<br>Fernández-Bertolín, Sergio; Fundació Institut Universitari per a la recerca a l'Atenció Primària de Salut Jordi Gol i Gurina (IDIAPJGol),<br>Recalde, Martina; IDIAP Jordi Gol<br>Roel, Elena; IDIAP Jordi Gol<br>Puente, Diana; IDIAP Jordi Gol, Research<br>Sena, Anthony; Janssen Research and Development Titusville<br>Blacketer, Clair; Janssen Research and Development Titusville<br>Lai, Lana; The University of Manchester, School of Medical Sciences<br>Alshammari, Thamir; King Saud University, Medication Safety Research Chair; Saudi Food and Drug Authority,<br>Ahmed, Waheed-UI-Rahman; University of Oxford Nuffield Department of Orthopaedics Rheumatology and Musculoskeletal Sciences<br>Alser, Osaid ; Harvard Medical School, Trauma, Emergency Surgery and Surgical Critical Care<br>Alghoul, Heba; Islamic University of Gaza Faculty of Medicine,<br>Areia, Carlos; University of Oxford, Nuffield Department of Clinical Neurosciences<br>Dawoud, Dalia; National Institute for Health and Care Excellence,<br>Prats-Uribe, Albert; University of Oxford, Nuffield Department of Orthopaedics Rheumatology and Musculoskeletal Science<br>Valveny, Neus; TFS health science<br>de Maeztu, Gabriel; IOMED<br>Sorlí Redó, Luisa; Universitat Autonoma de Barcelona<br>Martinez Roldan, Jordi; Hospital del Mar<br>Lopez Montesinos, Inmaculada; IMIM<br>Schilling, Lisa; University of Colorado - Anschutz Medical Campus<br>Golozar, Asieh; Johns Hopkins University Bloomberg School of Public Health<br>Reich, Christian; IQVIA<br>Posada, Jose; Stanford University School of Medicine<br>Shah, Nigam ; Stanford University,<br>You, Seng; Yonsei University College of Medicine, Department of Preventive Medicine

	Lynch, Kristine; Department of Veterans Affairs; The University of Utah School of Medicine<br>DuVall, Scott; Department of Veterans Affairs; The University of Utah School of Medicine<br>Matheny, Michael; VA Tennessee Valley Healthcare System, GRECC; Vanderbilt University Medical Center, Department of Biomedical Informatics<br>Nyberg, Fredrik; University of Gothenburg Sahlgrenska Academy, School of Public Health and Community Medicine, Institute of Medicine, Institute of Medicine<br>Ostropolets, Anna; Columbia University Irving Medical Center<br>Hripcsak, George; Columbia University Irving Medical Center<br>Rijnbeek, P; Erasmus Medical Center, Rotterdam<br>Suchard, MA; University of California Los Angeles,<br>Ryan, Patrick; Janssen Research and Development LLC, Observational Health Data Analytics; Columbia University Irving Medical Center, Department of Biomedical Informatics<br>Kostka, Kristin; IQVIA<br>Duarte-Salles, Talita; Institut de Recerca en Atencio Primaria Jordi Gol,
**Primary Subject Heading**:	Epidemiology
Secondary Subject Heading:	General practice / Family practice
Keywords:	COVID-19, EPIDEMIOLOGY, Hypertension < CARDIOLOGY

SCHOLARONE™ Manuscripts

I, the Submitting Author has the right to grant and does grant on behalf of all authors of the Work (as defined in the below author licence), an exclusive licence and/or a non-exclusive licence for contributions from authors who are: i) UK Crown employees; ii) where BMJ has agreed a CC-BY licence shall apply, and/or iii) in accordance with the terms applicable for US Federal Government officers or employees acting as part of their official duties; on a worldwide, perpetual, irrevocable, royalty-free basis to BMJ Publishing Group Ltd ("BMJ") its licensees and where the relevant Journal is co-owned by BMJ to the co-owners of the Journal, to publish the Work in this journal and any other BMJ products and to exploit all rights, as set out in our licence.

The Submitting Author accepts and understands that any supply made under these terms is made by BMJ to the Submitting Author unless you are acting as an employee on behalf of your employer or a postgraduate student of an affiliated institution which is paying any applicable article publishing charge ("APC") for Open Access articles. Where the Submitting Author wishes to make the Work available on an Open Access basis (and intends to pay the relevant APC), the terms of reuse of such Open Access shall be governed by a Creative Commons licence – details of these licences and which Creative Commons licence will apply to this Work are set out in our licence referred to above.

Other than as permitted in any relevant BMJ Author's Self Archiving Policies, I confirm this Work has not been accepted for publication elsewhere, is not being considered for publication elsewhere and does not duplicate material already published. I confirm all authors consent to publication of this Work and authorise the granting of this licence.

For peer review only

Characteristics and outcomes of COVID-19 patients with and without prevalent hypertension: a multinational cohort study

Carlen Reyes[1], Andrea Pistillo[1], Sergio Fernández-Bertolín[1], Martina Recalde[1,2], Elena Roel[1,2], Diana Puente[1,2], Anthony G. Sena[3,4], Clair Blacketer[3,4], Lana Lai[5], Thamir M Alshammari[6], Waheed-UI-Rahman Ahmed[7,8], Osaid Alser[9], Heba Alghoul[10], Carlos Areia[11], Dalia Dawoud[12,13], Albert Prats-Uribe[14], Neus Valveny[15], Gabriel de Maeztu[16], Luisa Sorlí Redó[2,17,18], Jordi Martinez Roldan[19], Inmaculada Lopez Montesinos[17], Lisa M Schilling[20], Asieh Golozar[21,22], Christian Reich[23], Jose D. Posada[24], Nigam H. Shah[24], Seng Chan You[25], Kristine E. Lynch[26,27], Scott L. DuVall[26,27], Michael E. Matheny[26,27], Fredrik Nyberg[28], Anna Ostropolets[29], George Hripcsak[30,31], Peter R. Rijnbeek[32], Mark A. Suchard[33], Patrick Ryan[3,30], Kristin Kostka[23,34], Talita Duarte-Salles[1*]

<u>Affiliations:</u>

1- Fundació Institut Universitari per a la recerca a l'Atenció Primària de Salut Jordi Gol i Gurina (IDIAPJGol), Barcelona, Spain.

2- Universitat Autònoma de Barcelona, Spain

3- Janssen Research & Development, Titusville, NJ, USA

4- Department of Medical Informatics, Erasmus University Medical Center, Rotterdam, The Netherlands

5- School of Medical Sciences, University of Manchester, UK

6- College of Pharmacy, Riyadh Elm University Riyadh, Saudi

7- Nuffield Department of Orthopaedics, Rheumatology, and Musculoskeletal Sciences, University of Oxford, Botnar Research Centre, Windmill Road, Oxford, UK.

For peer review only - http://bmjopen.bmj.com/site/about/guidelines.xhtml

8- College of Medicine and Health, University of Exeter, St Luke’s Campus, Heavitree Road, Exeter, UK

9- Massachusetts General Hospital, Harvard Medical School, USA

10- Faculty of Medicine, Islamic University of Gaza, Palestine

11- Nuffield Department of Clinical Neurosciences, University of Oxford, Oxford, UK

12- National Institute for Health and Care Excellence (NICE), London, UK

13- Cairo University, Faculty of Pharmacy, Cairo, Egypt

14- Centre for Statistics in Medicine, NDORMS, University of Oxford, Botnar Research Centre, Nuffield Orthopaedic Centre, Windmill Road, Oxford, UK

15- Real-World Evidence, TFS, Barcelona, Spain

16- IOMED, Barcelona, Spain

17- Department of Infectious Diseases, Hospital del Mar, Institut Hospital del Mar d'Investigació Mèdica (IMIM), Barcelona, Spain

18- Universitat Pompeu Fabra, Barcelona, Spain

19- Director of Innovation and Digital Transformation, Hospital del Mar, Barcelona, Spain

20- University of Colorado Anschutz Medical Campus, Aurora, CO, USA

21- Regeneron Pharmaceuticals, Tarrytown, NY, USA

22- Johns Hopkins Bloomberg School of Public health, NY, USA

23- Real-World Solutions, IQVIA, Cambridge, MA, USA

24- Stanford University School of Medicine, Stanford, CA, USA

25- Department of Preventive Medicine, Yonsei University College of Medicine, Seoul, Korea

26- VA Informatics and Computing Infrastructure, VA Salt Lake City Health Care System, Salt Lake City, UT, USA

For peer review only

For peer review only - http://bmjopen.bmj.com/site/about/guidelines.xhtml

27- Department of Internal Medicine, University of Utah School of Medicine, Salt Lake City, UT, USA

28- School of Public Health and Community Medicine, Institute of Medicine, Sahlgrenska Academy, University of Gothenburg, Gothenburg, Sweden

29- Columbia University Irving Medical Center, New York, USA

30- Department of Biomedical Informatics, Columbia University Irving Medical Center, New York, NY, USA

31- Medical Informatics Services, NewYork-Presbyterian Hospital, New York, NY, USA

32- Department of Medical Informatics Erasmus University Medical Center, Rotterdam, The Netherlands

33- Department of Biostatistics, Fielding School of Public Health, University of California, Los Angeles, USA

34- The OHDSI Center at the Roux Institute, Northeastern University, Portland, ME, USA

*Corresponding author:

Talita Duarte-Salles

Fundació Institut Universitari per la recerca a L'Atenció Primària de Salut Jordi Gol I Gurina (IDIAPJGol)

Gran Via Corts Catalanes, 587, àtic

08007 Barcelona-Spain

Tel: +34-93 4824342

Email: tduarte@idiapjgol.org

For peer review only

ABSTRACT

Objective: To characterize patients with and without prevalent hypertension and COVID-19, and to assess their adverse outcomes in both in and outpatients.

Design and setting: Retrospective cohort study using 15 healthcare databases (primary and secondary electronic health care records, insurance and national claims data) from the US, Europe and South Korea, standardized to the Observation Medical Outcomes Partnership common data model. Data was gathered from 1st March to 31st October 2020.

Participants: Two non-mutually exclusive cohorts were defined: 1) individuals *diagnosed* with COVID-19 (*diagnosed cohort*) and 2) individuals *hospitalized* with COVID-19 (*hospitalized cohort*) and stratified by hypertension status. Follow-up was from COVID-19 diagnosis/hospitalization to death, end of the study period, or 30-days.

Outcomes: Demographics, comorbidities, and 30-day outcomes (hospitalization and death for the *diagnosed cohort* and adverse events and death for the *hospitalized cohort*) were reported.

Results: We identified 2,851,035 *diagnosed* and 563,708 *hospitalized* patients with COVID-19. Hypertension was more prevalent in the latter (range (%, 95%CI) across databases 17.4 (17.2-17.6)- 61.4 (61.0-61.8) and 25.6 (24.6-26-6)-85.9 (85.2-86.6). Patients in both cohorts with hypertension were predominantly >50-year-old and female. Patients with hypertension were frequently diagnosed with obesity, heart disease, dyslipidaemia, and diabetes. Compared to patients without hypertension, patients with hypertension, in the COVID-19 *diagnosed cohort,* had more hospitalizations (range 1.3 (0.4-2.2)- 41.1 (39.5-42.7) vs 1.4 (0.9-1.9)-15.9 (14.9-16.9)) and mortality (0.3(0.1-0.5)-18.5 (15.7-21.3) vs 0.2 (0.2-0.2)-11.8 (10.8-12.8)). Patients in the COVID-19 *hospitalized cohort* with hypertension were more

For peer review only

For peer review only - http://bmjopen.bmj.com/site/about/guidelines.xhtml

likely to have acute respiratory distress syndrome (0.1(0.0-0.2) -65.6 (62.5-68.7) vs 0.1 (0.0-0.2)-54.7 (50.5-58.9)), arrhythmia (0.5 (0.3-0.7)-45.8 (42.6-49.0) vs 0.4 (0.3-0.5)-36.8 (32.7-40.9)) and increased mortality (1.8 (0.4-3.2)-25.1 (23.0-27.2) vs 0.7 (0.5-0.9)-10.9 (10.4-11.4)) than patients without hypertension.

Conclusions: COVID-19 patients with hypertension were more likely to suffer severe outcomes, hospitalizations and deaths compared to those without hypertension.

KEY WORDS: COVID-19, Epidemiology, Hypertension

WORD COUNT: 3,014

ARTICLE SUMMARY

Strengths and limitations of this study

1- This study is unique in its approach to characterizing COVID-19 cases across an international network of healthcare databases, with diverse healthcare systems and policies, through a comprehensive federated approach.

2- This study was carried out using routinely collected clinical practice data, which confers a great external validity, but also implies a risk of misclassification.

3- This study was intentionally descriptive and was deliberately not designed for causal inference.

4- The diagnosed and/or hospitalized cohorts were non-mutually exclusive.

5- The data that underpinned this study mostly came from the initial months of the COVID-19 pandemic and may not be representative of the COVID-19 cases diagnosed and/or hospitalized during subsequent periods.

For peer review only

INTRODUCTION

As of September 2021, the ongoing pandemic of the coronavirus disease 2019 (COVID-19) has affected over 220 million people and the estimated death toll surpasses the 4,5 million deaths worldwide[1]. Hypertension is a common chronic condition that may increase the risk of hospitalizations and adverse outcomes[2]. A higher prevalence of hypertension has been found among COVID-19 patients compared to the general population, which has attracted the attention of researchers[3]. The characterization of this population at risk is key to be able to design effective preventive strategies that could, improve patient outcomes and reduce the pressure on healthcare systems.

To date, observational studies [4-16], systematic reviews, and meta-analyses have reported an increased risk of progression to severe COVID-19 and increased mortality in patients with hypertension [17-21]. However, these studies, either only included hospitalized patients[4-13,15-16], leading to a selection bias, or had a small sample size [6-10,15], both of which limits the extrapolation of results.

Most patients with confirmed SARS-CoV-2 infection, experience mild or moderate symptoms (80%)[22] and are predominantly seen as outpatients, therefore a large characterization study including both inpatient and outpatients is needed.

This study aims to describe and compare the demographics, baseline comorbidities and 30-day outcomes of individuals with COVID-19 and with and without pre-existing hypertension, in both in and outpatients.

MATERIAL AND METHODS

Study design, setting, and data sources

A multinational, multi-data base cohort study was conducted using data from 1st March to the 31st October 2020 included in “The Characterizing Health Associated Risks and Your

For peer review only - http://bmjopen.bmj.com/site/about/guidelines.xhtml

Baseline Disease In SARS-COV-2" (CHARYBDIS [23]) study. This is a large-scale multinational cohort study aimed to characterize health-associated risks and baseline diseases in SARS-COV-2 patients using routinely collected primary care and hospital electronic health records (EHR), hospital billing, and insurance claims data from the United States (US), Europe (the Netherlands, Spain, the United Kingdom (UK), Germany, and France) and Asia (South Korea and China).

From the databases contributing to CHARYBDIS, only twenty had available information on pre-existing hypertension and were initially selected. To be included in the study, databases had to: 1. have at least 140 subjects with prevalent hypertension diagnosed with COVID-19 (necessary to estimate the prevalence of previous conditions or 30-day outcomes with sufficient precision (confidence interval width of ±5%)) and 2. have at least one year of previous data before the date of COVID-19 diagnosis or hospitalization. Data results for this paper were extracted on the 21st of January 2021 [23]. Fifteen databases complied with the aforementioned inclusion criteria. Of these, five had data for outpatients (IQVIA-Longitudinal Patients Database "LPD" (France), IQVIA-Longitudinal Patients Database "LPD" (Italy), IQVIA-Disease Analyser "DA" (Germany), Clinical Practice Research Datalink "CPRD" (UK), Integrated Primary Care Information "IPCI" (the Netherlands), two had data for in-patients (Health Insurance Review & Assessment Service "HIRA" (South Korea), Hospital del Mar "HMAR" (Spain)) and eight had both in and out-patient data (IQVIA-OpenClaims, HEALTHVERITY, Information System for Research in Primary Care "SIDIAP" (Spain [24]), Optum© de-identified Electronic health Record Dataset "OPTUM-HER" (US), VA-OMOP, University of Colorado Anschutz Medical Campus Health Data Compass "CUIMC" (US), CU-AMC-HDC, STAnford Medicine Research Data Repository

For peer review only

For peer review only - http://bmjopen.bmj.com/site/about/guidelines.xhtml

166 "STARR-OMOP" (US [25])). A more detailed description of the included data sources is available in the Supporting Figure 1 and Table 1.

168 **Study participants and follow-up**

169 Two non-mutually exclusive cohorts were defined: 1) individuals *diagnosed* with COVID-19 (COVID-19 *diagnosed*) and 2) individuals *hospitalized* with COVID-19 (COVID-19 *hospitalized*). COVID-19 *diagnosed* cohort included individuals with a COVID-19 clinical diagnosis and/or a SARS-CoV-2 positive test. The COVID-19 *hospitalized* cohort included patients hospitalized with a COVID-19 clinical diagnosis or positive test 21 days before admission up to the end of their hospitalization. The codes used to identify COVID-19 cases are described in more detail in Supporting Table 2. The index date (i.e. cohort start date) was the date of COVID-19 diagnosis or positive test (whichever occurred first), for the diagnosed cohort; and the date of hospitalization, for the hospitalized cohort. Cohort participants were followed from the index date to the earliest of death, the end of the observation period, or 30 days after.

180 **Baseline characteristics and outcomes of interest**

181 The hypertension diagnosis, as well as the participants' sex and age, were gathered at the index date and identified comorbidities in the year before the index date. Hypertension diagnosis and comorbidities (asthma, cancer, chronic kidney and liver disease, chronic obstructive pulmonary disease, dementia, heart disease, hyperlipidaemia, peripheral vascular disease, type 2 diabetes mellitus, obesity) were ascertained based on the Systematized Nomenclature of Medicine Current Terminology (SNOMED CT) hierarchy, with all descendant codes included. We selected and included comorbidities based on their prevalence in the cohorts of the participating sites and their clinical relevance to the COVID-19 research field [17-21]. Clinical epidemiologists generated a list of codes for the identification of prior medical conditions and outcomes of interest using a web-based integrated platform

For peer review only - http://bmjopen.bmj.com/site/about/guidelines.xhtml

(ATLAS tool: https://atlas.ohdsi.org/). The definition of the variables can be found in Supporting Table 3.

Our main 30-day outcomes of interest were hospitalization and death for the COVID-19 *diagnosed* cohort, and requirement of intensive services (identified as any record of mechanical ventilation and/or a tracheostomy and/or extracorporeal membrane oxygenation procedure), acute respiratory distress syndrome (ARDS), arrhythmia, total cardiovascular events (ischemic stroke, haemorrhagic stroke, heart failure (heart failure during hospitalization for the hospitalized cohort), acute myocardial infarction or sudden cardiac death), sepsis, venous thromboembolism (VTE) and death for the COVID-19 *hospitalized* cohort.

Statistical analyses

All data were standardized to the Observational Medical Outcomes Partnership (OMOP) Common Data Model (CDM) [26]. A common analytical code for the CHARYBDIS study was developed for the Observational Health Data Sciences and Informatics (OHDSI) Methods Library which was run locally in each database. Only aggregate results from each database were publicly shared. The CHARYBDIS protocol and source code can be found at https://github.com/ohdsi-studies/Covid19CharacterizationCharybdis.

Demographics (sex and age categorized in 5-year age bands), comorbidities and 30-day incidence rates of outcomes were reported as proportions, along with 95% Confidence Intervals (CI). A minimum of 5 individuals was established to minimize the risk of identification of patients.

All results are reported by cohort, database and by hypertension status (with or without hypertension).

This is a descriptive study and no causal inference is intended. Multivariable regression or adjustment for confounding was therefore considered out of remit, and not included in our

For peer review only

study. We used R version 4.0.3 for data visualization. Before performing these analyses, all the data partners requested the Institutional Review Board (IRB) or equivalent governance approval. The full ethics committees' statement is detailed in the ethics statement section. All data partners consented to the external sharing of the result set on data.ohdsi.org. Consent to participate was not required as only anonymised retrospective data was used for this study and no patient or GP contact was required.

Patient and Public Involvement

No patient involved

RESULTS

Study population

Overall, 2,851,035 patients diagnosed and 563,708 patients hospitalized with COVID-19 were identified in 15 databases from 8 countries (the US, South Korea, Germany, the Netherlands, France, Italy, Spain, and the UK). In total, 1,408,762 and 427,385 patients diagnosed and hospitalized with COVID-19, respectively, had a prior diagnosis of hypertension (Supporting Table 4). The prevalence of hypertension ranged from 17.4% to 48.3% in the COVID-19 *diagnosed* cohort, and from 25.6% to 85.9% in the COVID-19 *hospitalized cohort*.

Baseline characteristics

The age and sex distribution in the COVID-19 *diagnosed cohort* and in the COVID-19 *hospitalized cohort*, with and without hypertension are represented in Figures 1 and 2 respectively. Overall, in both cohorts, patients with hypertension were older than those without (higher proportion of patients aged above 50 across all databases). The proportion of patients *diagnosed* with COVID-19 and hypertension peaked at a younger age (55 to 70 years old) compared to those *hospitalized* (70 to 80 years old). The proportion of women with

For peer review only - http://bmjopen.bmj.com/site/about/guidelines.xhtml

hypertension was greater in the *diagnosed* cohort (8.6 % to 55.6%) than in the *hospitalized* cohort (4.5% to 56%).

Baseline comorbidities

Figures 3 and 4 reports the proportion of baseline comorbidities of the COVID-19 *diagnosed* cohort (Figure 3) and COVID-19 *hospitalized* cohort (Figure 4), with and without hypertension. Patients with hypertension and COVID-19 *diagnosed* or *hospitalized* were frequently diagnosed with obesity, heart disease, dyslipidaemia, and type 2 diabetes, the proportion of which, more than double the ones found among patients with COVID-19 without hypertension.

30-day outcomes of interest

Thirty-day outcomes in people with and without hypertension in both the COVID-19 *diagnosed* and/or *hospitalized* cohorts are reported in Tables 1 and 2.

Patients with hypertension *diagnosed* with COVID-19 were more likely to be hospitalized (range 1.3% to 41.1% vs 1.4 to 15.9%) and had increased mortality (range 0.3% to 18.5% vs 0.2% to 11.8%) when compared to those without hypertension (Table 1).

Table 1. Comparison of 30-day outcomes of interest between COVID-19 patients with and without hypertension (HTN), in the COVID-19 diagnosed cohorts in the CHARYBDIS Network, % (95%CI)

Database	HTN	N	30-day outcomes	
			Death	Hospitalization
IQVIA-OpenClaims	With	1,245,436	-	29.6 (29.5-29.7)

For peer review only - http://bmjopen.bmj.com/site/about/guidelines.xhtml

For peer review only

(US)				
	Without	1,333,227	-	8.9 (8.9-8.9)
OPTUM-HER (US)	With	6,6432	1.7 (1.6-1.8)	26.4 (26.1-26.7)
	Without	11,1033	0.2 (0.2-0.2)	9.2 (9.0-9.4)
VA-OMOP (US)	With	34,093	5.4 (5.2-5.6)	23.4 (23.0-23.8)
	Without	21,464	0.7 (0.6-0.8)	6.1 (5.8-6.4)
HEALTHVERITY (US)	With	25,405	-	14.6 (14.2-15.0)
	Without	88,768	-	3.1 (3.0-3.2)
SIDIAP (Spain)	With	21,289	9.8 (9.4-10.2)	22.8 (22.2-23.4)
	Without	100,852	3.3 (3.2-3.4)	11.2 (11.0-11.4)
CUIMC (US)	With	3,672	11.8 (10.8-12.8)	41.1 (39.5-42.7)
	Without	4,847	2.0 (1.6-2.4)	15.9 (14.9-16.9)
CU-AMC-HDC (US)	With	2,461	5.9 (5.0-6.8)	35.8 (33.9-37.7)
	Without	4,809	0.7 (0.5-0.9)	11.2 (10.3-12.1)
IQVIA-DA (Germany)	With	2,418	0.3 (0.1-0.5)	-
	Without	5,553	-	-
STARR-OMOP (US)	With	1,246	0.6 (0.2-1.0)	24.6 (22.2-27.0)
	Without	2,082	-	14.0 (12.5-15.5)
CPRD (UK)	With	756	18.5 (15.7-21.3)	-

	Without	2,616	11.8 (10.6-13.0)	-
IPCI (The Netherlands)	With	676	13.6 (11.0-16.2)	1.3 (0.4-2.2)
	Without	2,371	3.1 (2.4-3.8)	1.4 (0.9-1.9)
Note: "-" means information is not available or <5 cases for all databases except for CU-AMC HDC where information is not available for <10 cases.				

Patients with hypertension *hospitalized* with COVID-19 were more frequently diagnosed of ARDS (range 0.1 to 65.6% vs 0.1 to 54.7%), cardiac arrhythmia (range 0.5 to 45.8% vs 0.4 to 36.8%), and had increased mortality (range 1.8 to 25.1% vs 0.7 to 10.9%) as compared to those without hypertension (Table 2).

For peer review only

Table 2. Comparison of 30-days outcomes of interest between COVID-19 patients with and without hypertension (HTN), in the COVID-19 hospitalized cohorts in the CHARYBDIS Network, % (95%CI)

Database	HTN*	N	30-day outcomes						
			VTE†	Death	Cardiac arrhythmia	Sepsis	ARDS‡	Intensive services	Total CVE§
IQVIA-OpenClaims (US)	With	384,508	3.9 (3.8-4.0)	-	15.4 (15.3-15.5)	18.3 (18.2-18.4)	34.8 (34.6-35.0)	9.1 (9.0-9.2)	11.3 (11.2-11.4)
	Without	118,425	3.8 (3.7-3.9)	-	7.2 (7.1-7.3)	15.5 (15.3-15.7)	31.3 (31.0-31.6)	6.4 (6.3-6.5)	4.5 (4.4-4.6)
OPTUM-HER (US)	With	18,242	6.2 (5.9-6.5)	5.1 (4.8-5.4)	31.6 (30.9-32.3)	24.8 (24.2-25.4)	45.7 (45.0-46.4)	14.0 (13.5-14.5)	18.2 (17.6-18.8)
	Without	10,222	4.4 (4.0-4.8)	1.6 (1.4-1.8)	11.1 (10.5-11.7)	15.0 (14.3-15.7)	27.5 (26.6-28.4)	6.3 (5.8-6.8)	4.8 (4.4-5.2)
VA-OMOP (US)	With	8,996	7.3 (6.8-7.8)	15.4 (14.7-16.1)	33.9 (32.9-34.9)	20.0 (19.2-20.8)	43.9 (42.9-44.9)	17.1 (16.3-17.9)	21.0 (20.2-21.8)
	Without	1,475	6.9 (5.6-8.2)	7.6 (6.2-9.0)	16.8 (14.9-18.7)	16.2 (14.3-18.1)	39.6 (37.1-42.1)	11.2 (9.6-12.8)	7.3 (6.0-8.6)
HEALTHVERITY (US)	With	4,512	3.6 (3.1-4.1)	-	14.8 (13.8-15.8)	16.5 (15.4-17.6)	26.7 (25.4-28.0)	6.1 (5.4-6.8)	11.9 (11.0-12.8)
	Without	3,069	3.9 (3.2-4.6)	-	6.8 (5.9-7.7)	12.5 (11.3-13.7)	23.9 (22.4-25.4)	4.9 (4.1-5.7)	5.6 (4.8-6.4)

For peer review only - http://bmjopen.bmj.com/site/about/guidelines.xhtml

For peer review only

SIDIAP (Spain)	With	5,636	1.0 (0.7-1.3)	15.4 (14.5-16.3)	0.5 (0.3-0.7)	-	0.1 (0.0-0.2)	-	0.9 (0.7-1.1)
	Without	12,566	1.1 (0.9-1.3)	10.9 (10.4-11.4)	0.4 (0.3-0.5)	0.0 (0.0-0.0)	0.1 (0.0-0.2)	-	0.5 (0.4-0.6)
CUIMC (US)	With	1,708	3.9 (3.0-4.8)	25.1 (23.0-27.2)	12.1 (10.6-13.6)	6.1 (5.0-7.2)	16.0 (14.3-17.7)	2.2 (1.5-2.9)	8.1 (6.8-9.4)
	Without	892	3.6 (2.4-4.8)	10.2 (8.2-12.2)	4.7 (3.3-6.1)	5.3 (3.8-6.8)	17.8 (15.3-20.3)	1.8 (0.9-2.7)	3.8 (2.5-5.1)
CU-AMC HDC (US)	With	904	11.4 (9.3-13.5)	14.9 (12.6-17.2)	45.8 (42.6-49.0)	34.2 (31.1-37.3)	65.6 (62.5-68.7)	28.3 (25.4-31.2)	19.8 (17.2-22.4)
	Without	530	6.0 (4.0-8.0)	6.0 (4.0-8.0)	36.8 (32.7-40.9)	27.4 (23.6-31.2)	54.7 (50.5-58.9)	15.5 (12.4-18.6)	5.7 (3.7-7.7)
HIRA (South Korea)	With	1,943	0.7 (0.3-1.1)	7.7 (6.5-8.9)	4.4 (3.5-5.3)	5.3 (4.3-6.3)	2.6 (1.9-3.3)	4.9 (3.9-5.9)	10.0 (8.7-11.3)
	Without	5,656	NC	0.7 (0.5-0.9)	0.7 (0.5-0.9)	3.1 (2.6-3.6)	0.5 (0.3-0.7)	0.6 (0.4-0.8)	4.7 (4.1-5.3)
STARR-OMOP (US)	With	342	2.0 (0.5-3.5)	1.8 (0.4-3.2)	22.2 (17.8-26.6)	9.9 (6.7-13.1)	12.6 (9.1-16.1)	9.1 (6.1-12.1)	16.4 (12.5-20.3)
	Without	273	NC	-	6.6 (3.7-9.5)	7.0 (4.0-10.0)	11.4 (7.6-15.2)	5.5 (2.8-8.2)	-
HMAR (Spain)	With	594	3.2 (1.8-4.6)	14.3 (11.5-17.1)	23.1 (19.7-26.5)	1.9 (0.8-3.0)	12.6 (9.9-15.3)	13.5 (10.8-16.2)	12.1 (9.5-14.7)
	Without	1,417	2.6 (1.8-3.4)	3.9 (2.9-4.9)	6.6 (5.3-7.9)	0.7 (0.3-1.1)	7.3 (5.9-8.7)	6.6 (5.3-7.9)	2.2 (1.4-3.0)

Note: "-" means information is not available or <5 cases for all databases except for CU-AMC HDC where information is not available for <10 cases.

For peer review only - http://bmjopen.bmj.com/site/about/guidelines.xhtml

* hypertension; †: Venous thromboembolic (pulmonary embolism and deep vein thrombosis) events; ‡: Acute respiratory distress syndrome; §: cardiovascular disease events (ischemic stroke, haemorrhagic stroke, heart failure (heart failure during hospitalization for the hospitalized cohort), acute myocardial infarction or sudden cardiac death)

For peer review only

For peer review only - http://bmjopen.bmj.com/site/about/guidelines.xhtml

DISCUSSION

This large multinational, multi-database cohort study, reports a greater prevalence of hypertension among patients *hospitalized* with COVID-19 compared to those *diagnosed* with COVID-19. Patients with hypertension diagnosed and/or hospitalized with COVID-19 were frequently diagnosed with obesity, heart disease, dyslipidaemia, and type 2 diabetes at baseline, compared to those without hypertension. They were also more likely to experience adverse outcomes including death and hospitalizations (in the COVID-19 *diagnosed cohort*) and cardiac arrhythmia, ARDS and death (in the COVID-19 *hospitalized* cohort) than patients without hypertension.

This is the first large multinational study that characterizes both in and out-patients with COVID-19, with and without prevalent hypertension. Hypertension was more prevalent in hospitalized patients compared to those diagnosed with COVID-19 (range from 25.6% to 85.9% vs 17.4 to 61.4% respectively). The observed variability between databases is similar to previous reports, where prevalence's ranged from 28.8% [7] to 60% [15]. However, these results should be put into context given that our highest rate (in both COVID-19 *diagnosed* and COVID-19 *hospitalized*) was observed in the VA-OMOP database from the US Department of Veterans Affairs (mostly men of older age).

As in the general population with hypertension [27], patients with hypertension diagnosed with COVID-19 in this study were more frequently diagnosed with heart disease or type 2 diabetes at baseline, than individuals without hypertension. These results are similar to what has been previously published, where patients with hypertension and COVID-19 also reported a higher prevalence of diabetes mellitus [6,8,12], cardiovascular diseases (other than hypertension) [8,12], and chronic kidney disease [8] compared to those without hypertension. This study further expands these previous findings identifying these same comorbidities in the out-patients

For peer review only - http://bmjopen.bmj.com/site/about/guidelines.xhtml

diagnosed with COVID-19 and adds obesity and dyslipidaemia to the list of conditions more frequently found among patients with COVID-19 and hypertension compared to those without hypertension. The higher prevalence of comorbid conditions found in this study among patients with hypertension *hospitalized* with COVID-19 compared to patients with hypertension *diagnosed* with COVID-19 suggests a poorer baseline health status.

Patients with hypertension *hospitalized* with COVID-19 were more likely to have worse disease progression with higher rates of ARDS (Prevalence per cent change (PC) between patients with and without hypertension ranging from -1.8% to 18.2%), more cardiac arrhythmia (PC ranging from 0.1% to 20.5%) and increased mortality (PC ranging from 3.5% to 14.9%). Previous studies have documented poorer clinical outcomes in patients with hypertension hospitalized with COVID-19 (including ARDS) [8, 12, 14, 19], the need for mechanical ventilation, admission to intensive care units [6, 13, 19] or an increased mortality [4,7,11-13, 19]. This study further showed that patients with hypertension *diagnosed* with COVID-19 were more likely to experience hospitalizations (PC between patients with and without hypertension ranging from -0.1% to 25.6%), and deaths (PC from 1.5% to 10.5%).

These results highlight the importance of considering hypertension as a possible risk factor in the overall population *diagnosed* and not only in those *hospitalized* with COVID-19. It also adds to the current literature cardiac arrhythmia and cardiovascular diseases (other than hypertension) to the list of adverse outcomes more frequently diagnosed among patients with hypertension hospitalized with COVID-19 compared to those without hypertension.

This study has several strengths. This is the largest cohort study on individuals with hypertension who were *diagnosed* and/or *hospitalized* with COVID-19 to date. It provides novel insight into the characterization of patients *diagnosed* with COVID-19 and confers a greater external validity of its results compared to what has been published up to date (only *hospitalized* patients). It is also unique in its approach to characterizing COVID-19 cases

For peer review only

For peer review only - http://bmjopen.bmj.com/site/about/guidelines.xhtml

across an international network of healthcare databases, with diverse healthcare systems and policies, through a comprehensive federated approach, allowing the analysis of 15 databases without sharing patient identifiable data, hence respecting the patients' confidentiality at all times.

We recognize there are limitations to our approach. First, this study was intentionally descriptive and was deliberately not designed for causal inference. The observed differences between groups (eg. with versus without hypertension) should therefore not be interpreted as causal effects. Our patients were analysed depending on if they were *diagnosed* and/or *hospitalized* with COVID-19 according to database registration procedures, however, variations could have occurred during the processes by which patients were screened, tested, admitted, and registered across time and the databases. Additionally, the diagnosed and/or hospitalized cohorts were non-mutually exclusive, and therefore could be patients in the diagnosed cohort who were also hospitalized and vice versa.

This study was carried out using data recorded in routine clinical practice based on EHRs and/or claims, therefore, data could be incomplete or be erroneous, leading to potential misclassification. We have therefore selectively reported database-specific outcomes to minimize the impact of incompleteness. Differential reporting in databases is likely due to different coding practices, different primary and secondary level data availability, as well as variability in disease severity, with milder/less symptomatic cases more likely being only diagnosed, and more severe ones hospitalized. Finally, the data that underpinned this study mostly came from the initial months of the COVID-19 pandemic and may not be representative of the COVID-19 cases diagnosed and/or hospitalized during subsequent periods.

CONCLUSIONS

For peer review only - http://bmjopen.bmj.com/site/about/guidelines.xhtml

COVID-19 patients with hypertension are more likely to have comorbidities, experience more severe outcomes including hospitalizations and deaths (among outpatients with COVID-19) and experience more ARDS and deaths (among inpatients' with COVID-19) compared with patients without hypertension.

FIGURE LEGENDS

Figure 1. Comparison of the age and sex distribution in patients with a COVID-19 diagnosis with and without hypertension in the CHARYBDIS Network, %. Colour Red= with hypertension, Green= without hypertension.

Figure 2. Comparison of the age and sex distribution in patients with a COVID-19 hospitalization with and without hypertension in the CHARYBDIS Network, %. Colour Red=with hypertension, Colour Green=without hypertension.

Figure 3. Comorbidities at baseline among patients with a COVID-19 diagnosis with and without hypertension in the CHARYBDIS Network, %. Colour Red=with hypertension, Colour Green=without hypertension.

Figure 4. Comorbidities at baseline among patients with a COVID-19 hospitalization with and without hypertension in the CHARYBDIS Network, %. Colour Red=with hypertension, Colour Green=without hypertension.

For peer review only

CONTRIBUTORSHIP STATEMENT

CR, AGS, CA, APU, AG, FN, AO, GH, PRR, KK, TDS, KEL, SLD, MR, ER, SFB and AP provided substantial contributions to the conception or design, analysis, and interpretation of data for the work. CR, AGS, CA, APU, AG, FN, AO, GH, PRR, KK, TDS, KEL, SLD, MR, ER, SFB, AP, DP, NV, GdeM, LSR, JMR, ILM, NHS, PRy, MAS, MEM, CB, LL, TMA, W-U-RA, OA, HA, DD drafted or revised the manuscript critically for important intellectual

For peer review only - http://bmjopen.bmj.com/site/about/guidelines.xhtml

content. All authors approved the final version of the manuscript and CR, AGS, CA, APU, AG, FN, AO, GH, PRR, KK, TDS, LL, TMA, W-U-RA, OA, HA, DD, LMS, CRei, JDP, SCY agreed to be accountable for all aspects of the work (KEL and SLD only for VA data) in ensuring that questions related to the accuracy or integrity of any part of the work are appropriately investigated and resolved.

COMPETING INTERESTS STATEMENT

SLDV reports grants from Anolinx; MS, reports grants from US National Institutes of Health, grants from Department of Veterans Affairs, during the conduct of the study; grants from IQVIA, personal fees from Janssen Research and Development, grants from US Food and Drug Administration, personal fees from Private Health Management, outside the submitted work LLC, grants from Astellas Pharma, Inc, grants from AstraZeneca Pharmaceuticals LP, grants from Boehringer Ingelheim International GmbH, grants from Celgene Corporation, grants from Eli Lilly and Company, grants from Genentech Inc., grants from Genomic Health, Inc., grants from Gilead Sciences Inc., grants from GlaxoSmithKline PLC, grants from Innocrin Pharmaceuticals Inc., grants from Janssen Pharmaceuticals, Inc., grants from Kantar Health, grants from Myriad Genetic Laboratories, Inc., grants from Novartis International AG, grants from Parexel International Corporation through the University of Utah or Western Institute for Veteran Research, outside the submitted work; GH, reports grants from NIH, during the conduct of the study; grants from Janssen Research, outside the submitted work; FN, reports that Until 2019 was an employee of AstraZeneca and holds some AstraZeneca shares, outside the submitted work; KK, reports personal fees from National Institutes of Health, outside the submitted work, and at the time of data analysis and initial drafting of the manuscript, KK was an employee of IQVIA Inc; CR reports he is an employee of IQVIA Inc; GdM is Employee of IOMED; NV is an Employee of TFS; AGS

For peer review only

reports personal fees from Janssen R&D, outside the submitted work and full time employee of Janssen R&D and is a Johnson and Johnson shareholder; CB reports personal fees from Janssen R&D, outside the submitted work and is a full time employee of Janssen R&D and is a Johnson and Johnson shareholder; JDP reports grants from National LIbrary of Medicine, during the conduct of the study; AG is an employee of Regeneron Pharmaceuticals and reports stocks from Regeneron Pharmaceuticals. PR reports having received research group grants from Innovative Medicine Initiative and Janssen Research and Development; MS reports grants from US National Institutes of Health, grants from Department of Veterans Affairs, during the conduct of the study; grants from IQVIA, personal fees from Janssen Research and Development, grants from US Food and Drug Administration, personal fees from Private Health Management, outside the submitted work; PR, reports and is an employee of Janssen Research and Development and shareholder of Johnson & Johnson; ER, SFB, NHS, LMS, DP, SCY, MR, APU, HA, KEL, MM, AO, CA, CR, TDS, TMA, OA, W-U-RA, ILM, JMR, LSR, DD, LYHL, AP, have nothing to declare. The views expressed are those of the authors and do not necessarily represent the views or policy of the Department of Veterans Affairs or the United States Government. No other relationships or activities could appear to have influenced the submitted work.

FUNDING STATEMENT

This work was supported by several funders as follows; The European Health Data & Evidence Network received funding from the Innovative Medicines Initiative 2 Joint Undertaking (JU) under grant agreement No 806968. The JU received support from the European Union's Horizon 2020 research and innovation programme and EFPIA. This research received partial support from the National Institute for Health Research (NIHR) Oxford Biomedical Research Centre (BRC), US National Institutes of Health (R01

For peer review only

LM006910), US Department of Veterans Affairs, the Health Department from the Generalitat de Catalunya with a grant for research projects on SARS-CoV-2 and COVID-19 disease organized by the Direcció General de Recerca i Innovació en Salut, Janssen Research & Development, TFS, IOMED and IQVIA. The University of Oxford received funding related to this work from the Bill & Melinda Gates Foundation (Investment ID INV-016201 and INV-019257). TFS received funding related to this work from the University of Oxford. This work was also supported with funding, [resources, and facilities] of the Department of Veterans Affairs (VA) Informatics and Computing Infrastructure (VINCI), VA HSR RES 13-457. No funders had a direct role in this study. The views and opinions expressed are those of the authors and do not necessarily reflect those of the Clinician Scientist Award programme, NIHR, Department of Veterans Affairs or the United States Government, NHS, or the Department of Health, England.

For peer review only

DATA SHARING STATEMENT

Open Science is a guiding principle within OHDSI. As such, we provide unfettered access to all open-source analysis tools employed in this study via https://github.com/ohdsi-studies/Covid19CharacterizationCharybdis, as well as all data and results artefacts that do not include patient-level health information via https://data.odhsi.org/Covid19CharacterizationCharybdis/. Data partners contributing to this study remain custodians of their individual patient-level health information and hold either IRB exemption or approval for participation.

ACKNOWLEDGEMENTS

We would like to acknowledge the patients who suffered from or died of this devastating disease, and their families and carers. We also thank the healthcare professionals involved in

For peer review only - http://bmjopen.bmj.com/site/about/guidelines.xhtml

the management of COVID-19 during these challenging times, from primary care to intensive care units. The authors appreciate the Korean Health Insurance Review and Assessment Service for providing data.

We also thank the database curation teams around the world including the COVIDMAR Group (JPHorcajada, R.Güerri, J.Villar, M.Montero, S.Gómez-Zorrilla, M.Arenas-Miras, J.Gómez-Junyent, I.Arrieta, E.Sendra, S.Castañeda, E.Letang, I.Pelegrín, A.Rial, J.Rodríguez, C.Gimenez, J.Soldado, E.García).

We also thank the important contribution to this work of Dr Daniel Prieto-Alhambra.

For peer review only

ETHICS STATEMENT

Before performing these analyses, all the data partners received Institutional Review Board (IRB) approval or exemption. STARR-OMOP had approval from IRB Panel #8 (RB-53248) registered to Leland Stanford Junior University under the Stanford Human Research Protection Program (HRPP). The use of VA-OMOP data was reviewed by the Department of Veterans Affairs Central IRB, was determined to meet the criteria for exemption under Exemption Category 4(3), and approved for Waiver of HIPAA Authorization. The use of SIDIAP was approved by the Clinical Research Ethics Committee of the IDIAPJGol (project code: 20/070-PCV). The use of CPRD was approved by the Independent Scientific Advisory Committee (ISAC) (protocol number 20_059RA2). The use of the CUIMC database was approved by the Columbia University Institutional Review Board as an OHDSI network study (IRB-AAAO7805). The use of HMAR was approved by the Parc de Salut Mar Clinical Research Ethics Committee (Comité de Ética de la Investigación con medicamentos del Parc de Salut MAR, IRB-2020/9183). The use of HIRA database was approved by the IRB of Ajou University (‘AJIRB-MED-EXP-20-061’). The Colorado Multiple Institutional Review Board, CB F490University of Colorado, Anschutz Medical Campus extended an exemption

For peer review only - http://bmjopen.bmj.com/site/about/guidelines.xhtml

of IRB certificate on the 17th of November 2020 for the use of the CU-AMC-HDC data for this study. Moreover, given that this study only used de-identified data with no transmission of patient-level information at any time during the analysis and that all data reported was aggregated and no identification of individual patients or physicians was possible, some databases (IQVIA Open Claims, IQVIA DA Germany, IQVIA LPD France, IQVIA LPD Italy, IPCI) deemed this study as being not human subject research and no further approval was necessary. Furthermore, The New England Institutional Review Board of Janssen Research & Development (Raritan, NJ) has determined that studies conducted on licensed copies of Optum EHR and HealthVerity are exempt from study-specific IRB review, as these studies do not qualify as human subject's research.

REFERENCES

1- Weekly Operational Update on COVID-19-6 Sep 2021 [internet]. WHO [cited 9th September 2021]. Available from https://www.who.int/publications/m/item/weekly-operational-update-on-covid-19---6-september-2021.

2- Unger T, Borghi C, Charchar F, Khan NA, Poulter NR, Prabhakaran D, et al. 2020 International Society of Hypertension global hypertension practice guidelines. J Hypertens. 2020;38:982-1004.

3-Cook TM. The importance of hypertension as a risk factor for severe illness and mortality in COVID-19. Anaesthesia. 2020;75:976-977.

4-Gupta S, Hayek SS, Wang W, Chan L, Mathews KS, Melamed ML, et al. Factors Associated With Death in Critically Ill Patients With Coronavirus Disease 2019 in the US. JAMA Intern Med. 2020;180:1–12.

5-Jiménez E, Fontán-Vela M, Valencia J, Fernandez-Jimenez I, Álvaro-Alonso EA, Izquierdo-García E, et al. Characteristics, complications and outcomes among 1549 patients

For peer review only

For peer review only - http://bmjopen.bmj.com/site/about/guidelines.xhtml

hospitalised with COVID-19 in a secondary hospital in Madrid, Spain: a retrospective case series study. BMJ Open. 2020;10:e042398.

6-Huang S, Wang J, Liu F, Liu J, Cao G, Yang C, et al. COVID-19 patients with hypertension have more severe disease: a multicenter retrospective observational study. Hypertens Res. 2020;43:824-831.

7-Park BE, Lee JH, Park HK, Kim HN, Jang SY, Bae MH, et al. Impact of Cardiovascular Risk Factors and Cardiovascular Diseases on Outcomes in Patients Hospitalized with COVID-19 in Daegu Metropolitan City. J Korean Med Sci. 2021;36:e15.

8- Yao Q, Ni J, Hu TT, Cai ZL, Zhao JH, Xie QW, et al. Clinical characteristics and outcomes in coronavirus disease 2019 (COVID-19) patients with and without hypertension: a retrospective study. Rev Cardiovasc Med. 2020;21:615-625.

9- Zhou F, Yu T, Du R, Fan G, Liu Y, Liu Z, et al. Clinical course and risk factors for mortality of adult inpatients with COVID-19 in Wuhan, China: a retrospective cohort study. Lancet. 2020;395(10229):1054-1062.

10-Wang Y, Lu X, Li Y, Chen H, Chen T, Su N, et al. Clinical Course and Outcomes of 344 Intensive Care Patients with COVID-19. Am J Respir Crit Care Med. 2020;201:1430-1434.

11-Grasselli G, Zangrillo A, Zanella A, Antonelli M, Cabrini L, Castelli A, et al. Baseline Characteristics and Outcomes of 1591 Patients Infected With SARS-CoV-2 Admitted to ICUs of the Lombardy Region, Italy. JAMA. 2020;323:1574-1581.

12-Gao C, Cai Y, Zhang K, Zhou L, Zhang Y, Zhang X, et al. Association of hypertension and antihypertensive treatment with COVID-19 mortality: a retrospective observational study. Eur Heart J. 2020;41:2058-2066.

For peer review only - http://bmjopen.bmj.com/site/about/guidelines.xhtml

13- Guan WJ, Liang WH, Zhao Y, Liang HR, Chen ZS, Li YM, et al. Comorbidity and its impact on 1590 patients with COVID-19 in China: a nationwide analysis. Eur Respir J. 2020;55:2000547.

14- Ji W, Huh K, Kang M, Hong J, Bae GH, Lee R, et al. Effect of Underlying Comorbidities on the Infection and Severity of COVID-19 in Korea: a Nationwide Case-Control Study. J Korean Med Sci. 2020;35:e237.

15- Chilimuri S, Sun H, Alemam A, Mantri N, Shehi E, Tejada J, et al. Predictors of Mortality in Adults Admitted with COVID-19: Retrospective Cohort Study from New York City. West J Emerg Med. 2020;21:779-784.

16- Richardson S, Hirsch JS, Narasimhan M, Crawford JM, McGinn T, Davidson KW, et al. Presenting Characteristics, Comorbidities, and Outcomes Among 5700 Patients Hospitalized With COVID-19 in the New York City Area. JAMA. 2020;323:2052-2059. Erratum in: JAMA. 2020;323:2098.

17-Dorjee K, Kim H, Bonomo E, Dolma R. Prevalence and predictors of death and severe disease in patients hospitalized due to COVID-19: A comprehensive systematic review and meta-analysis of 77 studies and 38,000 patients. PLoS One. 2020;15:e0243191.

18-Javanmardi F, Keshavarzi A, Akbari A, Emami A, Pirbonyeh N. Prevalence of underlying diseases in dead cases of COVID-19: A systematic review and meta-analysis. PLoS One. 2020;15:e0241265.

19-Pranata R, Lim MA, Huang I, Raharjo SB, Lukito AA. Hypertension is associated with increased mortality and severity of disease in COVID-19 pneumonia: A systematic review, meta-analysis and meta-regression. J Renin Angiotensin Aldosterone Syst. 2020;21:1470320320926899.

For peer review only

For peer review only - http://bmjopen.bmj.com/site/about/guidelines.xhtml

20- Silverio A, Di Maio M, Citro R, Esposito L, Iuliano G, Bellino M, et al. Cardiovascular risk factors and mortality in hospitalized patients with COVID-19: systematic review and meta-analysis of 45 studies and 18,300 patients. BMC Cardiovasc Disord. 2021;21:23.

21- Moazzami B, Chaichian S, Kasaeian A, Djalalinia S, Akhlaghdoust M, Eslami M, et al. Metabolic risk factors and risk of Covid-19: A systematic review and meta-analysis. PLoS One. 2020;15:e0243600.

22- Baj J, Karakuła-Juchnowicz H, Teresiński G, Buszewicz G, Ciesielka M, Sitarz E, et al. COVID-19: Specific and Non-Specific Clinical Manifestations and Symptoms: The Current State of Knowledge. J Clin Med. 2020;9:1753.

23- Prieto-Alhambra D, Kostka K, Duarte-Salles T, Prats-Uribe A, Sena A, Pistillo A, et al. Unraveling COVID-19: a large-scale characterization of 4.5 million COVID-19 cases using CHARYBDIS. Res Sq [Preprint]. 2021:rs.3.rs-279400.

24- García-Gil M del M, Hermosilla E, Prieto-Alhambra D, Fina F, Rosell M, Ramos R, et al. Construction and validation of a scoring system for the selection of high-quality data in a Spanish population primary care database (SIDIAP). Inform Prim Care. 2011; 19(3): 135–45

25-Datta S, Posada J, Olson G, Li W, O'Reilly C, Balraj D, et al. A new paradigm for accelerating clinical data science at Stanford Medicine. arXiv:2003.10534

26-Voss EA, Makadia R, Matcho A, Ma Q, Knoll C, Schuemie M, et al. Feasibility and utility of applications of the common data model to multiple, disparate observational health databases. J Am Med Inform Assoc. 2015;22:553-64.

27-Tsimihodimos V, Gonzalez-Villalpando C, Meigs JB, Ferrannini E. Hypertension and Diabetes Mellitus: Coprediction and Time Trajectories. Hypertension. 2018;71:422-428.

For peer review only

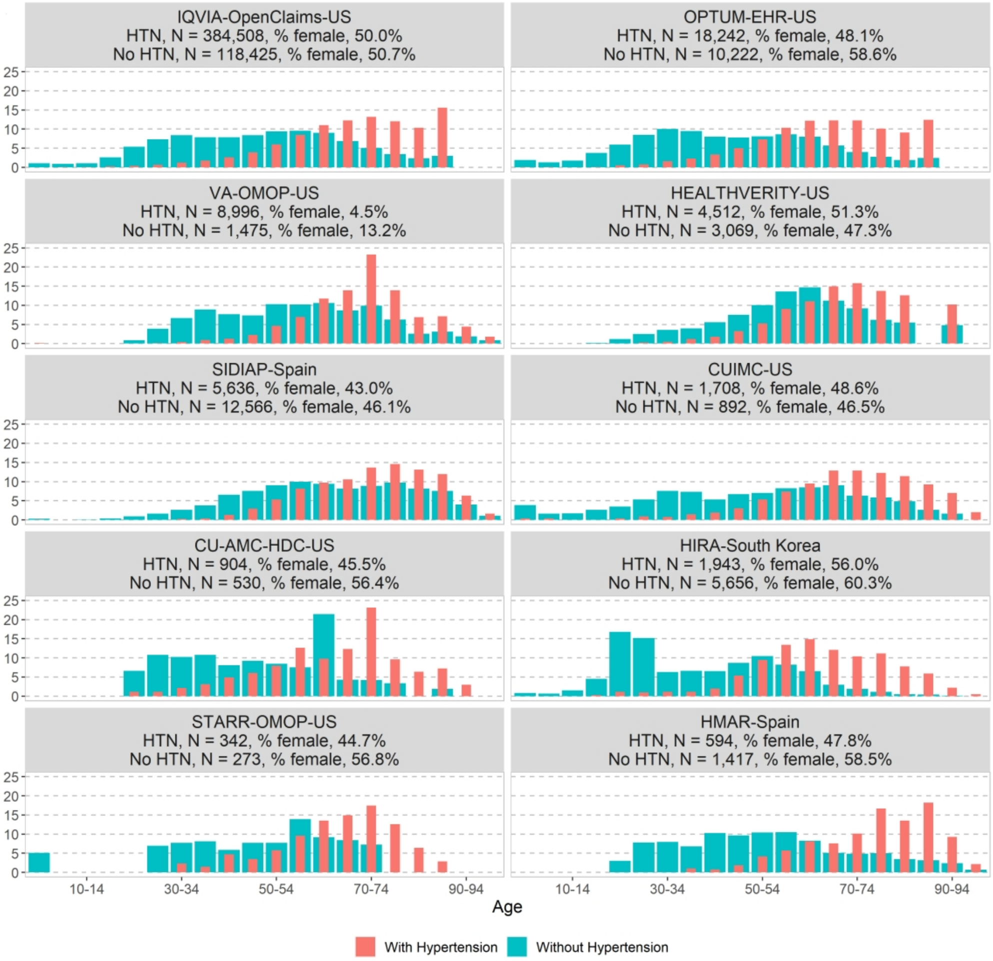

Figure 1. Comparison of the age and sex distribution in patients with a COVID-19 diagnosis with and without hypertension in the CHARYBDIS Network, %. Colour Red= with hypertension, Green= without hypertension.

729x710mm (118 x 118 DPI)

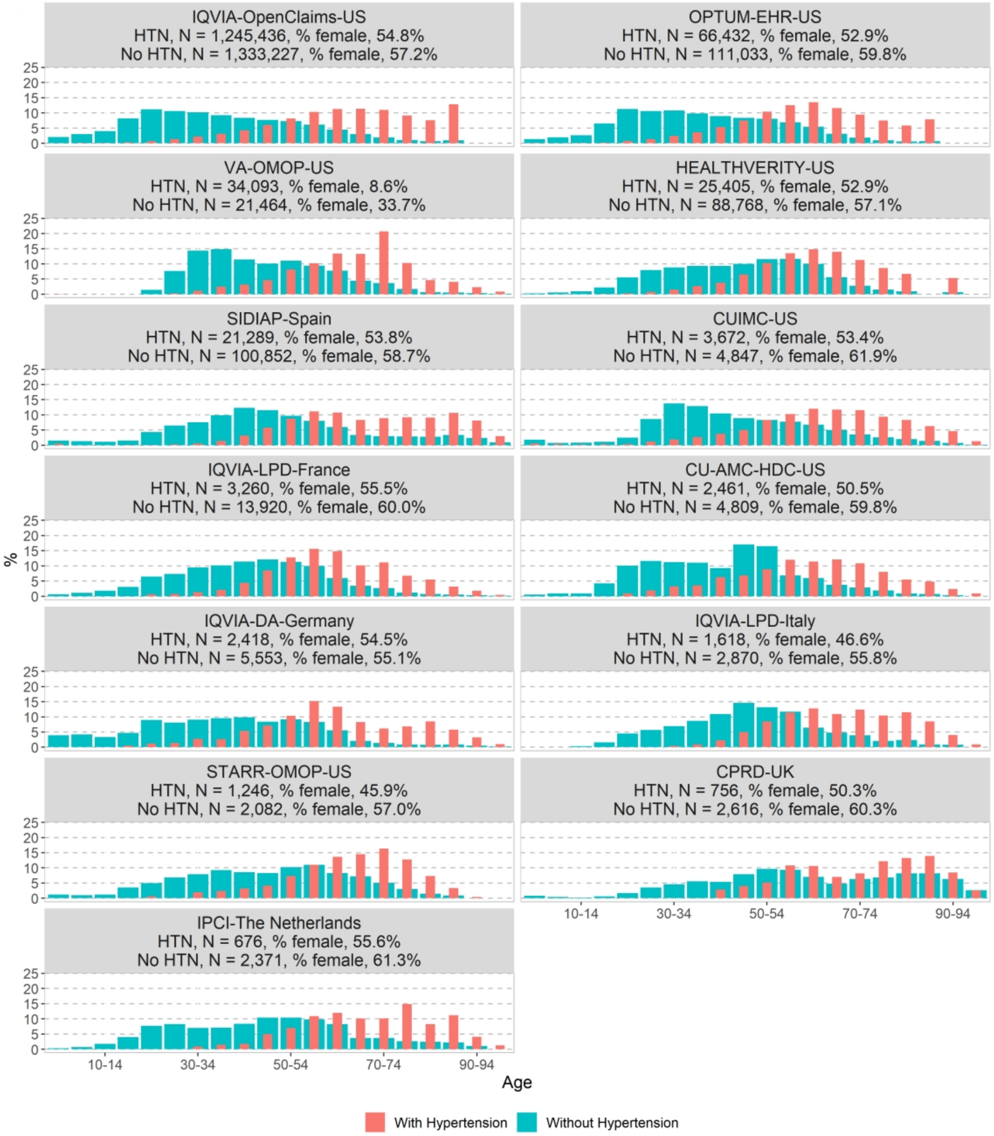

Figure 2. Comparison of the age and sex distribution in patients with a COVID-19 hospitalization with and without hypertension in the CHARYBDIS Network, %. Colour Red=with hypertension, Colour Green=without hypertension.

729x839mm (118 x 118 DPI)

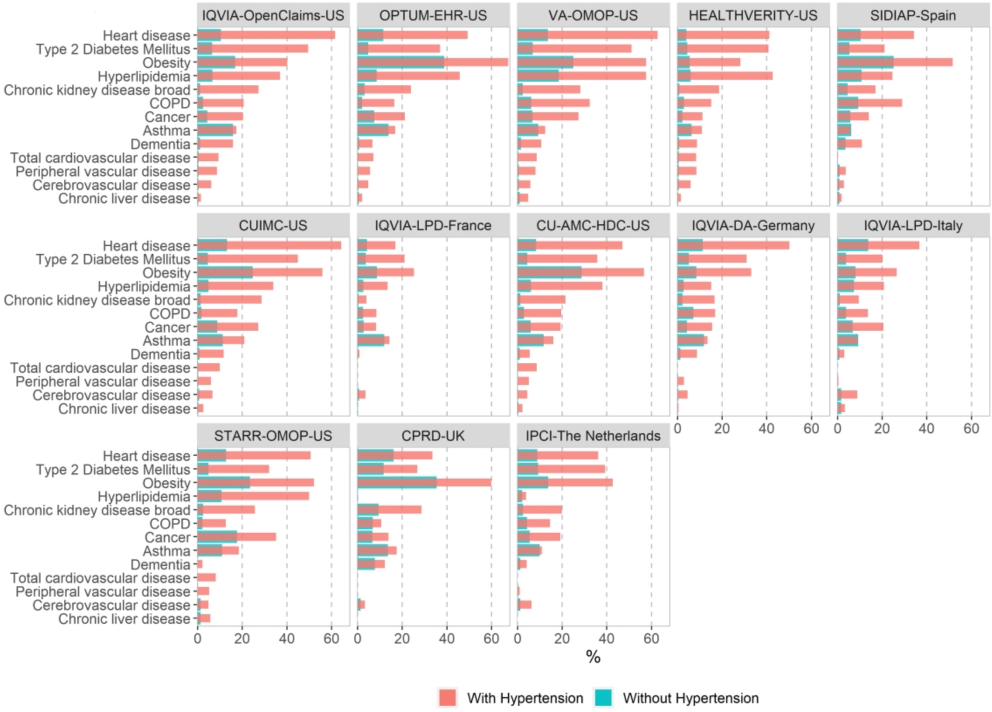

Figure 3. Comorbidities at baseline among patients with a COVID-19 diagnosis with and without hypertension in the CHARYBDIS Network, %. Colour Red=with hypertension, Colour Green=without hypertension.

710x516mm (118 x 118 DPI)

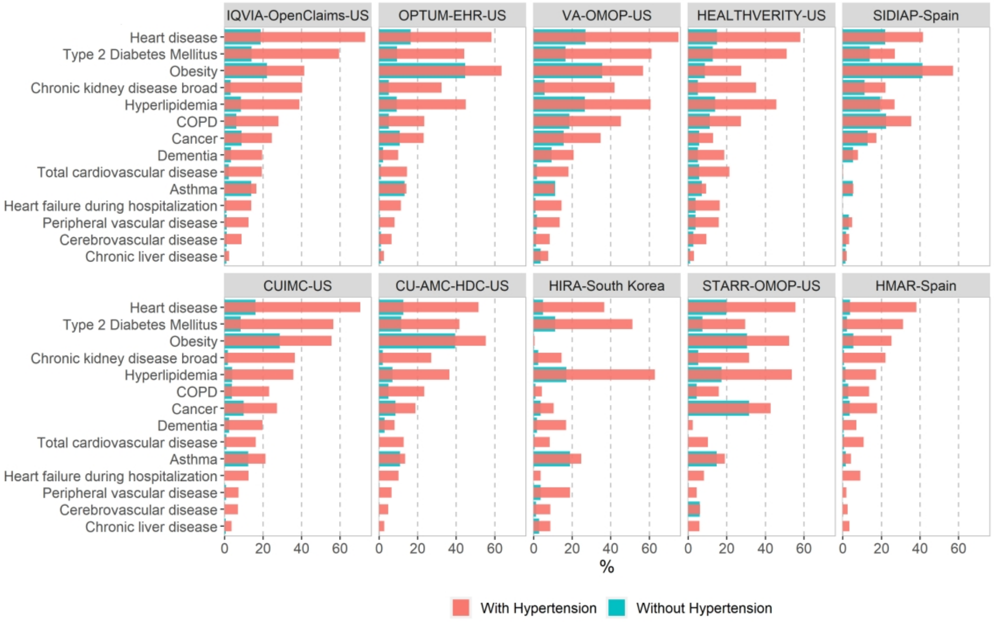

Figure 4. Comorbidities at baseline among patients with a COVID-19 hospitalization with and without hypertension in the CHARYBDIS Network, %. Colour Red=with hypertension, Colour Green=without hypertension.

710x452mm (118 x 118 DPI)

Supporting Figure 1. Flowchart showing the selection of databases included in the analyses

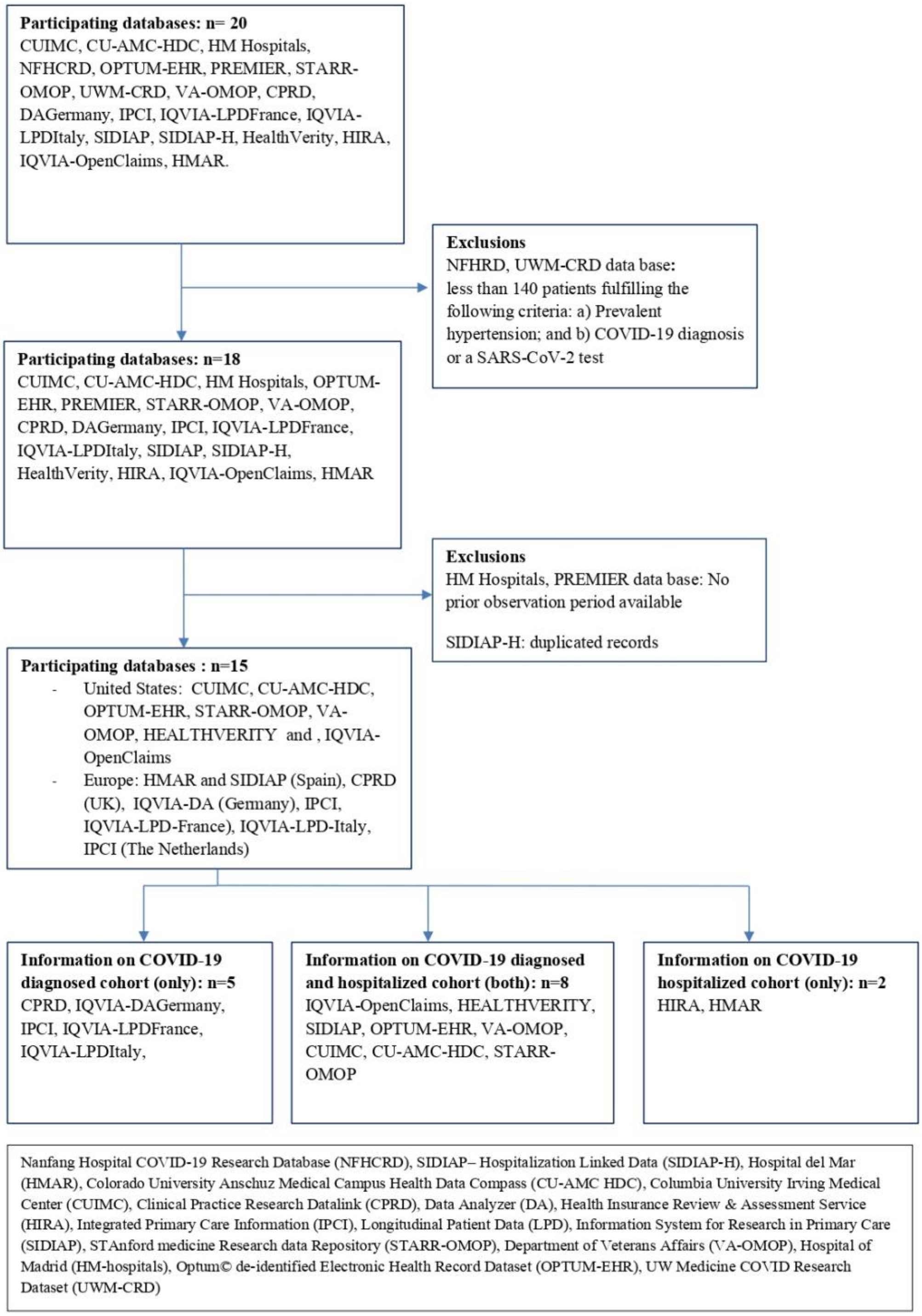

Supporting Table 1. Description of included databases

**Institution Name/ Database**	**Database Description**	**Country**
Janssen Research & Development<br>The Clinical Practice Research Datalink (CPRD)	The Clinical Practice Research Datalink (CPRD) is a governmental not-for-profit research service jointly funded by the NHS National Institute for Health Research (NIHR) and the Medicines and Healthcare products Regulatory Agency (MHRA) a part of the Department of Health United Kingdom (UK). CPRD consists of data collected from UK primary care for all ages. This includes conditions observations measurements and procedures that the general practitioner is made aware of in addition to any prescriptions as prescribed by the general practitioner. In addition to primary care there are also linked secondary care records for a small number of people. The major data elements contained within this database are outpatient prescriptions given by the general practitioner (coded with Multilex codes) and outpatient clinical referral immunization or test events that the general practitioner knows about (coded in Read or ICD10 or LOINC codes). The database also contains the patients' year of births and any date of deaths.	United Kingdom
IDIAPJGol<br>The Information System for Research in Primary Care (SIDIAP)	The Information System for Research in Primary Care (SIDIAP; www.sidiap.org) is a primary care records database that covers approximately 80% of the population of Catalonia North-East Spain. Healthcare is universal and tax-payer funded in the region and primary care physicians are gatekeepers for all care and responsible for repeat prescriptions.	Spain
Stanford Medicine Research Data Repository (STARR-OMOP)	A clinical data warehouse containing live Epic data from Stanford Health Care the Stanford Children's Hospital	United States

For peer review only

	the University Healthcare Alliance and Packard Children's Health Alliance clinics.<br>Reference: Datta S Posada J Olson G *et al.* A new paradigm for accelerating clinical data science at Stanford Medicine. *arXiv* 2020; published online March 17. http://arxiv.org/abs/2003.10534 (accessed Aug 20 2020).	
Columbia University Irving Medical Center (CUIMC)	The clinical data warehouse of New York-Presbyterian Hospital/Columbia University Irving Medical Center New York NY based on its current and previous electronic health record systems with data spanning over 30 years and including over 6 million patients	United States
IQVIA<br>Open Claims	Pre-adjudicated claims covering over 300 Million lives (~80% of the US) collected from office-based physicians and specialists via office management software and clearinghouse switch sources for the purpose of reimbursement.	United States
HIRA<br>Health Insurance Review & Assessment Service	National claim data from a single insurance service from South Korea, It contains the observational medical records (including both inpatient and outpatient) of a patient while they are qualified to get the national medical insurance.	South Korea
HMAR<br>Hospital del mar	Anonymized data from the Electronic Medical Records from Hospital del Mar (Barcelona, Spain). Hospital belonging to the Spanish National Health System (public), attending the Eastern area of Barcelona City. Includes hospital data collected routinely in the clinical practice, both structured and unstructured information, extracted using a free text analysis tool (with natural language processing): Inpatient (hospital) care, Outpatient specialist care, Emergency Room Visits and partial information from other settings like primary care and pharmacy care present in free text notes from EMRs. All subjects with at least one healthcare encounter with the Hospital within approximately last 20 years are included (approximately 0.6 M subjects, with more than	Spain

For peer review only

	5 M hospitalizations/visits). Hospital del Mar data are made available through collaboration with TFS / IOMED.	
OPTUM-EHR<br>Optum® de-identified Electronic Health Record Dataset	Optum ® de-identified Electronic Health Record Dataset is derived from dozens of healthcare provider organizations in the United States (that include more than 700 hospitals and 7,000 Clinics treating more than 103 million patients) receiving care in the United States. The medical record data includes clinical information, inclusive of prescriptions as prescribed and administered, lab results, vital signs, body measurements, diagnoses, procedures, and information derived from clinical Notes using Natural Language Processing (NLP)	United States
IPCI<br>Integrated Primary Care Information	The Integrated Primary Care Information (IPCI) database is collected from EHR records of patients registered with 391 GPs throughout the Netherlands. The database contains records from approximately 2.6 million patients out of a Dutch population of 17M (8.2%) starting in 1996.	The Netherlands
DA Germany<br>IQVIA Disease Analyser Germany	IQVIA DA Germany is collected from extracts of patient management software used by GPs and specialists practicing in ambulatory care settings. Data coverage includes more than 34M distinct person records out of at total population of 80M (42.5%) in the country and collected from 2,734 providers. Dates of service include from 1992 through March 2020	Germany
LPD-Italy<br>IQVIA LPD Italy	LPD Italy is comprised of anonymised patient records collected from software used by GPs during an office visit to document patients' clinical records. Data coverage includes over 2M patient records with at least one visit and 119.5M prescription orders across 900 GP practices. Dates of service include from 2004 through	Italy

For peer review only - http://bmjopen.bmj.com/site/about/guidelines.xhtml

	present. Observation time is defined by the first and last consultation dates. Drugs are captured as prescription records with product, quantity, dosing directions, strength, indication and date of consultation.	
LPD-France	LPD France is a computerised network of physicians including GPs who contribute to a centralised database of anonymised patient EMR. Currently, >1200 GPs from 400 practices are contributing to the database covering 7.8M patients in France. The database covers a time period from 1994 through the present. Observation time is defined by the first and last consultation dates. Drug information is derived from GP prescriptions. Drugs obtained over the counter by the patient outside the prescription system are not reported	France
HEALTHVERITY	This HealthVerity derived data set contains de-identified patient information with an antibody and/or diagnostic test for COVID-19 linked to all available Medical Claims and Pharmacy Data from select private data providers participating in the HealthVerity marketplace.	United States
University of Colorado Anschuz Medical Campus Health Data Compass (CU-AMC HDC)	Health Data Compass (HDC) is a multi-institutional data warehouse. HDC contains inpatient and outpatient electronic medical data including patient, encounter, diagnosis, procedures, medications, laboratory results from two electronic medical record systems (UCHealth and Children's Hospital of Colorado), state-level all-payers claims data, and the Colorado death registry. Acknowledgement statement: Supported by the Health Data Compass Data Warehouse project (healthdatacompass.org)	
Department of Veterans Affairs VA- OMOP	VA-OMOP data reflects the national Department of Veterans Affairs health care system which is the largest integrated provider of medical and mental health services	United States

For peer review only

	in the United States. Care is provided at 170 VA Medical Centers and 1 063 outpatient sites serving more than 9 million enrolled Veterans each year.	

Supporting Table 2. Definitions and codes used to identify COVID-19 cases

The below tables summarises the concepts used to identify patients diagnosed with COVID-19 . The full description of the logic used to identify patients diagnosed and hospitalized is provided at https://atlas.ohdsi.org/#/cohortdefinition/200 and https://atlas.ohdsi.org/#/cohortdefinition/197 respectively.

COVID-19 condition codes

Id	Name	Vocabulary
756023	Acute bronchitis due to COVID-19	OMOP Extension
756044	Acute respiratory distress syndrome (ARDS) due to COVID-19	OMOP Extension
756061	Asymptomatic COVID-19	OMOP Extension
756031	Bronchitis due to COVID-19	OMOP Extension
439676	Coronavirus infection	SNOMED
37311061	Disease caused by 2019-nCoV	SNOMED
4100065	Disease due to Coronaviridae	SNOMED
37310284	Encephalopathy caused by 2019 novel coronavirus	SNOMED

For peer review only

For peer review only - http://bmjopen.bmj.com/site/about/guidelines.xhtml

37310283	Gastroenteritis caused by 2019 novel coronavirus	SNOMED
4248811	Healthcare associated severe acute respiratory syndrome	SNOMED
756081	Infection of lower respiratory tract due to COVID-19	OMOP Extension
37310286	Infection of upper respiratory tract caused by 2019 novel coronavirus	SNOMED
45763594	Middle East respiratory syndrome	SNOMED
37310287	Myocarditis caused by 2019 novel coronavirus	SNOMED
37310254	Otitis media caused by 2019 novel coronavirus	SNOMED
37310285	Pneumonia caused by 2019 novel coronavirus	SNOMED
37016927	Pneumonia caused by Human coronavirus	SNOMED
40479642	Pneumonia due to Severe acute respiratory syndrome coronavirus	SNOMED
756039	Respiratory infection due to COVID-19	OMOP Extension
320651	Severe acute respiratory syndrome	SNOMED
37396171	Severe acute respiratory syndrome of upper respiratory tract	SNOMED
37311060	Suspected disease caused by 2019-nCoV	SNOMED
**COVID-19 specific testing - Positive**		
37310282	2019 novel coronavirus detected	SNOMED
**COVID-19 specific testing (note these required a corresponding value as concept of: Detected Positive or Present)**		

For peer review only

37310255	Detection of 2019 novel coronavirus using polymerase chain reaction technique	SNOMED
700360	Infectious agent detection by nucleic acid (DNA or RNA); severe acute respiratory syndrome coronavirus 2 (SARS-CoV-2) (Coronavirus disease [COVID-19]) amplified probe technique	CPT4
37310258	Measurement of 2019 novel coronavirus antibody	SNOMED
37310257	Measurement of 2019 novel coronavirus antigen	SNOMED
756055	Measurement of Severe acute respiratory syndrome coronavirus 2 (SARS-CoV-2)	OMOP Extension
586310	Measurement of Severe acute respiratory syndrome coronavirus 2 (SARS-CoV-2) Genetic material using Molecular method	OMOP Extension
704991	Measurement of Severe acute respiratory syndrome coronavirus 2 (SARS-CoV-2) in Blood	OMOP Extension
756029	Measurement of Severe acute respiratory syndrome coronavirus 2 (SARS-CoV-2) in Respiratory specimen	OMOP Extension
586307	Measurement of Severe acute respiratory syndrome coronavirus 2 (SARS-CoV-2) in Saliva	OMOP Extension
705107	Measurement of Severe acute respiratory syndrome coronavirus 2 (SARS-CoV-2) in Sample from nose	OMOP Extension
586309	Measurement of Severe acute respiratory syndrome coronavirus 2 (SARS-CoV-2) in Specified specimen	OMOP Extension
756065	Measurement of Severe acute respiratory syndrome coronavirus 2 (SARS-CoV-2) in Unspecified specimen	OMOP Extension
704992	Measurement of Severe acute respiratory syndrome coronavirus 2 (SARS-CoV-2) using Culture method	OMOP Extension
705001	Measurement of Severe acute respiratory syndrome coronavirus 2 (SARS-CoV-2) using Nucleic acid amplification technique	OMOP Extension
705000	Measurement of Severe acute respiratory syndrome coronavirus 2 (SARS-CoV-2) using Nucleic acid amplification technique in Blood	OMOP Extension
756085	Measurement of Severe acute respiratory syndrome coronavirus 2 (SARS-CoV-2) using Nucleic acid amplification technique in Respiratory specimen	OMOP Extension
586308	Measurement of Severe acute respiratory syndrome coronavirus 2 (SARS-CoV-2) using Nucleic acid amplification technique in Saliva	OMOP Extension
705106	Measurement of Severe acute respiratory syndrome coronavirus 2 (SARS-CoV-2) using Nucleic acid amplification technique in Sample from nose	OMOP Extension
756084	Measurement of Severe acute respiratory syndrome coronavirus 2 (SARS-CoV-2) using Nucleic acid amplification technique in Unspecified specimen	OMOP Extension
704993	Measurement of Severe acute respiratory syndrome coronavirus 2 (SARS-CoV-2) using Sequencing	OMOP Extension

For peer review only

586516	SARS-CoV-2 (COVID19) [Presence] in Unspecified specimen by Organism specific culture	LOINC
723480	SARS-CoV-2 (COVID19) Ab [Interpretation] in Serum or Plasma	LOINC
586515	SARS-CoV-2 (COVID19) Ab [Presence] in Serum or Plasma by Immunoassay	LOINC
586522	SARS-CoV-2 (COVID19) Ab [Units/volume] in Serum or Plasma by Immunoassay	LOINC
706179	SARS-CoV-2 (COVID19) Ab panel - Serum or Plasma by Immunoassay	LOINC
723477	SARS-CoV-2 (COVID19) Ag [Presence] in Respiratory specimen by Rapid immunoassay	LOINC
706166	SARS-CoV-2 (COVID19) E gene [Cycle Threshold #] in Unspecified specimen by NAA with probe detection	LOINC
586523	SARS-CoV-2 (COVID19) E gene [Presence] in Respiratory specimen by NAA with probe detection	LOINC
586518	SARS-CoV-2 (COVID19) E gene [Presence] in Serum or Plasma by NAA with probe detection	LOINC
706174	SARS-CoV-2 (COVID19) E gene [Presence] in Unspecified specimen by NAA with probe detection	LOINC
723473	SARS-CoV-2 (COVID19) IgA Ab [Presence] in Serum or Plasma by Immunoassay	LOINC
586521	SARS-CoV-2 (COVID19) IgA Ab [Presence] in Serum Plasma or Blood by Rapid immunoassay	LOINC
723459	SARS-CoV-2 (COVID19) IgA Ab [Units/volume] in Serum or Plasma by Immunoassay	LOINC
757686	SARS-CoV-2 (COVID19) IgA+IgM [Presence] in Serum or Plasma by Immunoassay	LOINC
586527	SARS-CoV-2 (COVID19) IgG Ab [Presence] in DBS by Immunoassay	LOINC
723474	SARS-CoV-2 (COVID19) IgG Ab [Presence] in Serum or Plasma by Immunoassay	LOINC
706181	SARS-CoV-2 (COVID19) IgG Ab [Presence] in Serum Plasma or Blood by Rapid immunoassay	LOINC
706177	SARS-CoV-2 (COVID19) IgG Ab [Units/volume] in Serum or Plasma by Immunoassay	LOINC
706176	SARS-CoV-2 (COVID19) IgG and IgM panel - Serum Plasma or Blood by Rapid immunoassay	LOINC
723479	SARS-CoV-2 (COVID19) IgG+IgM Ab [Presence] in Serum or Plasma by Immunoassay	LOINC

For peer review only - http://bmjopen.bmj.com/site/about/guidelines.xhtml

723475	SARS-CoV-2 (COVID19) IgM Ab [Presence] in Serum or Plasma by Immunoassay	LOINC
706180	SARS-CoV-2 (COVID19) IgM Ab [Presence] in Serum Plasma or Blood by Rapid immunoassay	LOINC
706178	SARS-CoV-2 (COVID19) IgM Ab [Units/volume] in Serum or Plasma by Immunoassay	LOINC
706167	SARS-CoV-2 (COVID19) N gene [Cycle Threshold #] in Unspecified specimen by NAA with probe detection	LOINC
706157	SARS-CoV-2 (COVID19) N gene [Cycle Threshold #] in Unspecified specimen by Nucleic acid amplification using CDC primer-probe set N1	LOINC
706155	SARS-CoV-2 (COVID19) N gene [Cycle Threshold #] in Unspecified specimen by Nucleic acid amplification using CDC primer-probe set N2	LOINC
715272	SARS-CoV-2 (COVID19) N gene [Presence] in Nasopharynx by NAA with probe detection	LOINC
757678	SARS-CoV-2 (COVID19) N gene [Presence] in Nose by NAA with probe detection	LOINC
706161	SARS-CoV-2 (COVID19) N gene [Presence] in Respiratory specimen by NAA with probe detection	LOINC
586524	SARS-CoV-2 (COVID19) N gene [Presence] in Respiratory specimen by Nucleic acid amplification using CDC primer-probe set N1	LOINC
586525	SARS-CoV-2 (COVID19) N gene [Presence] in Respiratory specimen by Nucleic acid amplification using CDC primer-probe set N2	LOINC
586520	SARS-CoV-2 (COVID19) N gene [Presence] in Serum or Plasma by NAA with probe detection	LOINC
706175	SARS-CoV-2 (COVID19) N gene [Presence] in Unspecified specimen by NAA with probe detection	LOINC
706156	SARS-CoV-2 (COVID19) N gene [Presence] in Unspecified specimen by Nucleic acid amplification using CDC primer-probe set N1	LOINC
706154	SARS-CoV-2 (COVID19) N gene [Presence] in Unspecified specimen by Nucleic acid amplification using CDC primer-probe set N2	LOINC
757680	SARS-CoV-2 (COVID19) neutralizing antibody [Presence] in Serum by pVNT	LOINC
757679	SARS-CoV-2 (COVID19) neutralizing antibody [Titer] in Serum by pVNT	LOINC
723469	SARS-CoV-2 (COVID19) ORF1ab region [Cycle Threshold #] in Respiratory specimen by NAA with probe detection	LOINC
706168	SARS-CoV-2 (COVID19) ORF1ab region [Cycle Threshold #] in Unspecified specimen by NAA with probe detection	LOINC
723478	SARS-CoV-2 (COVID19) ORF1ab region [Presence] in Respiratory specimen by NAA with probe detection	LOINC

For peer review only

723464	SARS-CoV-2 (COVID19) ORF1ab region [Presence] in Unspecified specimen by NAA with probe detection	LOINC
723471	SARS-CoV-2 (COVID19) RdRp gene [Cycle Threshold #] in Respiratory specimen by NAA with probe detection	LOINC
723470	SARS-CoV-2 (COVID19) RdRp gene [Cycle Threshold #] in Unspecified specimen by NAA with probe detection	LOINC
706160	SARS-CoV-2 (COVID19) RdRp gene [Presence] in Respiratory specimen by NAA with probe detection	LOINC
706173	SARS-CoV-2 (COVID19) RdRp gene [Presence] in Unspecified specimen by NAA with probe detection	LOINC
586528	SARS-CoV-2 (COVID19) RNA [Cycle Threshold #] in Respiratory specimen by NAA with probe detection	LOINC
586529	SARS-CoV-2 (COVID19) RNA [Cycle Threshold #] in Unspecified specimen by NAA with probe detection	LOINC
715262	SARS-CoV-2 (COVID19) RNA [Log #/volume] (viral load) in Unspecified specimen by NAA with probe detection	LOINC
723476	SARS-CoV-2 (COVID19) RNA [Presence] in Nasopharynx by NAA with non-probe detection	LOINC
586526	SARS-CoV-2 (COVID19) RNA [Presence] in Nasopharynx by NAA with probe detection	LOINC
757677	SARS-CoV-2 (COVID19) RNA [Presence] in Nose by NAA with probe detection	LOINC
706163	SARS-CoV-2 (COVID19) RNA [Presence] in Respiratory specimen by NAA with probe detection	LOINC
715260	SARS-CoV-2 (COVID19) RNA [Presence] in Saliva (oral fluid) by NAA with probe detection	LOINC
715261	SARS-CoV-2 (COVID19) RNA [Presence] in Saliva (oral fluid) by Sequencing	LOINC
723463	SARS-CoV-2 (COVID19) RNA [Presence] in Serum or Plasma by NAA with probe detection	LOINC
706170	SARS-CoV-2 (COVID19) RNA [Presence] in Unspecified specimen by NAA with probe detection	LOINC
706158	SARS-CoV-2 (COVID19) RNA panel - Respiratory specimen by NAA with probe detection	LOINC
706169	SARS-CoV-2 (COVID19) RNA panel - Unspecified specimen by NAA with probe detection	LOINC
723467	SARS-CoV-2 (COVID19) S gene [Cycle Threshold #] in Respiratory specimen by NAA with probe detection	LOINC
723468	SARS-CoV-2 (COVID19) S gene [Cycle Threshold #] in Unspecified specimen by NAA with probe detection	LOINC

For peer review only

723465	SARS-CoV-2 (COVID19) S gene [Presence] in Respiratory specimen by NAA with probe detection	LOINC
586519	SARS-CoV-2 (COVID19) S gene [Presence] in Serum or Plasma by NAA with probe detection	LOINC
723466	SARS-CoV-2 (COVID19) S gene [Presence] in Unspecified specimen by NAA with probe detection	LOINC
586517	SARS-CoV-2 (COVID19) whole genome [Nucleotide sequence] in Isolate by Sequencing	LOINC
40218805	Testing for SARS-CoV-2 in CDC laboratory	HCPCS
40218804	Testing for SARS-CoV-2 in non-CDC laboratory	HCPCS

For peer review only

Supporting Table 3. Definitions and codes used for hypertension and other comorbidities

**Name**	**Included Codes**
Hyperlipidemia	https://atlas.ohdsi.org/#/concept/432867
Chronic kidney disease	https://atlas.ohdsi.org/#/cohortdefinition/312
Cancer	https://atlas.ohdsi.org/#/cohortdefinition/222
Asthma	https://atlas.ohdsi.org/#/cohortdefinition/218
Dementia	https://atlas.ohdsi.org/#/cohortdefinition/226
Total cardiovascular disease	https://atlas.ohdsi.org/#/cohortdefinition/246
Peripheral vascular disease	https://atlas.ohdsi.org/#/concept/321052

For peer review only - http://bmjopen.bmj.com/site/about/guidelines.xhtml

Cerebrovascular disease	https://atlas.ohdsi.org/#/concept/381591
Chronic liver disease	https://atlas.ohdsi.org/#/concept/4212540
Chronic obstructive pulmonary disease	https://atlas.ohdsi.org/#/cohortdefinition/219
Heart disease	https://atlas.ohdsi.org/#/cohortdefinition/231
Hypertension	https://atlas.ohdsi.org/#/cohortdefinition/227
Obesity	https://atlas.ohdsi.org/#/cohortdefinition/224
Type 2 Diabetes Mellitus	https://atlas.ohdsi.org/#/cohortdefinition/311

Supporting Table 4. Prevalence of hypertension among COVID-19 patients in the diagnosed and hospitalised cohorts in the CHARYBDIS Network.

	**Diagnosed with COVID-19**		**Hospitalized with COVID-19**	
	**N of prevalent cases**	**% (95%CI)**	**N of prevalent cases**	**% (95%CI)**
IQVIA-OpenClaims-US	1,245,436	48.3 (48.2-48.4)	384,508	76.5 (76.3-76.6)
OPTUM-EHR-US	66,432	37.4 (37.2-37.7)	18,242	64.1 (63.5-64.6)
VA-OMOP-US	34,093	61.4 (61.0-61.8)	8,996	85.9 (85.2-86.6)

For peer review only

For peer review only - http://bmjopen.bmj.com/site/about/guidelines.xhtml

HEALTHVERITY-US	25,405	22.3 (22.0-22.5)	4,512	59.5 (58.4-60.6)
SIDIAP-Spain	21,289	17.4 (17.2-17.6)	5,636	31.0 (30.3-31.6)
CUIMC-US	3,672	43.1 (42.1-44.2)	1,708	65.7 (63.9-67.5)
IQVIA-LPD-France	3,260	19.0 (18.4-19.6)	-	-
CU-AMC-HDC-US	2,461	33.9 (32.8-34.9)	904	63.0 (60.5-65.5)
IQVIA-DA-Germany	2,418	30.3 (29.3-31.3)	-	-
HIRA-South Korea	-	-	1943	25.6 (24.6-26.6)
IQVIA-LPD-Italy	1,618	36.1 (34.6-37.5)	-	-
STARR-OMOP-US	1,246	37.4 (35.8-39.1)	342	55.6 (51.7-59.5)
HMAR-Spain	-	-	594	29.5 (27.5-31.5)
CPRD-UK	756	22.4 (21.0-23.8)	-	-
IPCI-The Netherlands	676	22.2 (20.7-23.7)	-	-

For peer review only

Reporting checklist for cohort study.

Based on the STROBE cohort guidelines.

Instructions to authors

Complete this checklist by entering the page numbers from your manuscript where readers will find each of the items listed below.

Your article may not currently address all the items on the checklist. Please modify your text to include the missing information. If you are certain that an item does not apply, please write "n/a" and provide a short explanation.

Upload your completed checklist as an extra file when you submit to a journal.

In your methods section, say that you used the STROBE cohortreporting guidelines, and cite them as:

von Elm E, Altman DG, Egger M, Pocock SJ, Gotzsche PC, Vandenbroucke JP. The Strengthening the Reporting of Observational Studies in Epidemiology (STROBE) Statement: guidelines for reporting observational studies.

		Reporting Item	Page Number
**Title and abstract**			
Title	#1a	Indicate the study's design with a commonly used term in the title or the abstract	1
Abstract	#1b	Provide in the abstract an informative and balanced summary of what was done and what was found	4,5
**Introduction**			
Background / rationale	#2	Explain the scientific background and rationale for the investigation being reported	6
Objectives	#3	State specific objectives, including any prespecified hypotheses	6
**Methods**			
Study design	#4	Present key elements of study design early in the paper	6
Setting	#5	Describe the setting, locations, and relevant dates, including periods of	6-8

For peer review only

		recruitment, exposure, follow-up, and data collection	
Eligibility criteria	#6a	Give the eligibility criteria, and the sources and methods of selection of participants. Describe methods of follow-up.	6-8
Eligibility criteria	#6b	For matched studies, give matching criteria and number of exposed and unexposed	n/a
Variables	#7	Clearly define all outcomes, exposures, predictors, potential confounders, and effect modifiers. Give diagnostic criteria, if applicable	8,9
Data sources / measurement	#8	For each variable of interest give sources of data and details of methods of assessment (measurement). Describe comparability of assessment methods if there is more than one group. Give information separately for for exposed and unexposed groups if applicable.	8,9
Bias	#9	Describe any efforts to address potential sources of bias	n/a
Study size	#10	Explain how the study size was arrived at	8
Quantitative variables	#11	Explain how quantitative variables were handled in the analyses. If applicable, describe which groupings were chosen, and why	9-10
Statistical methods	#12a	Describe all statistical methods, including those used to control for confounding	
9-10			
Statistical methods	#12b	Describe any methods used to examine subgroups and interactions	n/a
Statistical methods	#12c	Explain how missing data were addressed	n/a
Statistical methods	#12d	If applicable, explain how loss to follow-up was addressed	n/a
Statistical methods	#12e	Describe any sensitivity analyses	
n/a			
**Results**			
Participants	#13a	Report numbers of individuals at each stage of study—eg numbers potentially eligible, examined for eligibility, confirmed eligible,	10

For peer review only

		included in the study, completing follow-up, and analysed. Give information separately for for exposed and unexposed groups if applicable.	
Participants	#13b	Give reasons for non-participation at each stage	10
Participants	#13c	Consider use of a flow diagram	
Supporting figure 1			
Descriptive data	#14a	Give characteristics of study participants (eg demographic, clinical, social) and information on exposures and potential confounders. Give information separately for exposed and unexposed groups if applicable.	10,11
Descriptive data	#14b	Indicate number of participants with missing data for each variable of interest	
n/a			
Descriptive data	#14c	Summarise follow-up time (eg, average and total amount)	
10			
Outcome data	#15	Report numbers of outcome events or summary measures over time. Give information separately for exposed and unexposed groups if applicable.	
11-16			
Main results	#16a	Give unadjusted estimates and, if applicable, confounder-adjusted estimates and their precision (eg, 95% confidence interval). Make clear which confounders were adjusted for and why they were included	n/a
Main results	#16b	Report category boundaries when continuous variables were categorized	Figures 1-4
Main results	#16c	If relevant, consider translating estimates of relative risk into absolute risk for a meaningful time period	
n/a			
Other analyses	#17	Report other analyses done—eg analyses of subgroups and interactions, and sensitivity analyses	n/a

Discussion

For peer review only

Key results	#18	Summarise key results with reference to study objectives	17
Limitations	#19	Discuss limitations of the study, taking into account sources of potential bias or imprecision. Discuss both direction and magnitude of any potential bias.	19
Interpretation	#20	Give a cautious overall interpretation considering objectives, limitations, multiplicity of analyses, results from similar studies, and other relevant evidence.	17-19
Generalisability	#21	Discuss the generalisability (external validity) of the study results	18-19
**Other Information**			
Funding	#22	Give the source of funding and the role of the funders for the present study and, if applicable, for the original study on which the present article is based	22,23

Notes:

- 13c: Supporting figure 1
- 16b: Figures 1-4 The STROBE checklist is distributed under the terms of the Creative Commons Attribution License CC-BY. This checklist was completed on 20. September 2021 using https://www.goodreports.org/, a tool made by the EQUATOR Network in collaboration with Penelope.ai

For peer review only
